# Nanoscopic subcellular imaging enabled by ion beam tomography

**DOI:** 10.1038/s41467-020-20753-5

**Published:** 2021-02-04

**Authors:** Ahmet F. Coskun, Guojun Han, Shambavi Ganesh, Shih-Yu Chen, Xavier Rovira Clavé, Stefan Harmsen, Sizun Jiang, Christian M. Schürch, Yunhao Bai, Chuck Hitzman, Garry P. Nolan

**Affiliations:** 1 Baxter Laboratory, Department of Microbiology and Immunology, Stanford University School of Medicine, Stanford, CA USA; 2 Department of Radiology, Molecular Imaging Program at Stanford, Stanford University School of Medicine, Stanford, CA USA; 3 Wallace H. Coulter Department of Biomedical Engineering, Georgia Institute of Technology and Emory University, Atlanta, GA USA; 4 School of Electrical and Computer Engineering, Georgia Institute of Technology, Atlanta, GA USA; 5 Department of Chemistry, Stanford University, Stanford, CA USA; 6 Department of Materials Science and Engineering, Stanford University, Stanford, CA USA; 7 Present Address: Department of Radiology, Perelman School of Medicine, University of Pennsylvania, Philadelphia, PA USA; 8 Present Address: Department of Pathology and Neuropathology, University Hospital and Comprehensive Cancer Center Tübingen, Tübingen, Germany

**Keywords:** 3-D reconstruction, Nuclear organization, Image processing, Single-cell imaging

## Abstract

Multiplexed ion beam imaging (MIBI) has been previously used to profile multiple parameters in two dimensions in single cells within tissue slices. Here, a mathematical and technical framework for three-dimensional (3D) subcellular MIBI is presented. Ion-beam tomography (IBT) compiles ion beam images that are acquired iteratively across successive, multiple scans, and later assembled into a 3D format without loss of depth resolution. Algorithmic deconvolution, tailored for ion beams, is then applied to the transformed ion image series, yielding 4-fold enhanced ion beam data cubes. To further generate 3D sub-ion-beam-width precision visuals, isolated ion molecules are localized in the raw ion beam images, creating an approach coined as SILM, secondary ion beam localization microscopy, providing sub-25 nm accuracy in original ion images. Using deep learning, a parameter-free reconstruction method for ion beam tomograms with high accuracy is developed for low-density targets. In cultured cancer cells and tissues, IBT enables accessible visualization of 3D volumetric distributions of genomic regions, RNA transcripts, and protein factors with 5 nm axial resolution using isotope-enrichments and label-free elemental analyses. Multiparameter imaging of subcellular features at near macromolecular resolution is implemented by the IBT tools as a general biocomputation pipeline for imaging mass spectrometry.

## Abstract (web summary)

Secondary ion beam mass spectrometry (SIMS) is a method to obtain a chemical snapshot of biological tissue, but the spatial resolution is low. Here, the authors develop a computational and technology pipeline to localise a chemical signal in SIMS in 3D and sub-25 nm accuracy, called Ion Beam Tomography

## Introduction

Multiparameter single-cell phenotyping for nucleic acids and proteins has revealed changes in molecular complexity that occur during development and throughout cancer progression in manners that reveal the underlying mechanism and potential therapeutics^1,2^. Localization within individual cells of biologic constituents with high-dimensional analytic techniques provides further depth to such understanding^3,4^. Multiplexed ion beam imaging (MIBI) and broadly secondary ion beam mass spectrometry (SIMS) can be employed to spatially visualize multiple (7–50) protein parameters in histology sections^5,6^. Since multiplexed fluorescence-based profiling methods have revealed identities of cells in tissues and suggested functions of novel cellular neighborhoods^7–9^, three-dimensional (3D) SIMS analysis should allow us to expand such neighborhood concepts to the subcellular realm.

Super-resolution microscopy^10,11^ and 4pi single-molecule microscopy^12^ have successfully been employed for 3D subcellular analysis. While these methods continue shedding light on cell biology, they are currently limited to a handful of parameters and face challenges due to the significant data-acquisition and alignment procedures for whole-cell mapping. Mass spectrometry offers unique advantages with its multiparameter and high-resolution imaging capabilities, and recent efforts in SIMS, specifically OrbiSIMS^13^, has demonstrated the potential of multiplexing by metabolic imaging, but the spatial resolution was limited to 300 nm. To date, primarily lipid profiling has been implemented with OrbiSIMS largely due to the sensitivity limits of given target analytes. Another implementation of SIMS, NanoSIMS imaging directly overcomes the optical diffraction limit^14–16^ and it has been used for direct assembly of sequential NanoSIMS scans for 3D representations of lipids^17,18^, but it has not been applied to date for error-reduced and high-precision (sub-ion-beam-width) whole-cell volumetric imaging to study subcellular organization.

Here we present ion beam tomography (IBT), a technical, reagent, and mathematical pipeline for analysis of ion beam images acquired from continuous depth scans of single cells. IBT achieves high-parameter multiplex detection (7 to potentially 50 markers), subcellular sub-25 nm lateral accuracy, and 5 nm axial resolution. To assess spatial resolution capabilities and sensitivity of IBT, we developed a suite of combinatorial reagents and deblurring algorithmic approaches to analyze subcellular structures and metabolites in single cells. Specifically, we applied IBT to the measurement of spatial distributions of nuclear architectural features, including replication forks, and newly synthesized and mature mRNAs in cultured cells. Subcellular tomographic data will enable visualization and quantification of normal and diseased intracellular states, and the synthesis of high-dimensional histology data with subcellular 3D mapping will intersect with basic biology and medical practice. Systematic and hierarchical subcellular organization of metabolic progression of single cells were consistent observations from the presented ion beam tomographic information cubes based on the computational association and spatially resolved 3D clustering analysis of isotopically tagged replication and transcription sites near chromatin.

## Results

### Design of ion beam tomography methods and analysis approaches

**Fig. 1:**
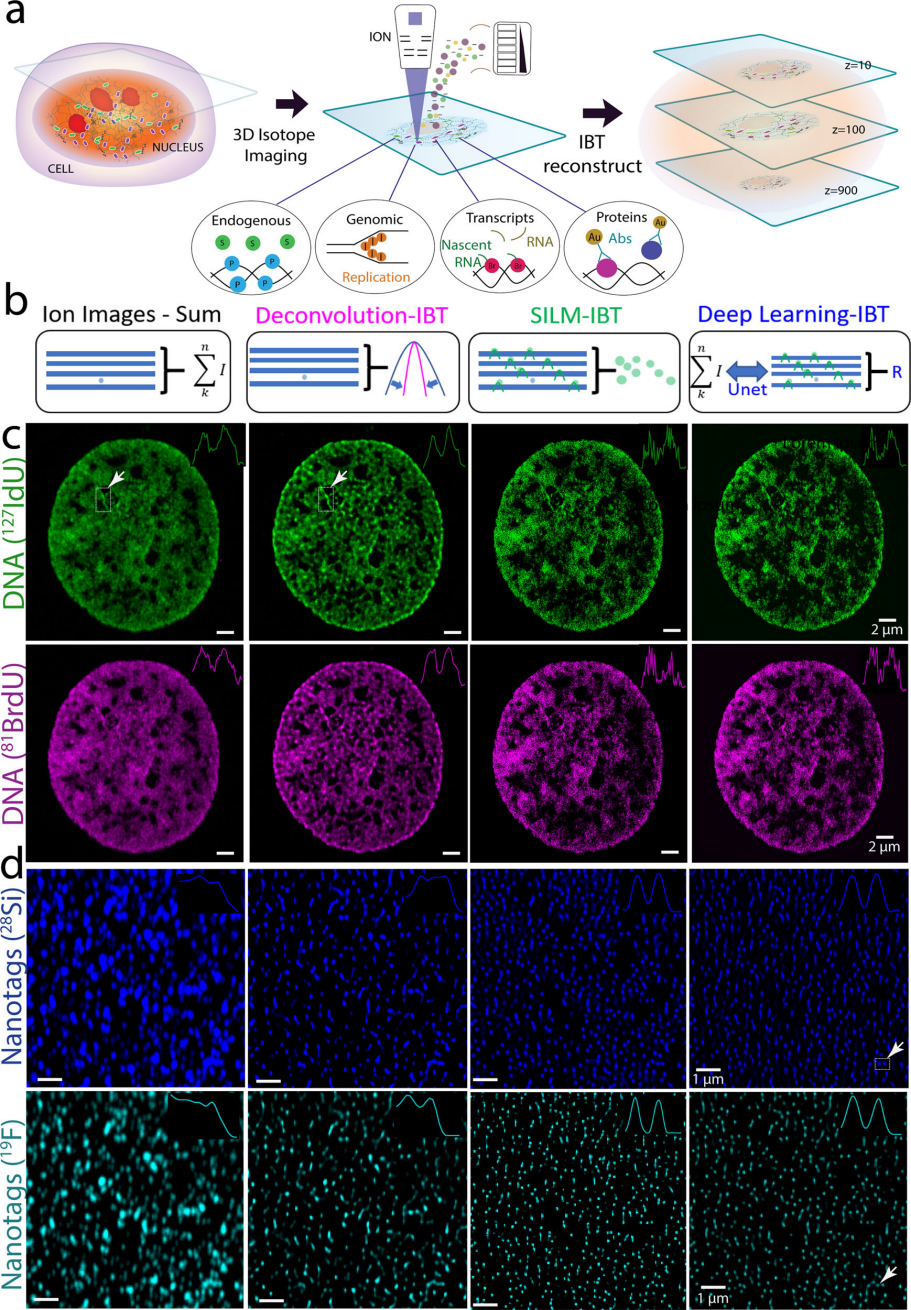
Ion-beam image reconstruction framework achieves high-precision imaging with multiplexing. **a** Metabolic labeling or metal-tagging in ion beam imaging. Continuous ion beam scans of up to 1000 depth sections were performed across the entire cell. **b** Reconstruction methods of serial image arrays from ion beam tomography (IBT) scans. Ion images-Sum**:** Conventional approach in SIMS image analysis, a range of 5-20 slices were directly summed. Deconvolution-IBT: A mathematical deblurring routine that iteratively removes out-of-focus etching. SILM-IBT**:** Localization of isolated ion molecules at each depth scan and synthesis of a highly precise ion image from localized ion distributions across 20–100 slices. Deep-learning-IBT**:** Training a neural net model on raw image stacks (low resolution) to SILM reconstructed image arrays (high precision) for deep learning-based reconstruction method to create a predicted image stack. **c** Co-tagged ^127^I-dU and ^81^Br-dU in a HeLa cell for chromatin mapping. (Top row) ^127^I channel in the SIMS data for newly synthesized DNA (^127^I-dU) after 24 h incorporation. Deconvolution resolves some of the overlapping chromatin features, while SILM and Deep-learning reconstructions finely resolve sub-25 nm chromatin fibers. The inset shows a cross-section across the DNA. Scale bars, 2 µm. (Bottom row) ^81^Br channels for the same DNA but now labeled with ^81^Br-dU simultaneously incubated with ^127^I-dU. Ion image arrays showed resolution enhancement from deconvolution to SILM and Deep-learning analysis, as before. **d** Comparison of image reconstruction methods by analyzing isotope encoded nanotags (100 nm diameters). ^19^F isotope was uniformly embedded in a Silica (^28^Si) matrix. (Top row) ^28^Si channel in the SIMS images for nanotags. The sum of the frames and Deconvolution-IBT resolve decent ion beam overlaps, and SILM and deep learning analysis allow sub-ion-beam-width reconstructions. Inset shows a cross-section of two neighboring nanotags, denoted by an arrow. Scale bars, 1 µm. (Bottom row) ^19^F channel in the same nanotags as a colocalized multi-color visuals. The SILM-IBT ^19^F and ^28^Si patterns agree well with each other with Structural similarity index (SSIM) values of 0.7. Reconstructions from Deep-learning-IBT and SILM provided SSIM values of 0.66. Inset corresponds to the resolved nanotag pair. Credit: Designua, Timonina, and Alejo Miranda/Shutterstock.com.

Ion-beam imaging enables visualization of the elemental composition of a cell specimen on a silicon substrate under bombardment with primary ions (Fig. 1a). A thin layer of cellular material (<5 nm) is etched during each raster scan, a process that generates secondary ions from the etched layer. The released ions can be analyzed by mass spectrometry to identify endogenous structures or labeled molecules. Two-dimensional (2D) scans of ion distributions have been previously used to produce spatial maps of cell materials at each layer.

Previous applications of MIBI utilized an oxygen duoplasmatron primary ion beam to generate 2D profiles of tissues at 260 to 500 nm spatial resolution with lanthanide isotopes as mass-based reporters utilizing a custom-MIBI-instrument based on SIMS^5,6^. Here, a different set of halogens, post-transitional and noble elements were used as contrast agents, and a cesium ion beam was employed using the NanoSIMS 50L device (Cameca), yielding 2D ion images at sub-100 nm lateral resolution due to the small spot size (<50 nm) of the beam, a superior capability of SIMS devices. Iterative scanning of a cell through the Z-axis generates multiple 2D images. Each 2D ion image series contains 3D information about the cellular volume since the ion beams extract ions slightly below (out of focus from) the ablated layer. While the stripped layer covers 5–10 nm at each scan, the penetration depth of ions extends to 100 nm^19^. This mismatch causes blurring in the acquired images, making it challenging to register and render images.

The standard approach for analyzing 3D ion image stacks involves the direct summation of images across the image stacks (Fig. 1b, Ion Images-Sum). Due to high noise levels and low-detection efficiency of SIMS imaging, the image sum approach is crucial to visualize continuous spatial distributions of subcellular structures. However, these images are still limited in their lateral resolution to the beam width of ions that are used for SIMS acquisitions. Practically, a physical ion-beam size of 50–100 nm has been used to capture SIMS images. For 3D whole-cell analysis, the beam size needs to be even larger (100–200 nm) to go through a 5–15 µm cell across 1000 slices. Therefore, achieving sub-ion-beam-width images is key to obtain high-precision ion tomograms.

We first developed a mathematical framework and a computational method to allow error-minimized 3D ion beam mapping of cellular volumes (Fig. 1b, Deconvolution-IBT). The resultant images were digitally assembled to generate volumetric renders of cells (Supplementary Fig. 1). Synthetic ion beam data were first used to validate the IBT approach (Supplementary Fig. 2). In the image used for validation, the true object distribution was a synthetic array of six virtual molecules separated by 200 nm and modeled as digitally acquired by a 120 nm ion beam width and 50 nm scanning pixel size on x and y axes (Supplementary Fig. 2, step 1). Only multiplicative noise is significant in ion beam imaging due to the independent fluctuations of ion pixel values for each frame; it is present as a gamma distribution and was calculated from a sample image acquired at similar ion imaging parameters. The convolved image was then contaminated by multiplicative noise factors. The digitally created images at distinct depths (*z*_*1*_, *z*_*2*_, and *z*_*3*_) were typical of noisy data (Supplementary Fig. 2, step 2). These multiple depth images were binned over a sliding window of size *n* that was varied between 3 and 20 slices (Supplementary Fig. 2, step 3). This process was then incremented by one layer at a time. For instance, ion signals from 1 to *n* slices were combined to yield the first transformed image, ion signals from slices 2 to *n* + 1 were binned for the second transformed image, and slices 3 to *n* + 2 yielded the third transformed ion image without the loss of axial resolution. This rendered an image series of 1000 slices into a restructured image series of a similar length (slightly shorter, reduced only by the sliding window size). Each binned section was then digitally deblurred by an iterative Lucy–Richardson^20^ deconvolution method (Supplementary Fig. 2, step 4). The Lucy-Richardson algorithm is suitable for experimental point-spread function (PSF) and iterative analysis; however, changes in electric current levels due to the ion source quality and parameters of the ion beam imaging system make it complicated to measure a unique PSF for every ion image of interest. To address this problem, a “hybrid” deconvolution algorithm was created by using a blind PSF rather than an experimentally determined PSF. The width of the blind PSF is selected based on the numerical value of the ion current recorded before and after the ion beam scanning. The width of the blind PSF is typically in the 100–200 nm range; for the simulation described, the PSF was 120 nm. The final ion image showed six distinct signatures that agreed well with the synthetic input.

Deconvolution-IBT was then applied to cellular metabolic signatures of DNA. A HeLa cell was simultaneously tagged with 5-^127^iodo-2′-deoxyuridine (^127^I-dU) and 5-^81^bromo-2′-deoxyuridine (^81^Br-dU) for 24 h to visualize replicated DNA. In principle, these two images correspond to the same chromatin pattern due to the incorporation of the same base (thymidine) into the DNA, making it ideal for comparative analysis of digital treatments. In comparison to the summation of 20 slices from this DNA image, deconvolved DNA signatures provided finer details (Fig. 1c). Insets show line scans with sharper peaks corresponding to a smaller DNA piece pointed by an arrow, after deconvolving and plotting a cross-section of the DNA patterns in both ^127^I-dU and ^81^Br-dU channels.

To characterize resolution enhancements of deconvolution-IBT, fabricated metal-coated silicon substrates with a sharp interface of gold (^197^Au) and silicon were imaged at different current levels. Using the deconvolution analysis pipeline, 55 nm lateral resolution was obtained using a cesium source from three edge scans (Supplementary Figs. 3-4). The deconvolution routine improved the resolution up to 4-fold in the *x–y* axis of IBT images. Here, the resolution was defined based on the error function (erf) fit^21^ of the signal drop at the edge, corresponding to the distance between 88 and 12% of the maximum ion beam signal at the plateau region. With these settings, a presumptive 53 nm resolution image was obtained for ion beam images of a Jurkat cell (Supplementary Fig. 5). Using the oxygen source, only a 498 nm maximum resolution was obtained from three edge scans at the junction of aluminum and silicon (Supplementary Fig. 6). Thus, the resolution depends on ion current levels and aperture size. As expected, a cesium beam allowed for higher resolution imaging than an oxygen beam. Therefore, with a scalable resolution from 55 nm to 500 nm, ion beam imaging bridges a gap between super-resolution optical imaging and wide-field microscopy (Supplementary Fig. 7). The axial depth resolution of the ion beam imaging was evaluated by two approaches. In the first, a single replication site that was marked by 30 min incorporation of ^127^I-dU was evaluated: A 175 nm wide pattern crossed 40 slices, indicative of 5 nm axial resolution (Supplementary Figs. 8 and 9). In the second approach, the cell height (~10 µm) was divided by the total number of scans (1000), again indicative of 5–10 nm axial resolution (Supplementary Fig. 10).

To create high-precision IBT maps with sub-ion-beam-width accuracy, we were inspired by the stochastic optical reconstruction microscopy (STORM) method^22,23^ from fluorescence imaging. STORM utilizes blinking single molecules to localize individual fluorophores and combines the localized positions to synthesize super-resolved data. In ion beam imaging, we adopt each scan from ultra-thin layers (1–10 nm) as a single time-stamped frame with varying ion signal levels and combine localized ion images from a series of sequential scans to generate a high-precision IBT reconstruction (Fig. 1b, SILM-IBT). We term this approach Secondary Ion-beam Localization Microscopy (SILM) that provides nanoscopic ion beam data cubes from a series of NanoSIMS scans (Supplementary Fig. 11). The highly precise ion-beam analysis benefits from the stochastic nature of a subset of ion signal levels appearing at each SIMS image, allowing us to localize only a subset of ion molecules. Unlike the STORM method, only mathematical localizations (similar to the FIONA^23^ approach) of individual molecules were used as a general spatially resolving principle, as also previously performed in photoacoustic imaging^24^ and label-free interferometric imaging^25^. Using a sliding window of 20–100 frames, the depth resolution is retained back to achieve highly precise tomograms of single cells. The high-precision tomograms, however, can be applied to mostly relatively dense subcellular markers and structures. For instance, a concentrated DNA image in the ^127^I-dU and ^81^Br-dU channels were analyzed by the SILM-IBT pipeline. Insets show the line scans with precisely localized features of chromatin in the major peaks identified in the previous sum and deconvolution IBT images denoted by dashed squares (Fig. 1c).

To quantify and validate the performance of SILM-IBT, we designed and fabricated multi-color nanotags with a 109.8 ± 17.6 nm average diameter (Supplementary Fig. 12), akin to TetraSpeck microspheres^26^ used in fluorescence microscopy. These nanotags are composed of homogeneously distributed isotopes (^19^F) within a silica matrix (^28^Si) across the entire 109 nm spherical volume, making them an appropriate model for generating ion image stacks to be tested by SILM analysis. An advantage of this approach is to colocalize the same nanotags in two or more channels of the ion beam imaging as a validation dataset without the need for separate scanning electron microscopy (SEM) comparisons. As an initial characterization, we imaged and analyzed ^19^F isotope embedded silica nanotags (Fig. 1d). Systematic improvements from Deconvolution-IBT and SILM-IBT were reproducibly obtained from nanotags signatures in both ^19^F and ^28^Si channels. The cross-sections of neighboring nanotags were finely resolved in the SILM data, but not in the deconvolution-IBT or the sum of the stacks (Supplementary Fig. 13). The precision accuracy of localized ions yielded 25 nm spatial localization accuracy of SILM for 3D ion beam imaging (Supplementary Fig. 14). Theoretically, SILM-IBT can achieve down to 5 nm lateral accuracy, given the sub-50 nm ion beam size and sufficient ion counts (Supplementary Fig. 15). Deconvolution-IBT revealed continuous structures in subcellular volumes to perform structural comparisons of connected molecular signatures at sub-55 nm resolution. On the other hand, SILM-IBT further localized sub-25 nm ultrafeatures in subcellular structures to study local molecular density such as the origins of replicated DNA and super-compact DNA structures. Deconvolution-IBT and SILM-IBT are two independent algorithms that use raw ion image stacks to generate deblurred ion reconstructions. As a comparison, nanotag pairs in the ^19^F channel were not resolved in the raw IBT images but finely separated in the SILM-IBT reconstructions, providing a key validation to the presented SILM-IBT platform (Supplementary Fig. 16).

As a general tomographic reconstruction strategy, we utilized deep learning analysis^27,28^ for IBT data (Fig. 1b). In this method, a model was trained from raw ion images (low-resolution) to SILM-IBT data (high-precision). An ion beam image of interest, acquired at similar conditions, was then processed to predict a spatially enhanced version of the corresponding ion beam slice using the training model (Supplementary Fig. 17). Applying deep learning reconstructions one slice at a time allowed the 3D assembly of whole-cell tomograms. The performance of deep learning analysis was evaluated with multi-color nanotags and co-labeled DNAs (Fig. 1c, d). Cross-sections yielded deep-learning-IBT reconstructions that are higher resolution than deconvolution-IBT and comparable to SILM-IBT. The advantage of the deep learning-IBT method is to create highly precise tomograms even at low-density biological structures, whereas SILM-IBT requires relatively high ion signals that consistently appearing in subsequent slices.

To evaluate the reconstruction performance for these three IBT analysis approaches, a structural similarity index (SSIM) method was used to compare the quality of a reference image to a target image^29^. Initially, the effect of Deconvolution-IBT was quantified from presumably similar ^127^I-dU and ^81^Br-dU ion images of an individual HeLa cell. Although spatial distributions of pixels in raw ion images for each depth were significantly dissimilar (SSIM, value: 0.54, Fig. 1c and Supplementary Fig. 18), deconvolved-IBT ion images were highly correlated (SSIM value: 0.86, providing a validation of the presented deconvolution processing methods. The same digital enhancement was obtained in Nalm6 cells from the SSIM value of 0.74 to 0.88 for Deconvolution-IBT (Supplementary Fig. 19). Next, SILM-IBT and Deep-Learning-IBT results were assessed from ^19^F-^28^Si dual-labeled nanotags, which should colocalize owing to the uniformity. SILM-IBT reconstructed images of nanotags in the ^19^F and the second ^28^Si channel showed similar spatial nanotag distributions (SSIM value, 0.7), validating the mass-channel-independent performance of localizations in ion beam imaging. Finally, Deep-learning-IBT-based predictions of nanotag distributions agreed well (SSIM value, 0.66) with the SILM-IBT reconstructions of nanotags. These SSIM value ranges fall into a typical range of deep learning-based enhancement of images that were set in the prior imaging works^27^.

IBT image acquisition settings must be carefully adjusted to obtain accurate reconstructions. Images are acquired at a low current level (<4 pA) for high-resolution analysis, yielding ion beam raw images at sub-100 nm resolution. Pixel size dimensions, *∆x* and *∆y*, of these ion beam images, must be smaller than the ion beam width of ∆w (Supplementary Figs. 20–21). To reconstruct these finely sampled experimental images, a PSF width between 100 and 200 nm was utilized in the deconvolution process. SILM analysis used this PSF for localizing individual ions. The same PSF width was also utilized in the training and prediction steps of the deep-learning-based reconstructions. Another parameter that affects the sensitivity in the ion beam imaging is ion conversion efficiency; this is defined as the ratio of ion incoming signal (*P*_in_) to the total secondary ion extracted from the sample (*P*_out_), and was calculated to be 3%. The captured ion beam images are typically fuzzy due to the finite ion beam width, imperfect image focusing, and out-of-focus etching. Therefore, multiple depth combinations were used to enhance the signal-to-noise ratio (Supplementary Fig. 22) for deconvolution analysis. SILM analysis and predictions from neural networks were used to transform raw images into high-precision ion beam image reconstructions.

Optical microscopy often suffers from multi-color aberrations that limit the co-localization of labeled regions. IBT benefits from the simultaneous etching of the same pixel regions in the channel of interest without any lateral shifts. Corrections are required, however, due to general image offset during depth scans. To enable these corrections, a fiducial pattern is required that allows the detection of spatial shifts of each depth image. To provide this pattern, iron (^55^Fe) microparticles with a diameter of 2–6 µm were added to cell samples, and the center and edge of the particle were tracked during the ion scans to allow image shift corrections (Supplementary Fig. 23). These shift values were then applied to other mass channels creating error-reduced, 3D cellular maps.

Subcellular IBT experiments provide tomographic, continuous acquisition of multiple depths in a few cells per day. To increase the throughput of the IBT approach, a chained mode was used to image at high-low currents by alternating image acquisition (Supplementary Fig. 24). In this strategy, a high-resolution scan was followed by a low-resolution scan, and this process was repeated until the bottom of the cell was reached. Chained mode preserves lateral resolution at sub-100 nm spatial details, but it loses IBT’s high axial resolution as the low-resolution scans etch through deeper (more than 50 nm) cellular layers. At the expense of spatial resolution, a higher current can image through the entire cell in a shorter timeframe to increase cell numbers evaluated (Supplementary Fig. 25).

### Tagging, labeling systems, and post-processing of IBT data

**Fig. 2:**
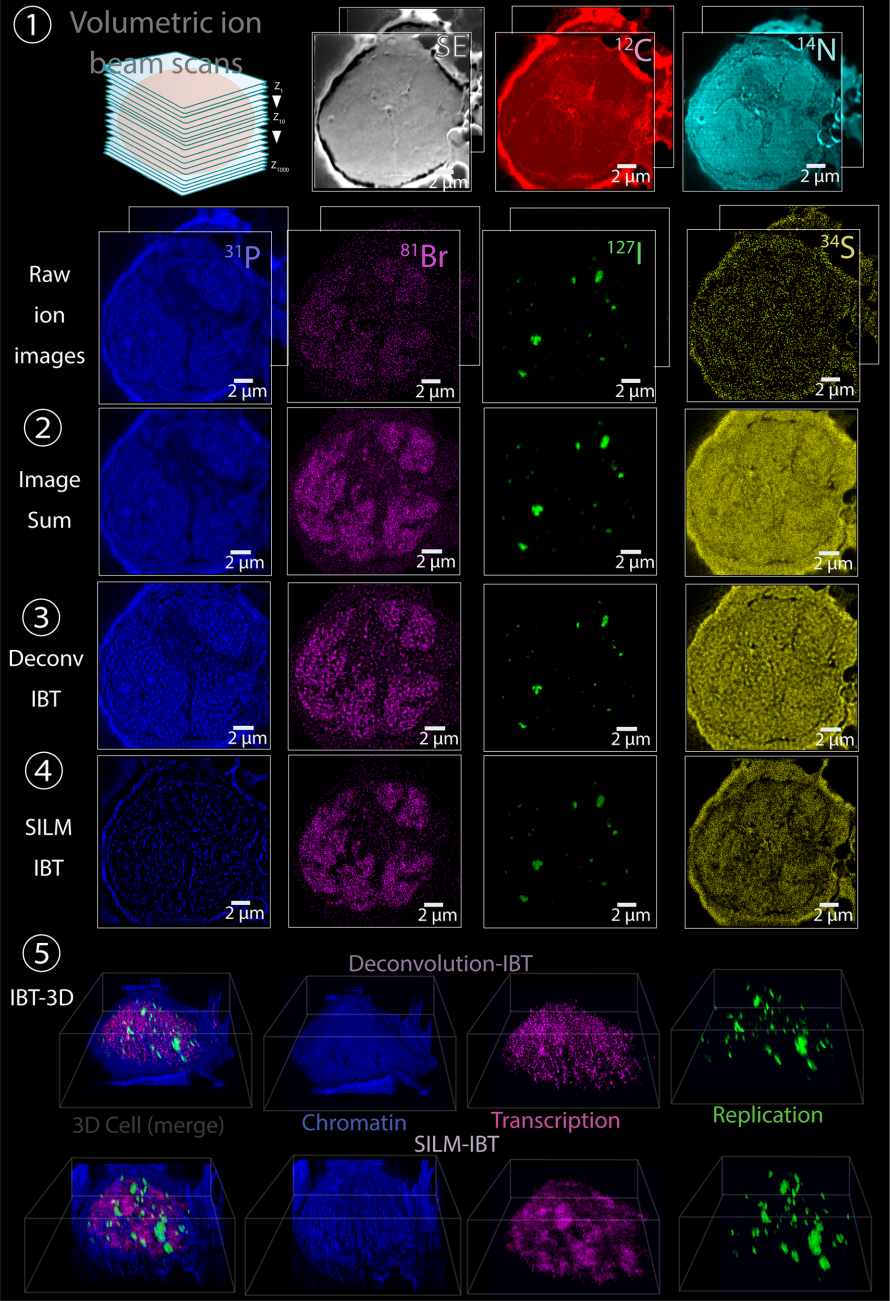
Metabolic tagging and endogenous elemental contrasts enable ion beam nanoscopic tomography. A single Nalm6, B cell lymphoblast, imaged for Secondary electron (SE), ^12^C (red), ^14^N (measured as ^12^C^14^N, cyan), ^31^P for DNA (blue), ^81^Br for newly synthesized transcripts (labeled with ^81^Br-rU, magenta), ^127^I for replication loci (labeled with ^127^I-dU, green), and ^34^S (yellow) for detection of total proteins. ① A volumetric scan across 1000 slices was performed by ion beam imaging from the top to the bottom of a cell for seven mass channels. Potentially 100 distinct mass channels can be analyzed. Raw ion images showed highly pixelated and noisy ion beam data. Scale bar 2 µm. ② Standard image sum analysis of 20 ion stacks for four mass channels. Global spatial patterns in subcellular volumes were visible albeit being blurry. Scale bar 2 µm. ③ Deconvolution-IBT analysis of four channels resolved finer structures in total DNA (blue), RNA (magenta), replicated DNA (green), and proteins (yellow). Chromatin showed folding fiber structures and other channels exhibited subcellular distributions around chromatin. Scale bar 2 µm. ④ Highly precise IBT using SILM analysis provided even sharper ion reconstructions that generated spatially localized data that is more precise than the practical resolution limit of ion-beam-width. Localized ion signals from each channel were digitally combined across twenty ion slices to create a tomographic image. Scale bar 2 µm. ⑤ Ion-beam tomograms are shown in the deconvolution-IBT image format (upper images, *n* = 600 stacks) and SILM-IBT image arrays (lower images, *n* = 600 stacks) with the three channels (selected out of seven acquired channels) for combining as a 3D cell, in the form of chromatin, transcription, and replication tomographic representations. 3D Visualization tool of *Volocity* (Perkin Elmer) was used to render IBT data in 3D.

Previous MIBI experiments utilized mostly lanthanides as reporters for tissue-labeling experiments and oxygen or gold ion beam sources (Fig. 2). For IBT, a different set of elemental tags were required to enable higher contrast levels when imaged by a sub-100 nm spot size of a cesium source. Sensitivities were based on the efficiency of secondary ion beam generation (Supplementary Fig. 26). Previous high-resolution ion beam imaging experiments have relied on metabolic enrichment of ^2^H, ^13^C, ^15^N, and ^18^O to measure turnover rates and to monitor cell division^14,15^. As a proof of concept for the IBT workflow, the following were detected in Nalm6 cells using a cesium beam: ^12^C as a measure of carbon distribution, ^14^N (measured as ^12^C^14^N) as a measure of nitrogen distribution, ^34^S as a measure of total protein, ^31^P as a measure of nucleic acids, ^127^I-dU for labeling of replication sites, and 5-^81^Br-ribouridine (^81^Br-rU) for labeling of newly synthesized transcripts (Fig. 2, step ①). Raw images were improved by multi-slice image sum (Fig. 2, step ②), and digital deconvolution of binned images (Fig. 2, step ③). Raw images were also directly localized by SILM analysis for sub-ion-beam-width reconstructions (Fig. 2, step ④). After performing these steps for each ion beam depth image, 3D representations in the form of analog pixels values (equivalent to 3D cell tomograms in terms of chromatin, replication, or transcription) and surface-rendered volumetric reconstructions were created from Deconvolution-IBT and SILM-IBT image stacks, wherein highly precise images have yielded much finer details of ion tomograms (Fig. 2, step ⑤).

Ion-beam tomograms provide snapshots of subcellular structures; they do not provide direct measures of molecular changes over time. For instance, after a 30 min incubation with ^127^I-dU, cells are fixed, and the moment just before fixation is preserved and measured. To enable capture of dynamic information with ion imaging time-dependent labeling can be used during the sample preparation. To analyze replication dynamics, cells were sequentially pulsed with ^127^I-dU and ^81^Br-dU with chase times of 30 min or 2 h, followed by immediate fixation and ion beam tomographic analysis. For transcription and replication co-dynamics analysis, cells were simultaneously pulsed with ^81^Br-rU and with ^127^I-dU for 30 min, followed by fixation and ion beam imaging.

After the application of the ion current-dependent blind PSF to iteratively compute the sharpened image, the final ion images are visualized in the form of volumetric renders, 3D spatially resolved clustering, and molecular neighborhood maps. As in analyses of the 2D data obtained from histology and of the high-parameter single-cell mass cytometry data^1,5^, analysis of 3D ion beam data requires dimensionality reduction from pixel positions (*x*, *y*, and *z*). Our lab recently demonstrated the existence of novel organized cell neighborhoods related to tissue function in the immune system^9^. In the next section, such spatial network concepts were implemented to study chromatin interactions with other molecular types such as proteins in complex tissue samples. To further study subcellular structural organization in ion beam tomographic information cubes, spatially resolved 3D clustering, hierarchical analysis, correlation, and association maps were generated across subcellular patterns with various metabolic labeling conditions.

### IBT reveals 3D nuclear architecture, metabolites, and dynamics

Chromatin has multiple levels of super-structural complexity from 11 nm DNA-bound mono-nucleosomes to 30, 120, and 700 nm fibers^30^. Mono- or di-parameter direct visualization of higher-order chromatin details has been performed by super-resolution optical microscopy (STORM, spatial details: 30–100 nm) and enhanced electron microscopy (ChromEM, spatial details: 5–100 nm)^31,32^. Tomographic imaging of chromatin remains difficult, however. Optical microscopy requires many frames per cell and electron microscopy requires physical sectioning of cells; both pose problems for 3D registration. In contrast, IBT directly records spatial details of chromatin at sub-100 nm resolution, with multiple parameters, without the need for sectioning or multiple frames per depth. Chromatin 3D conformation was analyzed in the IBT results to define “global” and “local” distributions of DNA.

**Fig. 3:**
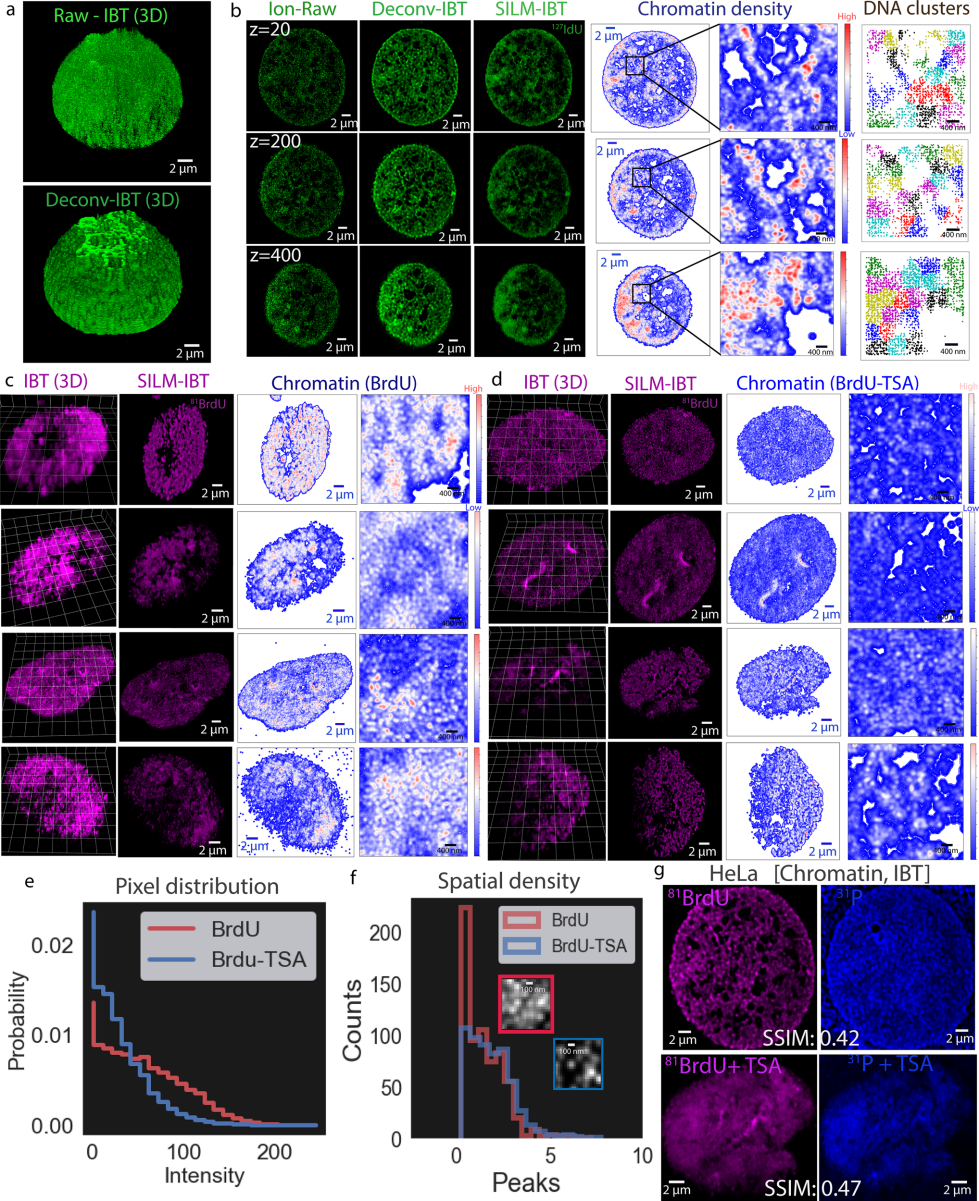
IBT for assay of chromatin states. **a** Thymidine analogue ^127^I-dU was simultaneously incorporated into HeLa cells for 24 h for chromatin visualization as a replicated DNA signature. IBT reconstructions and deconvolution IBT stacks were represented as 3D renders for 700 tomographic slices. **b** Replicated DNA images from ^127^I-dU. The first column shows typical raw ion images from 20^th^, 200^th^, and 400^th^ depths from the ^127^I-dU channel (green). The second column presents reconstructed ^127^I-dU images by Deconvolution-IBT. The third column corresponds to SILM-IBT images of the same chromatin target and these images were then converted to chromatin density maps (blue to red: low to high). The fourth and fifth columns (zoomed out version) show corresponding chromatin density maps. DNA clusters (*n* = 10) were assigned to unique colors on the spatial chromatin maps. **c** HeLa cells were treated with a histone deacetylase (HDAC) inhibitor, Trichostatin A (TSA), for 24 h, after labeling of replicated DNA for 24 h by ^81^Br-dU. Four HeLa cells were only labeled by ^81^Br-dU without TSA and up to 30 slices were acquired by SIMS. **d** Another set of four HeLa cells were treated by TSA and imaged. Chromatin density maps were created in the SILM-IBT images from these four cells. The cells that are treated with TSA consistently showed dimmer ion signals and more relaxed DNA features. **e** Intensity analysis of chromatin (BrdU, *n* = 4) and TSA treated chromatin (BrdU-TSA, *n* = 4) for an average of all the eight cells, yielding lower signal levels for TSA treated cells. **f** Spatial density analysis for peak distributions in a pair of cells with and without TSA, a reduction in the counts of peaks was detected. Insets: 20 × 20 pixels from each BrdU and BrdU-TSA images. **g** Chromatin features in a HeLa cell imaged using ^31^P channel together with ^81^Br-dU labeling for replicated DNA. Upper images: HeLa cell imaged using the IBT data from ^81^Br-dU and ^31^P channels, exhibiting 0.42 SSIM values. Lower images: Another HeLa cell for the same ^81^Br-dU and ^31^P channels after TSA treatment. IBT-sum images of 30 frames for ^81^Br-dU and ^31^P, providing SSIM values of 0.47.

For analysis of local chromatin density, replicated DNA was detected based on ^127^I-dU and ^81^Br-dU incorporation into the newly synthesized chromatin and processed by Deconvolution-IBT and SILM-IBT (Fig. 3a and Supplementary Fig. 27). In this experiment, HeLa cells were cultured with ^127^I-dU and ^81^Br-dU for 24 h, followed by fixation, and ion beam tomographic imaging across 700 depths. Raw ion images and reconstructed ion tomographic slices are presented for 20^th^, 200^th^, and 400^th^ slices in Fig. 3a, b. To map out finer chromatin patterns, SILM-IBT results were processed by density variations. In the contour maps, the entire cell was plotted for a continuous colormap: low (blue), medium (white), and red (high). Localized ion distributions were then clustered by nearest neighbor distances^31^. Each color shows a unique chromatin domain in the single cell, yielding spatially variant DNA densities in the range of 500–900 nm sized clusters.

To explore the effect of epigenetic perturbations, cells were treated with a histone deacetylase (HDAC) inhibitor, Trichostatin A (TSA). After incubating the HeLa cells with I-dU and Br-dU for 24 h, TSA was added to the same cells for another 24 h. Cells were fixed right after the treatment and imaged by SIMS. Eight HeLa cells were analyzed in the Br-dU channel for DNA density analysis, in which half of the cells were left untreated with TSA (Fig. 3c) and another half was treated with TSA (Fig. 3d). Spatial DNA distributions showed more relaxed and weaker patterns in the TSA-treated cells compared to the untreated ones. TSA reduced the chromatin intensity and DNA peaks in spatial density (Fig. 3e, f) based on the quantification of local DNA voxels (*n* = 4 treated, *n* = 4 untreated cells), providing statistical significance with a *P*-value of lower than 0.001 (***). Of note, these ion signal comparisons would get affected by local environmental changes that lead to concentration-independent variations in secondary ion signals, causing deviations in the precision of IBT-based spatial analysis of subcellular structures.

As a label-free DNA detection method, chromatin was independently identified by analysis of the ^31^P signal. Since all nucleic acids, as well as phosphorylated proteins, contribute to the endogenous ^31^P signal, the phosphate channel image differs slightly, as expected, from the ^127^I-dU and ^81^Br-dU images. Even though ^31^P is present in additional molecular entities, this image agreement (SSIM: 0.47) demonstrates that the endogenous ^31^P channel can be used to identify chromatin architecture without the need for additional metabolic or secondary tagging (Fig. 3g).

For global analysis of chromatin density, replicated DNA in the I-dU and Br-dU channel images were digitally segmented and quantified across the cell tomograms. Cancer cells and aberrant immune cells (B cell lymphoblasts) typically have large and deformed nuclei with protein compositions and chromatin conformations distinct from those of normal cells;^33,34^ very little is known about the subcellular DNA density in these abnormal cells. To resolve spatial details in chromatin density states, HeLa cells and Nalm6 cultures were incubated with ^127^I-dU and ^81^Br-dU to label replicating DNA, and signals from ^127^I, ^81^Br, and ^31^P channels were analyzed. Spatial subregions were defined by the spatial distribution of these signals. A direct calculation of signal intensity may be used to segment the chromatin; however, a fuzzy logic image segmentation (See “Methods”) was used for this analysis to consistently classify voxel distributions into subgroups of chromatin density states regardless of the dynamic range differences of the ion beam image values.

Chromatin states were defined as compacted (a largely inactive but dense state of chromatin), decondensed (regions of medium density chromatin), and very low density (regions with little active transcription)^35^. A spatially resolved color visualization of an individual Nalm6 cell for the 50^th^ ion beam image slice and HeLa for the 500^th^ ion slice showed chromatin in all three states (Supplementary Figs. 28–29). To evaluate the effect of image deconvolution on chromatin segmentation, raw and reconstructed images were segmented. The raw images exhibited smaller amounts of compact chromatin and higher amounts of very-low-density chromatin compared to the segmented results from reconstructed data (Supplementary Fig. 30a–d). These results demonstrate how deconvolution-IBT improvements can spatially resolve subcellular structures in ion beam images. Volumetric density analysis of a HeLa cell and a Nalm6 cell revealed “cell-type-specific” spatial nuclear maps of chromatin states (Supplementary Fig. 30e). Specifically, HeLa cells showed more decondensed chromatin compared to the Nalm6 cells.

**Fig. 4:**
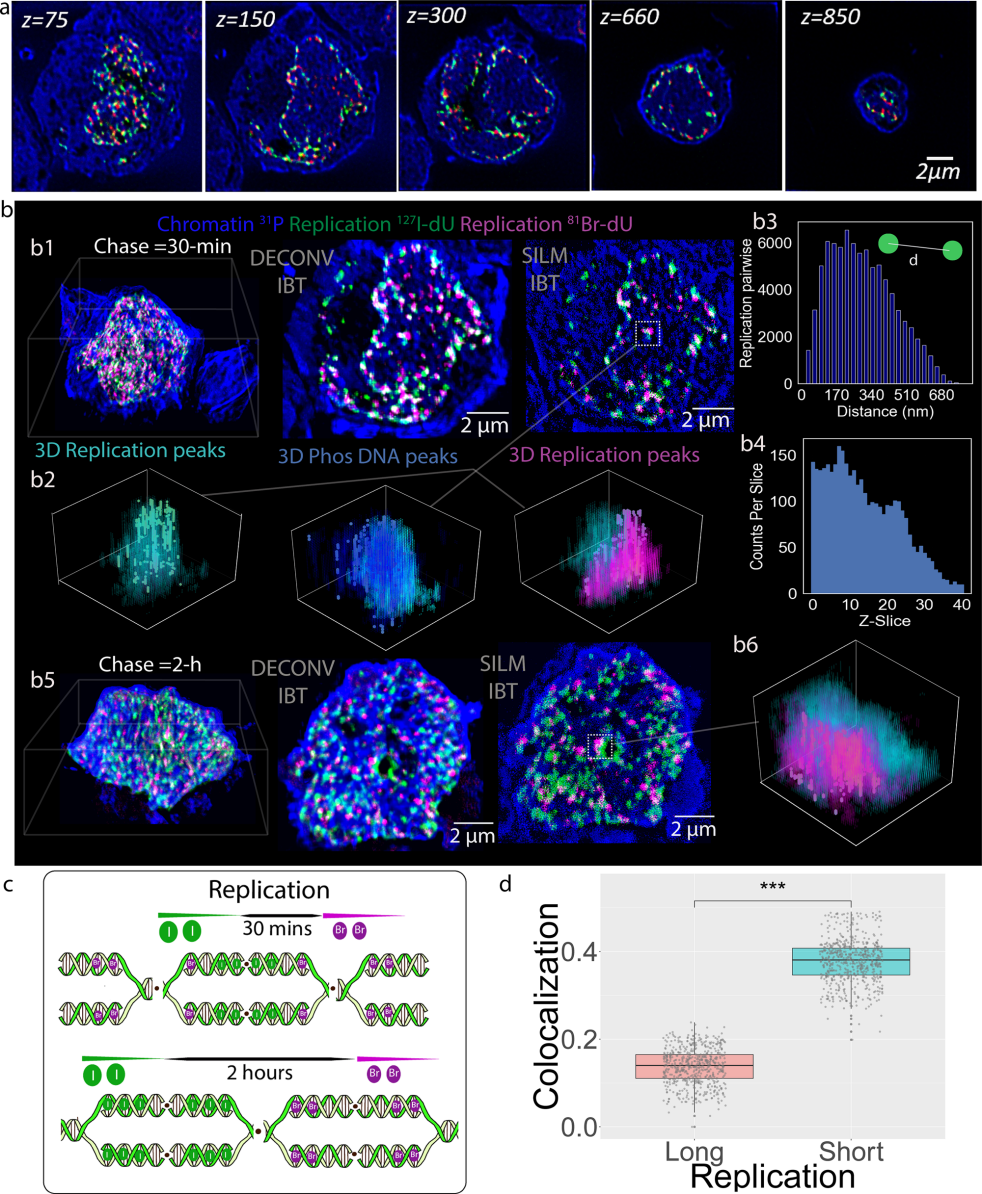
Ion-beam tomograms reveal overlaps and domains in the 3D distribution of DNA replication sites. **a** Nalm6 cells were incubated with ^127^I-dU for 30 min and then without a label for 30 min or 2 h, followed by a 30 min incubation with ^81^Br-dU. The endogenous DNA backbone was visualized in the ^31^P channel (blue) and replication sites were detected in the ^127^I-dU (green) and ^81^Br-dU (red) channels. IBT images are shown from the 75^th^ slice to the 850^th^ slice of the cell. **b** Upper images: 3D visualization of ^31^P (blue), replication sites labeled with ^127^I-dU (green), and replication sites labeled with ^81^Br-dU (magenta) across 1000 slices with a 30 min chase and 2 h chase. **b1** The 3D spatial distributions across 100 tomographic slices of replication sites for Deconvolution-IBT and SILM-IBT are illustrated. **b2** In the SILM-IBT, peaks were identified in a single replication site and shown as green circles for ^127^I-dU replication, blue circles for phosphorus, and magenta circles for ^81^Br-dU replication. **b3** Histogram of pairwise distances between the ^127^I-dU replication peaks, yielding an average 170 nm separation with a wide range extending to 600 nm. **b4** Peak counts per slice in the ^127^I-dU channel across 40 slices. Lower images: 3D render of ^31^P (blue) across 800 slices. **b5** In the middle and right images, the spatial distributions across 100 slices are presented for chromatin with^31^P (blue), replication sites labeled with ^127^I-dU (green), and ^81^Br-dU (magenta) in the form of Deconvolution-IBT and SILM-IBT renders, respectively. **b6** Peaks identified in the ^81^Br-dU channel overlaid with the original values of both ^127^I-dU and ^81^Br-dU replication voxels. **c** Schematic of the shorter chase time (30 min) for the replication site, leading to higher spatial overlap than the longer chase. **d** Box plot of co-localization of ^127^I-dU and ^81^Br-dU channels for the 2 h (long) and 30 min (short) chase experiments. Median, first and third quartile, and 95% confidence interval of the median are shown. Each data point corresponds to co-localization values per ion beam depth (*n* = 600 slices over a long IBT scan), providing a significant *p*-value < 2.2e-16 (***) by the *Wilcox* test (Two-sided).

High-resolution imaging of replication is crucial to understanding the metabolic effects of therapies in cancer, particularly for drug resistance studies^36^. To visualize replication forks, Nalm6 cells were incubated with ^127^I-dU for 30–60 min, then for 30 min or 2 h (the chase) without the label, and then with ^81^Br-dU for 30 min. Cells were fixed, and the subcellular ion beam images were collected (Fig. 4a). These ion beam image series were then converted with a 3D renderer (Fig. 4b). Images of the cells chased for 30 min showed significant overlaps in the ^127^I-dU and ^81^Br-dU channels (Fig. 4b1). Pulse-chase experiments performed in S-phase cells (synchronized with aphidicolin) revealed similar spatial overlap patterns^37^ (Supplementary Figs. 31–34). Peak detection analysis (Supplementary Fig. 35) in the SILM-IBT data of replication sites revealed separation distances in 3D of replication domains (Fig. 4b2), yielding an average distance of 170 nm and a wide range up to 600 nm (Fig. 4b3). Peaks of replication sites also exhibited heterogeneity and a dynamic range of up to 150 replication domains (RD) per ion beam slice (Fig. 4b4). IBT visuals of the cells chased for 2 h showed fewer overlaps in the ^127^I-dU and ^81^Br-dU channels (Fig. 4b5) and the corresponding 3D separation distances of replication domains were visualized (Fig. 4b6). These results suggest that with the short chase, ^81^Br-dU labels replication sites that are also labeled by ^127^I-dU, whereas the longer chase provides higher spatial separation of replication sites (Fig. 4c). The correlation coefficient (CC) of ^127^I-dU and ^81^Br-dU channels across 600 depths were calculated to quantify the overlap in the signal. The CC was 0.38 for the 30 min chase and 0.13 for the 2 h chase (Fig. 4d). This agrees well with super-resolution fluorescence microscopy analyses^38^. Besides, the original DNA and replicated DNAs were not tightly coinciding with each other due to the offset that is needed to perform a relaxed DNA operation. This is due to the spatial dynamics of replicated DNA that moves out of the condensed chromatin domains to adjacent replication foci during the short chase time but later comes back to these original condensed domains to re-distribute the entire newly replicated DNA^39^.

**Fig. 5:**
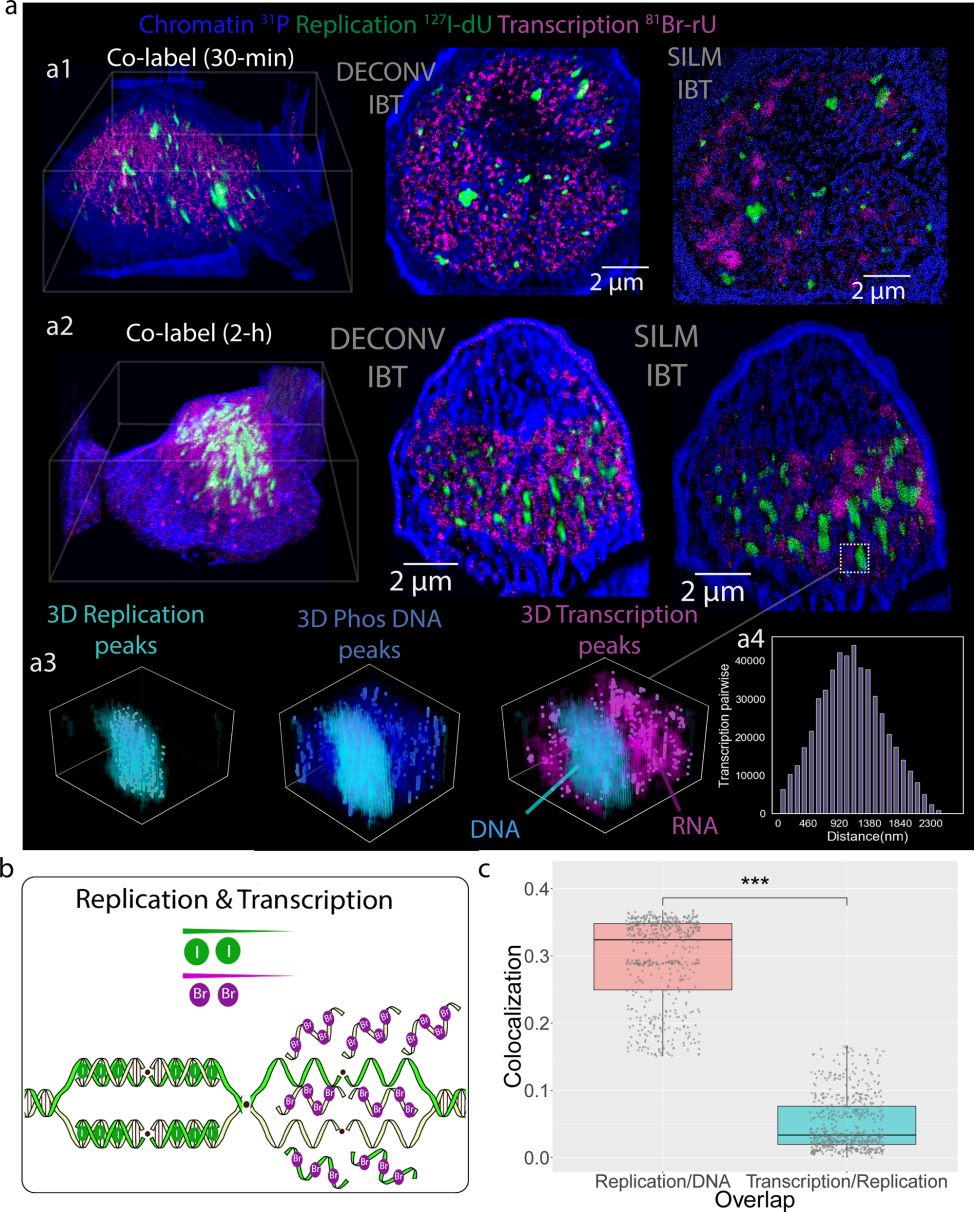
Spatial segregation of replication and transcription visualized by ion beam tomography. **a** Transcript synthesis was detected with ^81^Br-rU (magenta), and DNA replication was detected with ^127^I-dU (green) by simultaneous 30 min and 2 h incubation of Nalm6 cells with labels. The ^31^P channel (blue) reflects primarily nucleic acids. **a1** Nalm6 cell co-labeled to visualize RNA and DNA synthesis for 30 min. The first image shows the 3D render. The middle image illustrates the Deconv-IBT images for RNA transcripts (magenta) and replicating DNA (green) near the chromatin (blue). The right image shows the SILM-IBT results for 100 slices from three channels. **a2** Another Nalm6 cell co-labeled for RNA and DNA synthesis for 2 h. From left to right: 3D render, Deconv-IBT, SILM-IBT results for ^127^I-dU, ^81^Br-rU, and Phosphorous channels. **a3** In the SILM-IBT data, high-density signals in replicated DNA tomograms (3D replication peaks) were localized and identified by a green synthetic circle, and also overlaid by the semi-transparent 3D visual of the original voxel values. Similarly, peaks in the phosphorus and transcription channels were also identified and overlaid with the original voxel distributions. In the 3D transcription plot, the DNA phase and RNA phase were separately obtained, suggesting a clear phase separation approach. **a4** Transcription peaks were separated by distances that exhibited a range from 100 nm to 1.7 µm and averaged around 1.1 µm. **b** As shown schematically, our data indicate that replication and transcription types of machinery are spatially segregated. Iodine (I) was incorporated into the replication forks and bromine (Br) was added to newly synthesized RNAs. **c** Box plot of co-localization of transcription and replication pixels. Each data point represents an ion beam depth section (*n* = 550 slices over a long IBT scan). The difference in the box plots had a significant *p*-value < 2.2e−16 (***) by the Wilcox test (Two-sided). Median, first and third quartile, and 95% confidence interval of the median are shown.

To analyze chromatin states together with DNA and RNA synthesis^40^, ^127^I-dU and ^81^Br-ribouridine (^81^Br-rU) were used to monitor the synthesis of DNA and RNA, respectively, in three dimensions. The mutually exclusive mechanisms of transcription and replication have been widely studied by sequencing but not by multiplex imaging^41–43^. Nalm6 cells synchronized in S-phase or not were incubated for 30 min or 2 h with both ^127^I-dU and ^81^Br-rU, followed by fixation and ion beam tomographic imaging (Fig. 5a). The cell with longer incubation (2 h), visualized in Fig. 5a1, exhibited an enhancement in the transcription signal but preserved the anticorrelated spatial patterns when compared to the shorter incubation (30 min), depicted in Fig. 5a2. In 2 h incubation, 3D peaks were quantitatively analyzed in the replication, transcription, and phosphorus channels near a dominant replication site. 3D visuals of peak positions validated separate RNA and DNA enriched subcellular volumes (Fig. 5a3). Pairwise distances in between peaks in the Br-rU transcription channel showed a large distribution from 100 nm to 1.7 µm separation, yielding an average of 1.1 µm (Fig. 5a4). To quantify the relative spatial distribution observed in simultaneous transcription and replication processes (Fig. 5b), the CC was determined by comparison of pixels in the replication channel (^127^I) and the chromatin (^31^P) channel across 550 slices. For non-synchronized cells, the CC was 0.32 for the replicated DNA and total nucleic acid but was negligible (CC 0.03) for replicated DNA domains as compared to regions that reflect the presence of RNA (Fig. 5c). The simultaneous RNA–DNA measurements in S-phase Nalm6 cells also exhibited the spatial disconnect (Supplementary Figs. 36–38). The future of these IBT experiments is to explore separation biology in metabolic regulation of subcellular volumes^44–46^.

To validate our observations Nalm6 cells were pulsed with ^81^Br-rU for 30 min and treated for 30 min with a transcription inhibitor α-amanitin^47^, and followed by fixation and ion beam imaging. During the incubation with α-amanitin, transcript synthesis was repressed in the decondensed and compact chromatin regions (Supplementary Fig. 39). The 3D renders and zoomed images from signals in the ^31^P and ^127^I-dU channels, both mostly reflective of DNA, validated the spatial segregation of replicated DNAs and newly synthesized RNAs near chromatin. To explore the efficacy of the drug on active transcription, another cell that had been simultaneously treated with α-amanitin and ^81^Br-rU for 2 h was fixed and imaged. Drug treatment significantly reduced transcription by 0.67-fold after cell volume normalization relative to a cell not treated with α-amanitin (Supplementary Figs. 40 and 41). To further validate the IBT approach, ^127^I-labeled rU was used to label transcripts and ^81^Br-dU was used to label replication forks in Nalm6 cells. Even at the level of individual pixels in raw ion images, spatial segregation of replicated DNAs and newly synthesized RNA was apparent in different depth sections (Supplementary Fig. 42).

The IBT method was used to visualize specific transcripts using the single-molecule FISH (smFISH) strategy^48^. Previously, FISH was implemented in the SIMS field for abundant transcripts^49–52^, but not for the detection of single RNA molecules. Instead of using fluorescent tags, the oligonucleotide probes were conjugated to biotin and streptavidin-conjugated 5 nm ^197^Au nanoparticles were used to detect biotin. The nanoparticles allowed the detection of single RNA molecules without the need for amplification. As a proof of concept, 24 biotinylated oligonucleotides of 20 nucleotides in length were designed to hybridize to the highly expressed ActB mRNA (Supplementary Fig. 43a). Use of multiple oligonucleotides that target the single mRNA result in significant signal enhancement relative to a single probe.

First, spinning disk confocal fluorescence microscopy using the Alexa488 conjugated anti-streptavidin antibody was used to validate probe hybridization. *ActB* mRNA molecules were highly expressed in the cytoplasm of a HeLa cell and a few actively transcribing RNAs were also detected in the nucleus (Supplementary Fig. 43b). For IBT experiments, cells were incubated with ^127^I-dU for 23 h, then RNA was labeled by incubation with ^81^Br-rU for 1 h, followed by fixation, permeabilization, and smFISH. Newly synthesized RNAs were measured by analysis of the incorporation of ^81^Br-rU, and *ActB* transcripts were detected by smFISH in the ^197^Au channel. In a HeLa cell, the 25 µm central portion was imaged across 450 depth sections. *ActB* mRNA was observed at depth levels ranging from 50 to 350 slices (Supplementary Fig. 43c). Ion-beam tomographic RNA images were then visualized by 3D rendering (Supplementary Fig. 43d).

### Computational dissection of subcellular organization

The ion beam 3D subcellular organization was modeled by hierarchical clusters, correlation, and association maps. Tomographic information cubes were represented as data frames with spatial coordinates (*x*, *y*, *z*) and ion pixel values. A systematic progression of spatial positions and association maps of replicated DNAs, newly synthesized RNA, phosphorous (DNAs, phosphorylated proteins, and nucleic acids), and total proteins (^34^S as the base of amino acids) were computationally validated in the ion beam tomograms from previously presented data. To explore the unsupervised volumetric distribution of ion beam reconstructions, a spatially resolved 3D clustering approach will be then applied to IBT data cubes under wild type and drug-treated conditions.

First, Euclidian distances between pairs of ion beam voxels from each channel were calculated to determine the relative spatial positioning in two interacting ion sub-volumes for distinct ion channels. Clustering of these Euclidian distances for each pairwise interaction in the ion images provided spatial proximity (Supplementary Fig. 44a) of simultaneous incorporation of ^127^I-dU for replicated DNA and ^81^Br-dU for another replicated DNA. Two large significant clusters were observed with high similarity (dark red) across the diagonal hierarchical maps. Replicated DNAs in both ^127^I-dU and ^81^Br-dU channels were organized at high spatial proximity across subcellular volumetric partitions. Next, to study global organization matrices for voxel values of ^127^I-dU, ^81^Br-dU, and phosphorus (^31^P) were converted to Pearson correlation coefficients and represented as 3 × 3 correlation maps (Supplementary Fig. 44b). A high degree of correlation (dark blue) was obtained for the corresponding replicated DNA channels in ^127^I-dU and ^81^Br-dU, along with a slightly reduced correlation of replicated DNA channels with phosphorous. An association map between the replicated DNAs showed wider circular coverage (corresponding to the higher association) in the Chord diagram owing to the similar spatial distributions of DNA pixels, while replicated DNAs showed reduced association with phosphorus due to the contaminants from additional nucleic acids and phosphorylated proteins in that channel (Supplementary Fig. 44c). Second, the relative spatial distribution of early replicated DNA (^127^I-dU) and late replicated DNA (^81^Br-dU) that are separated by 30 min of cell division was determined from the analysis of Euclidian distances between ion beam voxels from each ion channel. Hierarchical clustering of early replicated DNA and late replicated DNA revealed again two significant clusters with a smaller size (Supplementary Fig. 44d) and lower Pearson coefficient values compared to the synchronous replication data (Supplementary Fig. 44e). In the association maps, early and late replicated DNA was associated the most with the largest circular area, suggesting that the majority of the correlations are between these regions (Supplementary Fig. 44f). Third, relative spatial modeling of early (^127^I-dU) and late (^81^Br-dU) replicated DNAs with 2 h delay were studied. Hierarchical clusters of Euclidian distances exhibited even smaller clusters (Supplementary Fig. 44g) compared to both synchronous and 30 min delay replication data. A correlation map showed significantly lower values for early and late replicated DNAs and smaller values for phosphorus and total proteins (Supplementary Fig. 44h). An association map exhibited a reduced circular area in the diagram (in other words, diminished correlation) between early and late replicated DNAs, together with similar associations of phosphorus and proteins (Supplementary Fig. 44i). The last step of validation was to study the relative spatial positioning of replicated DNA (^127^IdU) and newly synthesized RNA (^81^Br-rU) after 2 h of incorporation. The clustering of Euclidian distances between the voxels of replicated DNAs and synthesized RNAs showed four distinct clusters (Supplementary Fig. 44j). Two of these clusters were diagonal distributions for overlaps and the other two were in the top-left and bottom-right corners of the map. These demonstrated a spatial isolation model of replication and transcription machinery^42,43^. Correlation maps showed minimal correlation values of replicated DNAs and new RNAs, while considerable interrelation of phosphorous and proteins with replication and transcription (Supplementary Fig. 44k). Association maps showed minimal relevance of replicated DNAs and new RNAs (Supplementary Fig. 44l).

**Fig. 6:**
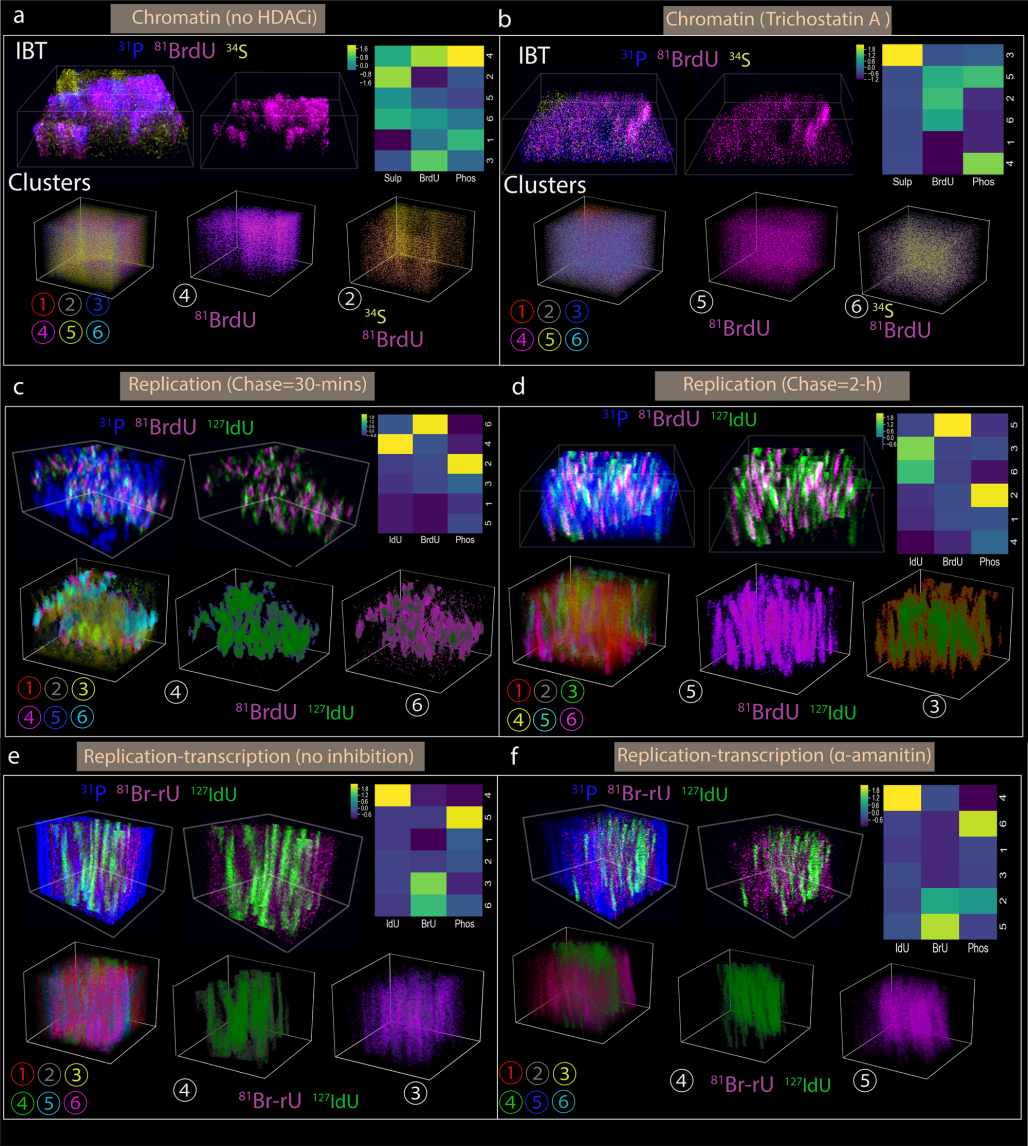
Spatially Resolved 3D Clustering of IBT data informs subcellular structural organization. **a** Chromatin (no HDACi): 3D K-means clustering analysis in a HeLa cell. 3D IBT renders of ^81^Br-dU (chromatin), ^31^P, and ^34^S channels, three-channel merge (left), and only ^81^Br-dU (right). 3D six clusters for an IBT data cube, uniquely colored, and 3D visualized. The pixel values of ^81^Br-dU were displayed on cluster 4 and similarly for ^81^Br-dU and ^34^S on cluster 2. Each ion channel was plotted as a heatmap, colormap blue to yellow. **b** Chromatin (HDACi): 3D clustering of another HeLa cell treated with ^81^Br-dU under TSA drug. 3D IBT renders of TSA treated cells for the merge and single channel. ^81^Br-dU values on cluster 5 and ^81^Br-dU and ^34^S on cluster 6. Z-score IBT maps for Phos (^31^P), ^81^Br-dU, and Sulp (^34^S), colormap blue to yellow. **c** Replication (30 min): 3D representation of a Nalm6 cell incubated with ^127^I-dU and ^81^Br-dU with 30 min chase. 3D IBT visual of ^127^I-dU and ^81^Br-dU (chromatin) with ^31^P channel, three-channel merge (left), and only ^81^Br-dU (right). ^81^Br-dU (magenta) and ^127^I-dU (green) values were overlaid on clusters 4 and 6. Heatmap for ^127^I-dU, ^81^Br-dU, and Phos (^31^P), z-scored ion values. **d** Replication (2 h): 3D analysis of another HeLa cell treated with ^127^I-dU and ^81^Br-dU with 2 h chase. 3D IBT of ^81^Br-dU and ^127^I-dU (chromatin) with ^31^P channel. ^81^Br-dU (magenta) and ^127^I-dU (green) values were shown in clusters 5 and 3. Heatmap for ^127^I-dU, ^81^Br-dU, and Phos (^31^P), z-scored ion signals. **e** Replication-transcription (no inhibition): 3D maps of Nalm6 cell simultaneously treated with ^81^Br-rU (transcription) and ^127^I-dU (replication) for 2 h. 3D IBT representation of ^81^Br-rU (transcription) and ^127^I-dU (replication) along with^31^P channel. ^81^Br-rU was displayed on cluster 3 and ^127^I-dU on cluster 4. Normalized heatmap, colormap blue to yellow. **f** Replication-transcription (with α-amanitin): 3D visualization of another Nalm6 cell simultaneously treated with ^81^Br-rU (transcription) and ^127^I-dU (replication) for 2 h. 3D IBT renders of ^81^Br-rU (transcription) and ^127^I-dU (replication) along with^31^P channel. ^81^Br-rU was overlaid on cluster 5 and ^127^I-dU was on cluster 4. Normalized z-score heatmap, colormap blue to yellow.

3D spatially resolved subcellular features were identified by a K-means clustering algorithm^53,54^ on the volumetric IBT data cubes (Fig. 6). In this 3D clustering method, ion voxels from three channels (number of channels, *M* = 3) were analyzed by a distance metric on the 3D space to cluster six (number of clusters, *C* = 6) unique groups (Supplementary Fig. 45). 3D K-means based classification method then visualized spatially preserved ion signals from each cluster. This 3D volumetric clustering method is broadly applicable to multiplex imaging channels *M* > 3 to identify spatially intersecting regions of 3D datasets in *N* > 6 clusters.

Based on the 3D IBT data, three pairing subcellular data cubes were analyzed by 3D clustering. The first one was to visualize the change in 3D chromatin distribution in response to the HDAC inhibitor drug, TSA. In a HeLa cell, replicated DNA was measured by ^81^Br-dU incorporation for 24 h and IBT measurements included phosphate (^31^P) and sulfur (^34^S) channels (Fig. 3c, left cell in the first row). 3D spatially resolved grouping of the three channels (upper row: IBT renders, bottom row: clusters) provided significantly varying z-score values across six volumetric clusters, providing chromatin patterning that is non-uniformly distributed in the subcellular volumes (Fig. 6a). In the case of another HeLa cell (Fig. 3c, the right cell in the first row) treated with both ^81^Br-dU and TSA drug, these six clusters show reduced variability, suggesting uniformly distributed and smaller DNA pieces (Fig. 6b).

The second one was to quantify intersecting subcellular volumes in two distinct separations between replicated DNAs using 30 min and 2 h chase (Fig. 4b). Nalm6 cells were treated by ^127^I-dU and ^81^Br-dU at two distinct time points (early and late) that are separated by a chase duration in only cell culture media. 3D clustering on ^127^I-dU, ^81^Br-dU, and Phosphate channels yielded two dominant clusters for each replication channel, wherein early replicated DNA exhibited higher z-score volumetric values compared to the late one (Fig. 6c). This difference between early and late replicated DNAs on the heatmap was higher for a longer chase time of 2 h, yielding a smaller number of intersecting voxels in the 3D replication forks (Fig. 6d).

The last one was to compare subcellular volumes of immune cells under perturbation by transcriptional inhibition. Nalm6 cells were simultaneously incubated by ^127^IdU for tagging replicated DNA and ^81^Br-rU for newly synthesized RNA for 2 h (Fig. 5a2). Spatially resolved 3D clustering of replication and transcription channels exhibited entirely isolated 4th and 3rd clusters due to the spatial separation of replicated DNAs and new RNAs (Fig. 6e). In the case of the α-amanitin treated cell, clusters were similar, except that the transcription signal (^81^Br-rU) now showed more uniform distributions across the z-score values of each cluster on the heatmap (Fig. 6f).

Together, repeatability and consistency of association maps for unique cells with systematic metabolic labeling showed a hierarchical and comparative structural organization of replicated DNA, synthesized RNA, total proteins, and natural phosphorus signal. The addition of protein factors, transcriptional regulators, lipids, and natural point of source cell signatures can benefit from the presented computational modeling to decode subcellular patterns both in health and disease.

### IBT-based histology

To demonstrate IBT’s utility in primary tissue, chromatin and its interactions with other molecular distributions were evaluated in formalin-fixed paraffin-embedded (FFPE) 10 µm thick lymph node tissue section from a T cell lymphoblastic lymphoma patient. We detected ^31^P, ^34^S, ^37^Cl, ^14^N, and ^12^C in individual CD3^+^ T cells (identified by standard immunohistochemistry) at sub-100 nm ion beam resolution across 600 depth sections (Supplementary Fig. 46a). The ^31^P signal was located primarily in the nucleus, as expected, and the ^34^S ion signal was enriched in the cytosol. Other channels showed high signals mostly in the cytosol and extracellular regions due to the presence in the tissue of numerous proteins in the extracellular space. A 3D rendering is presented from two different viewing angles in Supplementary Fig. 46b.

Chromatin topology is known to be tightly regulated by interactions with proteins. Super-resolution microscopy has been deployed previously to visualize interactions of chromatin and protein clusters in individual cells from culture^55^. For pairwise detection of chromatin interactions with protein in tissues, a molecular neighborhood analysis was performed on ^31^P (primarily chromatin) and ^34^S (proteins) images. To spatially partition subcellular regions, a 2 × 2 pixel region was segmented by the center-of-mass of the smaller regions and Delaunay triangulation was performed. Distinct spatial subregions showed enrichment of strong DNA-protein interactions in the nucleus; protein-protein contacts were detected in the cytosolic and extracellular regions. The 2D neighborhood maps and corresponding heatmap for the 50^th^ and the 300^th^ depth sections of the IBT images are shown in Supplementary Fig. 46c, d, respectively. 3D Delaunay tetrahedrons of subcellular volumes are shown in a 3D plot in Supplementary Fig. 46e and as a heatmap in Supplementary Fig. 46f. Such label-free neighborhood analyses at high resolution within FFPE samples will enable analyses of intracellular changes that occur upon cellular recognition events.

Multiplex histology has been limited to 2D spatial analysis^5^. Volumetric analysis of tissue biopsy samples using IBT will enhance our understanding of cell-to-cell interactions in the context of highly localized details of receptor and marker distributions^56^. As a proof of principle for use of IBT in histology, we analyzed the interactions of immune cells (CD45^+^, label: ^169^Tm) and cancer cancers (CK19^+^, label: ^141^Pr) within a tumor sample from a patient with cholangiocellular carcinoma under oxygen bombardment (See Supplementary Methods). A non-uniform 3D distribution of surface markers was observed (Supplementary Fig. 47). Polarization of surface markers helps determine the potential capacity of a cell from the ion beam snapshots. These 3D histology details show the promise of IBT for quantifying cell-to-cell interactions.

## Discussion

IBT is a mathematical and technical framework for the analysis of three-dimensional MIBI. The method enables analysis of ion beam images acquired at multiple depths. In a typical IBT experiment, cells were initially imaged at 0.2-4 pA ion current over 1000 axial scans. Previously, these 3D image stacks were just processed by a Z-correction method^57,58^ that only flipped etching patterns of ion signal from the top to the bottom of the cell. However, it is not been applied to digital enhancements of image stacks using deblurring mathematics, localization of ion signal, or artificial intelligence solutions. In the presented IBT platform, after the binning of images, digital deconvolution was applied to the transformed ion image series using a hybrid deblurring algorithm and an ion beam current-dependent point-spread function. SILM and Deep-learning framework allowed sub-ion-beam-width reconstruction for whole cells. Three-dimensional processing was implemented by spatial clustering and molecular neighborhoods. In cultured cells and tissues, IBT revealed three-dimensional volumetric distributions of genomic regions, RNA transcripts, and protein factors with sub-25 nm lateral and 5 nm axial resolution.

Of note, 3D super-resolution imaging by fluorescence microscopy also relies on the data from a single cell from extensive image arrays. For instance, 1 section requires 10,000 and more frames, and combining 100 sections from a single cell requires a 24 h experiment for nuclear imaging in a single cell by STORM/PALM or even STED, together with 3-plex molecular detection. The current IBT scheme presents results from single cells at the 20 h/cell acquisition rate. Thus, the throughput of 3D STORM and SILM experiments are on the same orders and comparable. The IBT presents a universal platform for 3D subcellular biology at a matching throughput.

Another important aspect of the IBT is that the sub-beam-width precision was obtained but not the sub-ion-beam-diffraction limit. While the ion beam field indicated the existence of a diffraction limit for ions, it is in the order of picometer nanometers and much smaller than the ion-beam-width of 50–100 nm. Thus, diffraction is negligible when considering the resolution limits for an ion beam imaging system. In the context of IBT precision enhancements, the spatial details are in the order of nanometers that are larger than the diffraction limit of ions. Thus, there is still a considerable gap to reach true super-resolution that can break the diffraction limit of ions beyond the IBT advancements.

The presented IBT platform will open new avenues of subcellular imaging research to study aberrant cells:

- Direct visualization of 3D chromatin at sub-25 nm spatial details impacts the study of the role of the nucleus in the determination of cell function for dissecting epigenetic mechanisms.
- Subcellular 3D metabolic profiling for replication dynamics will find significant use in cell cycle regulation as a critical regular of therapeutic response. While not presented here, a systematic study of replication stress under various drug treatments in cancer models (human/mice) will shed light on subcellular dynamics in cultures and tissues.
- The nascent transcriptome plays a key role in cell function in cancers. Thus, the spatial visualization of the relative DNA-RNA synthesis process will provide a subcellular signature in therapeutic effects.

When additional time points are evaluated and the strategy is combined with pseudotime techniques^1,59^, it should be possible to describe trajectories of changes in biological features at the single-cell level. The ion beam tomographic analysis pipeline described here can be used in the analysis of data obtained using mass spectroscopy-based imaging technologies^60,61^. In the future, 3D machine learning algorithms^62^ could be used to train and classify MIBI images of subcellular regions for cell-specific analyses. Deep-learning-based IBT technology will eventually be used for the creation of an epigenetic atlas of cells^63^ that will be useful in both basic research studies and the clinic.

## Methods

### Synthetic ion beam image construction

Ion-beam images were modeled based on linear image formation theory that is contaminated by a multiplicative noise and modified by the ion extraction efficiency:

1 $$I\left( {x,y,z} \right) = \frac{{P_{{\rm{out}}}}}{{P_{{\rm{in}}}}}\left[ {O\left( {x,y,z} \right) \otimes {\rm{PSF}}\left( {x,y,z} \right)} \right]x\,N\left( {x,y,z} \right)$$

where *I*(*x,y,z*) is the detected image at the detector output, *O*(*x*,*y,z*) is the true object distribution that includes proteomic samples (individual proteins and cellular labels), PSF(*x,y,z*) is the point-spread function of the ion beam imaging platform for each gun source and elemental labeling, *N*(*x,y,z*) is the multiplicative noise that is calculated from an ion image of interest, *P*_in_ is the density of atoms impinging on the sample per unit time, and *P*_out_ is secondary ions extracted from the sample per unit time.

Each true object, *O*(*x*,*y,z*), is an array of points distributed in space adjusted based on the required synthetic pattern. Initially, we assumed an array of proteomic signatures that were separated by a distance (*d*). The simulated true distribution was then convolved by PSF(*x,y,z*) of the ion beam imaging system. Typically, the *x/y* extent of the PSF is limited to ion beam width (50–500 nm) and the *z* extent is related to the sample etch rate (1–30 nm). Using *x/y*:100, *z*:5 nm, we obtained a blurry image without noise. The convolved image was then contaminated by the additive and multiplicative noise factors from electronics and other factors. As background level is very low in ion beam imaging, we only considered multiplicative noise, *N*(*x,y,z*), as a gamma distribution that was computed from a sample ion image. During the image acquisition, ions are lost during the secondary ion beam conversion and electronically readout. We included an additional multiplicative factor for ion extraction efficiency,*P*_out_/*P*_in_, in the range of approximately 3%. Finally, the synthetic ion beam image, *I*(*x,y,z*), was digitally sampled by a pixel size of *x*/*y* to be in the 50–100 nm range.

### Ion-beam imaging experimental design

Oxygen and cesium were used as the sources for NanoSIMS 50L (Cameca). The ion beam current determines resolution and sensitivity. The lower current provided higher spatial resolution, whereas the higher current in the cesium beam allowed deeper etching for faster 3D imaging (Supplementary Fig. 25). The lateral image resolution is governed by the width of the ion beam size for each source (Cs, O^−^/Au). The pixel size should be smaller than half of the highest spatial frequency of the ion beam image based on the Nyquist criterion. Thus, the pixel size should ideally be smaller than a large portion of the beam width:

2 $${\Delta}x,{\Delta}y \le {\Delta}w_{{\rm{beam}}},\;{\mathrm{where}}\,{\Delta}x = \frac{{A_{{\rm{scan}}},x}}{{N_x}}{\mathrm{and}}\,{\Delta}y = \frac{{A_{{\rm{scan}}},y}}{{N_y}}$$

where Δ*x*, Δ*y* are pixel sizes, Δ*w*_beam_ is ion beam width, and *N*_*x*_, *N*_*y*_ are the total number of pixels, and *A*_scan_, *x*, *A*_scan_, *y* are the total ion image area. For instance, *N*_*x*_ *or N*_*y*_ = 256 × 256 for a cellular area of *A*_scan *x y*_ = 20 µm × 20 µm yields Δ*x*, Δ*y* = 78 nm, a pixel size that performs well for Δ*w*_beam_ = 100 nm. Resolution and pixel size comparisons were performed for the same cell (Supplementary Fig. 21). Much smaller pixel sizes are not optimal as the image acquisition time significantly increases. Dwell time per pixel must also be tuned. The typical value for the NanoSIMS is 1000 ms. Better sensitivity was achieved around 2000 ms at the cost of increasing acquisition time per pixel; however, ion beam images were distorted at dwell times longer than 2000 ms.

Ion-beam imaging requires conductive sample carriers, and thus, bare glass is not suitable. Custom-sized (7 mm × 7 mm) and (18 mm × 18 mm) silicon wafers (Silicon Valley Microelectronics) were used to host cells and tissues. Gold-coated substrates were not appropriate for long depth scans as the regions without cell materials were etched and caused charge buildup, which significantly distorted images.

Samples were loaded into a NanoSIMS 50L (Cameca) device, and a vacuum was set within the first 1–2 h. To adjust the mass selection, 3 µL master mix buffer (^127^I-dU and ^81^Br-dU stock solutions) was dried on a large spot next to the cells and was imaged before cell or tissue imaging. After etching an elementally enriched region by high current ion beam sputtering, SIMS signal levels became detectable. Exact mass values were determined and set from the tuning window. Optics and detector settings were fine-tuned to maximize the ion beam signal. Using optimized mass settings, cells were imaged at first high sputter rate (aperture setting D1_0 with >100 pA current) to remove outermost layers, followed by low current ion beam imaging (aperture setting D1_3 with >0.2–4 pA current) for high-resolution analysis. In this work, entrance slit and aperture slit values were kept at 0 for maximized ion signal. In the SIMS panel, elements need to be separated by more than 3 atomic mass units. Based on the optimum parameters, a large ion beam scan was captured over a 50 µm × 50 µm area with a 256 × 256 pixel window. A dwell time of 1000 ms per pixel was used. Once a region of interest (ROI) was defined, a 20 µm × 20 µm subcellular area was scanned over 1000 depths, typically taking 18 h of image acquisition per cell. Simultaneously data on seven ion channels (^12^C, ^12^C^14^N, ^19^F, ^34^S, ^31^P, ^81^Br, and ^127^I) and a secondary electron microscopy image were captured at each scan. The acquired images were visualized by an open-source OpenMIMS plugin in Fiji and ImageJ. Batch analysis and 3D analysis are done by the presented analysis pipeline.

### Mathematical pipeline for ion beam tomography

Raw ion beam images were saved as.im format files, and a MATLAB function was used to convert.im files to TIFF files. Image alignment is required for different depth scans as the ion beam drifts during the acquisition. To address this problem, a cellular subregion that stayed almost stationary across all the depths was selected, and depth images were spatially registered down to pixel levels. Additional fiducial markers were included to perform the registration. One approach was to coat microparticles such as iron (^55^Fe) microbeads (2–6 µm diameter) onto the cell sample and visualize the bead along with the subcellular ROI (e.g., entire nucleus) in the electron microscopy channel or specific elemental image (Supplementary Fig. 23). Registrations across ion imaging stacks were done in Fiji and MATLAB.

Raw ion beam images were summed over a few depth image sections (1 to *n*, where *n* varies between 3 and 20 slices) to improve the signal-to-noise ratio (SNR) of the combined ion beam images:

3 $${\rm{SNR}}\left( {{\rm{dB}}} \right) = 20\log _{10}\left( {\frac{{\mu _{{\rm{signal}}}}}{{\sqrt {\frac{{\mathop {\sum }\nolimits_{i = 1}^n \left( {B_i - \mu _{{\rm{background}}}} \right)}}{N}} }}} \right)$$

where *μ*_signal_ is the mean signal value and *μ*_background_ is the mean background value, $\mathop {\sum}\nolimits_{i = 1}^n {\left( {B_i} \right)}$ is the background pixel value, and *N* is the number of pixels in the background region of the ion beam cellular image.

Summing up to 10 subsequent slices increased SNR exponentially (up to SNR=7 dB), whereas SNR slightly increased after binning more than 10 slices (up to SNR=11 dB). Thus, subgrouping a large depth scan into *n*=10 slice groups provided optimum mathematical IBT performance. Based on the quality of raw image scans, the *n* was increased for dim ion signal and was decreased for bright ion images. To avoid axial resolution loss, we binned 10 slices and incremented by one slice at a time over the entire section. Thus, IBT preserves original depth resolving power down to 5–10 nm axial resolution. Multi-slice summations were performed in Fiji, Python, or MATLAB modules.

A 3D image enhancement algorithm was applied to the summed images for digital confocality on ion beam imaging. The Lucy-Richardson (LR) deconvolution algorithm was used to deblur the image iteratively based on the modification of the PSF at each iteration:

4 $$O(x,y,z)^{n + 1} = O(x,y,z)^n\left( {\frac{{I(x,y,z)}}{{O(x,y,z)^n \otimes {\rm{PSF}}\left( {x,y,z} \right)}}\widehat { \otimes {\rm{PSF}}\left( {x,y,z} \right)}} \right)$$

where *O*(*x,y,z*)^*n*^ and *O*(*x,y,z*)^*n*+1^ are the object distributions in *n*th and *n+1*th iterations, *I*(*x,y,z*) is the original ion beam image, ⊗ is the convolution operation, PSF(*x,y,z*) is the original PSF, and $\widehat {{\rm{PSF}}\left( {x,y,z} \right)}$ is the modified PSF. The algorithm outputs *O*(*x,y,z*)^*n*+1^ ion image after *n+1* iterations. After setting the image standards for each channel and element, the iteration number was fixed between 3 and 10 to provide sharper ion beam images.

Here, we performed a hybrid deconvolution, a combination of iterative LR algorithm, and a blind PSF. Based on the measured current levels of the ion beam imaging (0.2 to 4 pA), a PSF width was computed and included in the LR deconvolution algorithm. The deconvolved images were then lightly (2 × 2) filtered by a Gaussian function or median filter to reduce the image noise. The resultant images were passed to the 3D quantification and representation. Digital deconvolutions were performed by custom-written MATLAB (2019a) and Python algorithms.

Raw IBT data cubes with a series of ion images were first three-times (3×) interpolated in Fiji using the average of nearby pixels and bilinear settings, creating smaller pixel sizes and better localization. These interpolated images were then included in a folder, from which a subset of images (20–100 slices) was selected and localizations were performed in Python on a Jupyter notebook interface. Previously developed STORM package (Zhuang Lab, Harvard) was used to define initial settings, but the threshold values were adjusted based on the signal-to-noise ratio of raw images. The localized points were then convolved by a Gaussian using a tunable sigma value to make a SILM image. The final reconstructed SILM images were then saved in a folder for post-analysis.

An accurate sampling of SIMS datasets is needed for SILM analysis. Typically, a single cell should be scanned with 256 × 256 or 512 × 512 scans over an area of more than 10 microns, providing a sub-50 nm pixel size for a sub-100 nm PSF width of ion beam imaging. The resolution limit in an ion beam imaging system is PSF width—so, a 100 nm beam size can resolve features down to 100 nm. Then, based on the Nyquist ratio, the pixel size ideally should be half of the beam size/minimum feature size, which provides around 50 nm pixel size. Since also the pixel size and ion beam current is low, each pixel would randomly exhibit dark/non-zero values during 3D scans. For sub-50 nm pixel size, we would capture more than 100 I- signals that arise from ~150 base pairs (each bp is 0.34 nm). However, the secondary signal ion generation efficiency is in the order of %5 or less—so in this pixel, we would capture less than 10 counts. Thus, localizing the center of such a pixel and neighboring pixels would localize the existence of high-concentrated DNA regions from a DNA duplex.

In terms of cluster imaging, 3D scans only capture the sub-group of ion signals in the cluster due to the low secondary ion conversion efficiency at each scan. This conversion efficiency is also randomly distributed at the sequential scans. SILM localizes these cluster pieces at high precision, each of which is fitted by a Gaussian curve. We then combine localizations from e.g., 20–100 slices to resolve the size of the cluster. For instance, a 100 nm sized nanotag can even be considered as a cluster because each scan only shows a piece of circular nanotag shape, but not the full circular shape. When we localize signals from 20 slices and sum these multiple Gaussians, we obtain the brightest pixel in the middle of the reconstructed image. In other words, the summation signal in SILM reconstructions is a combination of multiple localized peaks in the subset of clustered isotopes embedded in nanotags. The interpretation of SILM-IBT data is the reconstruction signal is correlated with neighboring pixel localization that can show clusters as well as isolated ion signals. Higher SILM-IBT signals suggest more clustering while the baseline SILM-IBT signature is individual ion counts.

The minimum number of ion signals should be slightly higher than the threshold of background value. Simply, the random pixel noise should not be localized, but less than 10 counts work fine in SILM-IBT reconstructions. For instance, in Fig. 2, original pixel values in sulfur and bromine channels are less than 10 counts, providing SILM-IBT outputs that agree well with Deconvolution-IBT results in terms of high concentration of isotopes across the subcellular volumes. When it comes to higher pixel counts, the localization accuracy increases, but more z-slices will be needed to capture a larger dynamic range. For example, in Fig. 2, the phosphorus channel is brighter compared to other ion channels, which mostly localizes higher count values with the same number of z-slices used in sulfur channel and bromine channel based SILM-IBT reconstructions. Of note, single ion-molecule detection is relatively difficult to claim due to the varying conversion efficiency to the secondary ions. Instead, localization of ion molecules exhibits consistent high-density and low-density ion enrichments from isotopes, as noted in results in Fig. 2. Since Deconvolution-IBT exhibits uniform SIMS structures, these results were validated by comparing SILM-IBT results to the enriched regions in the Deconvolution-IBT reconstructions.

Raw IBT images (Low-resolution) and SILM-IBT images (High-precision) were used to generate a highly precise (sub-ion-beam-width) image. Raw images were trained in comparison to SILM-IBT ground truth images using the CSBDEEP package in the Python interface. Tensorflow and Keras packages were used to generate a model from this high to low-resolution experimental ion images. Epoch number was 100 for 20 iterations. Large images were split into 32 × 32 or 16 × 16 smaller frames to generate hundreds of training data. Using similar conditions, newly acquired low-resolution ion stacks were then processed to predict a high-precision reconstructed image. Deep-learning-IBT reconstructed images reach similar resolution levels of SILM-IBT images without the need for parameter tuning at even low-density biological structures.

Deconvolution, SILM, and Deep-Learning IBT results were processed to explore the 3D distribution of subcellular regions in individual cells. Here we used the *Imaris* module of Bitplane and 3D visualization 1 online interface of Volocity (Perkin Elmer) to handle IBT datasets. Using this 3D rendering pipeline, 3D movies were created of transcription, replication, and chromatin experiments in individual mammalian cells (Supplementary movies 1–14). Next, images were segmented in 3D and connected objects were labeled. For quantitative comparisons of specific subcellular distributions, centroids and volumes of each sub-object were listed as an output excel text file or MATLAB variable, followed by statistical analysis in R studio.

### Analysis

Two separate ion channels exhibited overlaps or spatial segregation for transcription and replication studies. Co-localization of ion channels at each depth was quantified based on the 2D correlation coefficient (CC):

5 $$r = \frac{{\mathop {\sum }\nolimits_m \mathop {\sum }\nolimits_n \left( {P_{mn} - \bar P} \right)\left( {R_{mn} - \bar R} \right)}}{{\sqrt {\left( {\mathop {\sum }\nolimits_m \mathop {\sum }\nolimits_n \left( {P_{mn} - \bar P} \right)^2} \right)\left( {\mathop {\sum }\nolimits_m \mathop {\sum }\nolimits_n \left( {R_{mn} - \bar R} \right)^2} \right)} }}$$

where *P*_*mn*_ and *R*_*mn*_ are two overlapping images, $\overline P$ and $\bar R$ are the mean values of total pixels in each image, and *r* is the CC. Higher *r* values suggested more overlap across all the pixels. Linear fit analysis for the entire 600–1000 ion image slices generated a *r* distribution over a 3D cell, which was then represented as box plots with scatter points. Image co-localization was computed in MATLAB and the coefficient values were exported in text format, followed by R software analysis for box plotting.

Spatial resolution was quantified based on the line scans using the signal drop from 88 to 12% of the maximum to determine image resolution. Using Deconvolution-IBT, The 88–12% criterion provided 55 nm resolution for images acquired with the cesium source, whereas 395 nm resolution was achieved for experiments with the oxygen source. The axial resolution was high as each layer was etched only a few nanometers after interacting with the ion beam. Acquiring 1000 depths across more than half of the B cell showed less than 5–10 nm axial resolution as the cell size is around 10 µm. The 88-12% criterion on the line scan across the depth profile also provided 5 nm axial resolution. Edge analyses were performed in MATLAB.

Elements exhibit distinct sensitivities in ion beam tomographic analysis (Supplementary Fig. 26). High to the low sensitivity of elements imaged by a cesium beam are S, Cl, Se, Br, I, F, Te, Au, Pt, O, C, Si, Ir, Ge, P, Ag, Sn, Os, and Sb. High to the low sensitivity of elements imaged by an oxygen beam are Cs, Na, K, Li, Rb, Ca, Sc, Sr, Ga, In, Ba, Nd, Eu, Y, Pr, Al, Dy, Tb, Ho, Yb, Ce, Sm, Er, Mg, La, Gd, Tm, Lu, Ti, V, Zr, Tl, U, Cr, Nb, Mn, Mo, Th, Hf, Fe, Rh, Sn, Cu, Be, Ru, Ni, Si, Ag, Co, Ta, B, Pb, W, Pd, and Bi. The ratio of sensitivity levels was corrected for accurate mapping of true molecular concentrations in ion beam imaging experiments. These sensitivity comparisons were performed on an Excel sheet.

The Delaunay triangles and tetrahedrons were extracted from IBT images after calculating the center of masses in 2 × 2 pixels, 4 × 4 pixels, or 8 × 8 pixels subregions. The total ion image signal per area and centroids of triangles/tetrahedrons were extracted from MATLAB analysis. Heat maps were then prepared in the R studio with *ggplot2* and *heatmap.2* packages using Python. Ion signal levels of each corner and spatial positions were clustered together to spatially visualize subregions of pairwise molecular interactions in chromatin.

Chromatin states were determined based on the grouping of pixel intensity values in the ion beam data. Fuzzy C-means (FCM) clustering was used to partition the volumetric ion beam data. The classified groups of clustered pixel values were then used to re-color the segmented cells in Supplementary Figs. 28–30. Adjusting cluster dimension (C) allowed the segmentation of fine chromatin sub-states. Tomographic ion images benefited from the rapid identification of distinct density levels in the subcellular regions. K-means and other clustering methods can potentially provide similar segmentation results in subcellular tomographic images. FCM analysis was performed in MATLAB.

The structural similarity index (SSIM) is a quality metric (between 0 to 1) used to determine visual similarities of one image to another based on luminance (I: Intensity), contrast (C: Max/Min pixel intensity difference), and structure (S: features) of an image. SSIM was calculated as follows:

6 $${\rm{SSIM}} = \frac{{\left( {2\mu _x\mu _y + C_1} \right)\left( {2\sigma _{xy} + C_2} \right)}}{{\left( {\mu _x^2 + \mu _y^2C_1} \right)\left( {\sigma _x^2 + \sigma _y^2 + C_2} \right)}}$$

where *μ*_*x*_,*μ*_*y*_ are local means, *σ*_*x*_, *σ*_*y*_ are standard deviations, and *σ*_*xy*_ is cross-variance of the images.

In the MATLAB *ssim (A, ref)* function, the image of interest from the ^127^I-dU channel was compared to a reference from the ^81^Br-dU channel to determine the similarity of spatial chromatin variations. To circumvent dynamic range issues in the raw and reconstructed DNA images, ion images were adjusted to [0, 1] range. These normalized images were then subjected to the SSIM quantification using Python programs.

The spatial algorithm used on the BrdU and BrdU-TSA images was applied to 4 cells from each group that contained voxels in the SILM-IBT reconstructions of chromatin. The statistical significance was computed on the histograms in python. Each chromatin data was divided into 20 by 20 pixel blocks at the outset of the algorithm, and the photutils package in Python was utilized for the detection of local peaks on each of the blocks across the image. The local peaks were detected in the stack by individual thresholding each block in the image. Once the peaks were computed for the images in the BrdU and BrdU-TSA groups, it was averaged across four cells in each group. A step plot was then computed for the averaged peaks along with the underlying probability density function of the images. The BrdU-TSA group showed less number of peaks per 20 by 20 pixel area compared to the BrdU only group. These chromatin analyses were done in Python.

To explore relative spatial positioning across newly synthesized DNA and RNA, pairwise distances were converted to Euclidian distances ($d_{{\rm{Euclidian}}}(x,y) = \sqrt {\mathop {\sum}\nolimits_{i = 1}^n {\left( {x_i - y_i} \right)^2} }$) and the resultant distance matrix was clustered to determine the subcellular organization (Supplementary Fig. 44a, d, g, and j). To calculate global correlation patterns across the entire cell volume, a 3D ion tomographic information cube was organized as a matrix array (X, Y, Z, ion voxel value), followed by calculation of Pearson correlation coefficients among replication and transcription data (Supplementary Fig. 44b, e, h, and k). To visualize the association of each subcellular component, chord diagrams were created from the correlation matrix from the previous step (Supplementary Fig. 44c, f, i, and l). These maps were created in R-studio programs.

Microtubules labeled by Alexa Fluor 647 were imaged in STORM-buffers to allow blinking over 3000 frames, followed by fluorophore localizations. While all the frames were added to visualize microtubules at low-resolution, the STORM analysis provided super-resolved microtubule distributions. A similar analysis was done for ^127^I-dU labeled DNA structures in a HeLa cell and captured by NanoSIMS for 50 frames. A simple addition of frames showed a blurry image, but after SILM localizations of isolated ion molecules, finer localized DNA features were obtained (Supplementary Movie 15).

Peak identification was implemented in Python for distinguishing maximum voxels in the processed SILM-IBT images. The SILM-IBT images are comprised of 3 channels. The structure of the original data involved 2D images that are stacked upon each other to form a 3D stack of images, each of which was identified by a corresponding z-slice number. A region of interest was identified manually containing a cluster of voxels. The algorithm then identified blobs or bright spots likely to be cluster centers in the region of interest, wherein the maxima regions in the 3D stack of images were detected using a python package called *photutils*. Each of the 2D images in the stack was applied a threshold individually, and hence the local peaks in each z-slice could be computed. To assign voxels to a potential maximum, the standard deviations of each pixel was calculated during the process of thresholding. Multiple peaks with similar intensities at a given z-slice were recorded by corresponding coordinates. The peaks were represented as a circle and overlaid onto the 3D scatterplot of the original ion channels.

Unsupervised algorithms work efficiently without any labels from the data. Among others, K-means algorithm is computationally adaptable to larger datasets independent of the cluster shapes. K-means method was adapted from other fields including image processing, pattern recognition, and neural networks. Although K-means is an unsupervised clustering algorithm, it is still necessary to specify the number of clusters expected in the dataset. This is often a trial and error process and largely depends on the inherent properties of the dataset.

The K-means method is a special case of Naive Bayes based on the Expectation-Maximization. At the outset, K-means randomly initialized the first iteration of centroids corresponding to the initial set of clusters. A distance metric was utilized to assign each data point to the nearest centroid based on the metric defined. Throughout each iteration, the centroids of the clusters were recomputed based on the points of the newly assigned cluster (Supplementary Fig. 45a). The cycle continued until there was a negligible difference between the centroids computed in successive iterations.

The data was scaled and normalized before the clustering by the K-means algorithm. The purpose of scaling the data was to reduce the variance to a unit value and to subtract the mean, to ultimately change the range of the data across all variables. Normalization of the data was used to adjust the values of the underlying probability distribution, yielding a scale of 0 to 1 for the data variables. In general, normalization enables easier implementation and reduces the memory required to store the variables.

Python 3.7 was the platform used for implementing the algorithm. In the algorithm, the area and the z-range were defined at the beginning for data to be processed. After defining the volumetric region of the cell for further analysis, various mathematical operations were performed on the clustered K-means data to ensure that the data was in the optimal form required for plotting.

A subset of data for visualization can be defined as a collection of elements randomly arranged, which in this case are the clustered data points. Ordered sets are sets that have an index associated with each element. If there exist two sets *X* and *Y*, the following operations can be defined (Supplementary Fig. 45b). The union of *X* and *Y* is the set of elements contained in *X*, or *Y*, or both. The intersection of *X* and *Y* is the set of elements contained in *X* and *Y*. Set difference of *X* and *Y* is the set of all the elements contained in *X* but not contained in *Y*. Python has defined data structures as arrays, a collection of elements stored in memory locations that are contiguous and can be accessed by indexing. In some cases, the data type of the data variable is converted into an ordered set data type, instead of an array data type, as it helps with the intersection, union, and differences operation of clustered data through set operations.

While plotting heat maps of clustered data, normalization and log scaling is crucial to implement. For instance, certain channels such as phosphorous tend to be predominant, and therefore has a higher pixel mean value, in comparison to other channels such as iodine-based channels (^127^IdU or ^127^I-rU). Implementing 3-Dimensional (3D) scatterplots involved adjusting parameters related to color, size, and transparency of the markers used to represent the pixels. This is important to keep the underlying structure of the clustered data, which was not apparent due to the number of samples plotted. Custom colormaps were also defined for the selection of colors for the heat maps, cluster maps, and scatterplots.

### Nanotag fabrication

Fluor (^19^F)-doped silica (^28^Si) nanoparticles (nanotags) were synthesized via a modified Stöber reaction. In brief, 1.7 μl dimethylsulfoxide (DMSO) or 1.7 μl (3-trifluoropropyl) silane (0.1 M in DMSO) were added to a reaction mixture containing 1.45 ml isopropanol, 100 μl water (18.2 MΩ.cm resistivity at 25 °C), 45 μl 28% (v/v) ammonium hydroxide, 65 μl tetraethyl orthosilicate (99.999% trace metal basis), and 3.5 μl (3-mercaptopropyl)trimethoxysilane (MPTMS) to yield 100 nm silica nanoparticles or 100 nm ^19^F-doped silica nanoparticles, respectively, after shaking for 20 min at 1000 RPM. The nanoparticles were collected via centrifugation (5 min at 10,000*g*), washed with ethanol, and stored in ethanol. The nanoparticle size distribution and concentration were measured on a nanoparticle tracking analyzer (NTA; NS-300, Malvern Panalytical, Westborough, MA). All chemicals were obtained from Sigma Aldrich and used without any further purification.

### Cell culture

HeLa cells (Sigma-Aldrich, 93021013) were cultured in Dulbecco’s Modified Eagle’s medium (DMEM 1×; Life Technologies) with 10% fetal bovine serum (FBS, Omega Scientific) and penicillin-streptomycin (Life Technologies) at 37 °C, 5% CO_2_ and passaged every few days with TrypLE Express (Thermo Fisher). Before imaging, cells were cultured on 18 mm × 18 mm or 7 mm × 7 mm silicon substrates overnight. For metabolic experiments, cells were incubated with the isotope of interest for the time indicated. After 24 h, cells on silicon substrates were washed with phosphate-buffered saline (PBS), followed by fixation with 1.6% paraformaldehyde (PFA; Electron Microscopy Sciences) for 10 min. Cells were then washed with PBS and deionized (DI) water a few times and then treated with ethanol. Samples were then dried in a vacuum desiccator for a few days before loading into the SIMS device for experiments. Jurkat clone E6-1 (ATCC® TIB-152™) cells were grown in RPMI 1640 (Life Technologies), 10% FBS (Omega Scientific), and 2 mM L-glutamine (Life Technologies). NALM6 clone G5 (ATCC, CRL-3273) cells were grown in RPMI 1640 (Life Technologies) and 10% FBS (Omega Scientific). Non-adherent Jurkat and Nalm6 cells were coated onto poly-L-lysine (Sigma-Aldrich) modified silicon substrates by spin coating at 30 RCF for 5 min, followed by fixation with 1.6% PFA for 10 min. Cells were either permeabilized for antibody staining or directly washed with PBS and DI water for SIMS experiments.

### Pulse-chase metabolic experiments

HeLa cells on silicon substrates were cultured overnight. For chromatin tracing, cells were incubated in media with 10 μM ^127^I-dU (Sigma-Aldrich) and ^81^Br-dU (BioLegend) for 24 h. For replication studies, HeLa cells were pulsed for 30 min with 20 μM ^127^I-dU. After washing the cells twice with warm PBS, cells were incubated for either 30 min or 2 h in medium without a label. Cells were then incubated for 30 min with 20 μM ^81^Br-dU. Jurkat and Nalm6 cells were pulsed and chased in tubes, followed by centrifugation at 125 RCF for 3 min to exchange buffers. Cells were then coated onto poly-L-lysine-modified silicon substrates and prepared for SIMS as above. For transcription studies, cells were pulsed with 2 mM ^81^Br-rU (Sigma-Aldrich) or 2 mM ^127^I-labeled rU (Sigma-Aldrich) for 30–60 min, followed by washes with PBS and DI water, and PFA fixation. To inhibit transcription, cells were treated with 10 µg/mL α-amanitin (Abcam) for 30 min on ice, followed by washes and PFA fixation. To synchronize the cells in the S phase, cells were treated with 5 µg/mL aphidicolin (Sigma-Aldrich) for 16 h, followed by two washes with warm PBS.

### RNA detection

Twenty-four DNA probes (20-mers of unique sequence) were designed against different regions of *ActB* mRNA in Stellaris Probe Designer (LGC Biosearch Technologies). Each oligonucleotide was biotinylated at the 5′ end (Supplementary Table 1) during synthesis (Integrated DNA Technologies), and all were pooled. Endogenous biotin and avidin binding sites in cells were blocked to avoid non-specific binding (Abcam, cat #ab64212). Cells were incubated with 2 nM oligonucleotide mixture in a buffer containing 2 × SSC, 10% dextran sulfate, and 30% formamide at room temperature. After overnight incubation, the cell sample was washed with 30% formamide for 20 min and twice with 2 × SSC for 5 min. Samples were then incubated with streptavidin-conjugated 5 nm gold nanoparticles (Cytodiagnostics, cat#AC-5-04-05) in a PBS1× buffer for 1 h at room temperature. After washing the cells with DI water, the sample was dehydrated and imaged by IBT.

### Confocal microscopy

Cells were prepared on coverslips (22 mm × 22 mm) for fluorescence experiments. Labeling and culturing conditions were the same as for IBT protocols. A spinning disk microscope (Andor DragonFly) was used to capture control fluorescence images of cells. A 60× objective lens with 1.6× tube magnification was used, providing a total 96× magnification. Alexa Fluor 488 Streptavidin Conjugate (Thermo Fisher, cat #S32354) was used at 1:100-1:500 dilutions to target biotinylated oligonucleotides that are specific to *ActB* mRNA for control experiments in HeLa cells. The power level was adjusted to 50% of the 488 nm laser. A micromanager interface was used to capture images. ImageJ was used to merge images for pseudo coloring.

### Statistics and reproducibility

Images exhibited in Figs. 1–4 and Supplementary Figs. 3, 4, 5, 13, 16, 28, 29, 30, 31, 32, 33, 36, 37, 38, 39, 40, 42, 43, 46, and 47 denote data from at least two independent experiments. Statistical analyses were performed with R software (Version 1.1.423, 2009–2018 RStudio, Inc). MATLAB analysis was done in the 2019a version. Python processing was performed in the Anaconda environment using Jupyter notebook (Version 3.7.4). Box plots show median, first and third quartile, and 95% confidence interval of the median. The two-sided Wilcox test was used for data pair comparisons. A p-value of <0.05 was considered statistically significant.

### Reporting summary

Further information on research design is available in the Nature Research Reporting Summary linked to this article.

## Supplementary information

Supplementary Information

Peer Review File

Description of Additional Supplementary Files

Supplementary Movie 1

Supplementary Movie 2

Supplementary Movie 3

Supplementary Movie 4

Supplementary Movie 5

Supplementary Movie 6

Supplementary Movie 7

Supplementary Movie 8

Supplementary Movie 9

Supplementary Movie 10

Supplementary Movie 11

Supplementary Movie 12

Supplementary Movie 13

Supplementary Movie 14

Supplementary Movie 15

Supplementary Software 1-6

Reporting Summary

## Data Availability

IBT datasets are available at https://zenodo.org/badge/latestdoi/307904501.

## Code availability

Custom Python (Jupyter Notebook), MATLAB, and R source codes are provided with Supplementary Software 1–6 and available at https://github.com/coskunlab/Ion-Beam-Tomography.
