# Supplementary material for: Nanoscopic subcellular imaging enabled by ion beam tomography
Source: Nat Commun. 2021 Feb 4;12:789. doi: 10.1038/s41467-020-20753-5 (PMC7862654; doi:10.1038/s41467-020-20753-5)
Supplement: Supplementary file 1 — Supplementary Information [file 41467_2020_20753_MOESM1_ESM.pdf]

Supplementary Information for

Nanoscopic subcellular imaging enabled by Ion Beam Tomography

Ahmet F. Coskun[1,2,3,¥,*], Guojun Han[1,¥], Shambavi Ganesh[3,4], Shih-Yu Chen[1], Xavier Rovira Clavé[1], Stefan Harmsen[2], Sizun Jiang[1], Christian M. Schürch[1], Yunhao Bai[5], Chuck Hitzman[6], and Garry P. Nolan[1,*]
[1]Baxter Laboratory, Department of Microbiology and Immunology, Stanford University School of Medicine, Stanford, CA
[2]Department of Radiology, Molecular Imaging Program at Stanford, Stanford University School of Medicine, Stanford, CA
[3]Wallace H. Coulter Department of Biomedical Engineering, Georgia Institute of Technology and Emory University, Atlanta, GA, USA
[4]School of Electrical and Computer Engineering, Georgia Institute of Technology, Atlanta, GA, USA
[5]Department of Chemistry, Stanford University, Stanford, CA
[6]Department of Materials Science and Engineering, Stanford University, Stanford, CA
[¥] Equal contribution
Correspondence should be addressed to
* G.P.N. (gnolan@stanford.edu) and A.F.C. (ahmet.coskun@bme.gatech.edu)

Supplementary File	Title
**Supplementary Figure 1**	Deconvolution-Ion beam tomography pipeline
**Supplementary Figure 2**	Validation of Deconvolution-IBT using synthetic data.
**Supplementary Figure 3**	Lateral resolution analysis of ion images captured by Cesium gun source for Au coated Si samples at D1-3
**Supplementary Figure 4**	Lateral resolution analysis of ion images captured by Cesium gun source for Au coated Si samples at D1-2
**Supplementary Figure 5**	Lateral resolution analysis of ion images captured by Cesium gun source for Jurkat cell samples at 0.15-0.4 pA
**Supplementary Figure 6**	Resolution analysis of ion beam images captured by Oxygen gun source for Al coated Si samples
**Supplementary Figure 7**	Synthetic ion beam imaging and microscopy data
**Supplementary Figure 8**	Axial resolution analysis by a circular replication site
**Supplementary Figure 9**	Axial changes of sixty slices for the subcellular region
**Supplementary Figure 10**	Axial resolution by cell height divided by total scans
**Supplementary Figure 11**	SILM-IBT reconstruction pipeline
**Supplementary Figure 12**	Isotope encoded Nanotag size measurements
**Supplementary Figure 13**	Cross-section comparisons of image sum and SILM-IBT results.
**Supplementary Figure 14**	SILM precision analysis with 25 nm sigma in localization histogram
**Supplementary Figure 15**	Comparison of STORM and SILM principles (theory)
**Supplementary Figure 16**	Comparison of Raw-IBT and SILM-IBT in 2D and 3D
**Supplementary Figure 17**	Deep learning-based IBT pipeline
**Supplementary Figure 18**	Structural similarity values for HeLa DNA images
**Supplementary Figure 19**	Structural similarity values for Nalm6 DNA images
**Supplementary Figure 20**	Modeling of ion beam imaging parameters
**Supplementary Figure 21**	Pixel size effect of ion beam acquisition on analysis
**Supplementary Figure 22**	Signal to noise ratio analysis on multi-slice sums
**Supplementary Figure 23**	Registration patterns for IBT
**Supplementary Figure 24**	Schematic chain-mode for depth imaging by IBT

**Supplementary Figure 25**	Rapid depth etching by high current levels
**Supplementary Figure 26**	Elemental sensitivity analysis for Cesium and Oxygen
**Supplementary Figure 27**	Deconv-IBT results for chromatin images
**Supplementary Figure 28**	Fuzzy logic segmentation in a Nalm6 cell
**Supplementary Figure 29**	Fuzzy logic segmentation in a HeLa cell
**Supplementary Figure 30**	Volumetric chromatin density in HeLa and Nalm6
**Supplementary Figure 31**	Four-channel replication experiment (2-hours)
**Supplementary Figure 32**	A repeat of replication dynamics experiment (30-min)
**Supplementary Figure 33**	A repeat of replication dynamics experiment (2-hours)
**Supplementary Figure 34**	Statistics of overlaps in replication experiments
**Supplementary Figure 35**	Peak detection on a single replication site
**Supplementary Figure 36**	Replicate for transcription and replication in Nalm6
**Supplementary Figure 37**	Transcription and replication in a Nalm6 cell
**Supplementary Figure 38**	Long pulse (2-h) for transcription and replication
**Supplementary Figure 39**	Newly synthesized transcript localization after drug
**Supplementary Figure 40**	Transcription inhibitors during 2-h metabolic labeling
**Supplementary Figure 41**	Transcription signal reduction by α-amanitin
**Supplementary Figure 42**	Transcription and replication (color swap) in Nalm6
**Supplementary Figure 43**	3D mature transcription in HeLa by RNA FISH labeling
**Supplementary Figure 44**	Computational association and correlation analysis of Deconv-IBT data
**Supplementary Figure 45**	3D spatially resolved the k-means clustering algorithm on IBT data
**Supplementary Figure 46**	Label-free mass imaging in FFPE tissues
**Supplementary Figure 47**	Volumetric histology by IBT
**Supplementary Table 1**	Biotinylated FISH probe sequences for *ActB* detection
**Supplementary Methods**	

Deconvolution-Ion beam tomography pipeline

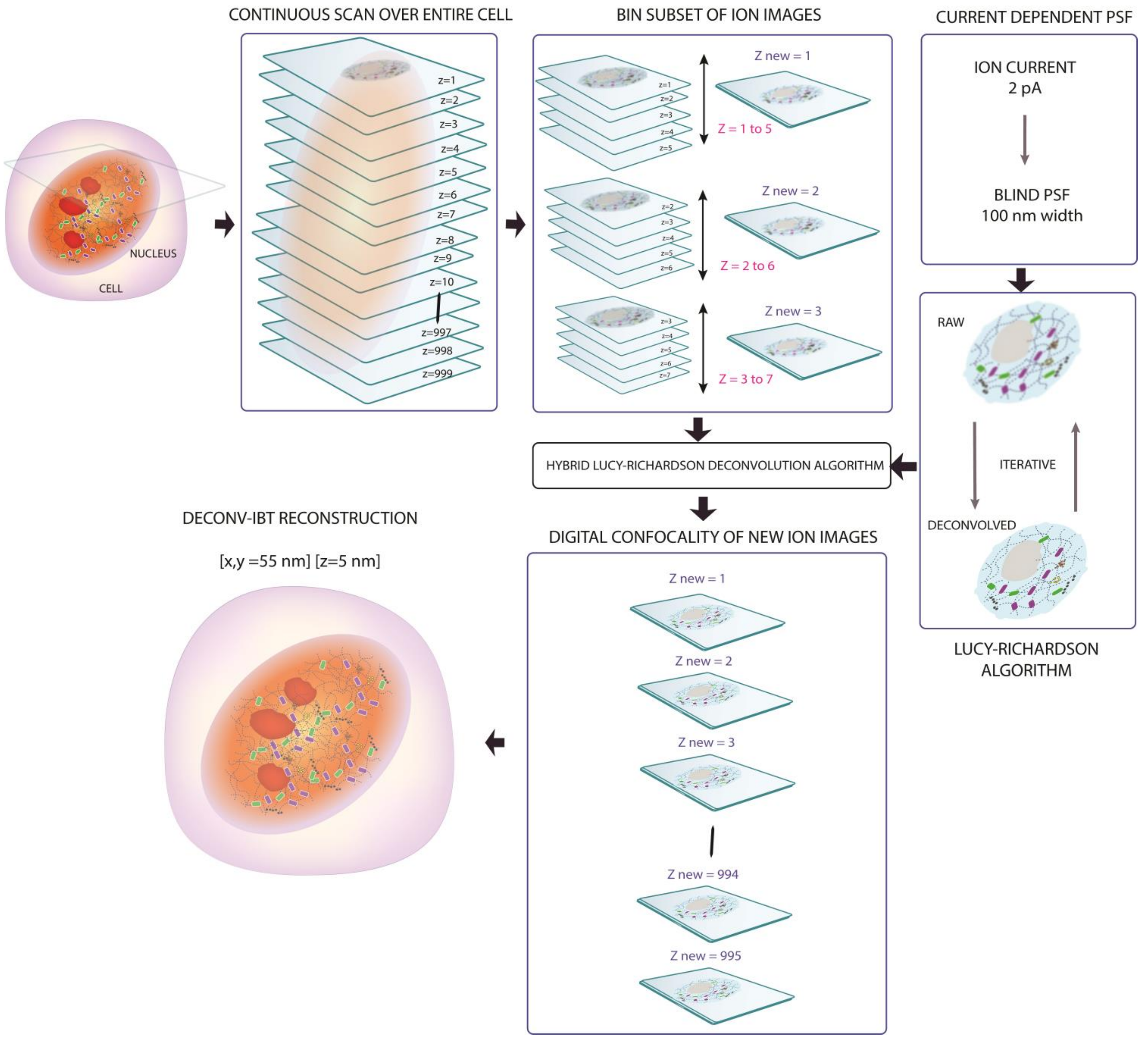

Supplementary Figure 1. IBT deconvolution pipeline.

Ion images are acquired across 1,000 depth sections.

A subset of raw ion scans is binned (in this example, over five slices) to generate a new ion image with a high signal to noise ratio (SNR) and less noise. This process is incremented by one slice at a time, providing a new ion image series with a similar dimension. The last few slices are skipped due to windowing.

A current-dependent point spread function is calculated for the deconvolution process.

Each ion image is then deblurred by an iterative Lucy Richardson deconvolution algorithm.

Five iterations were optimum for typical ion images but must be optimized based on the SNR of the images.

The resultant sharper ion images are then stitched over axial direction for error-free reconstructions.

Credit: Images were edited from Designua, Timonina, and Alejo Miranda/Shutterstock.com.

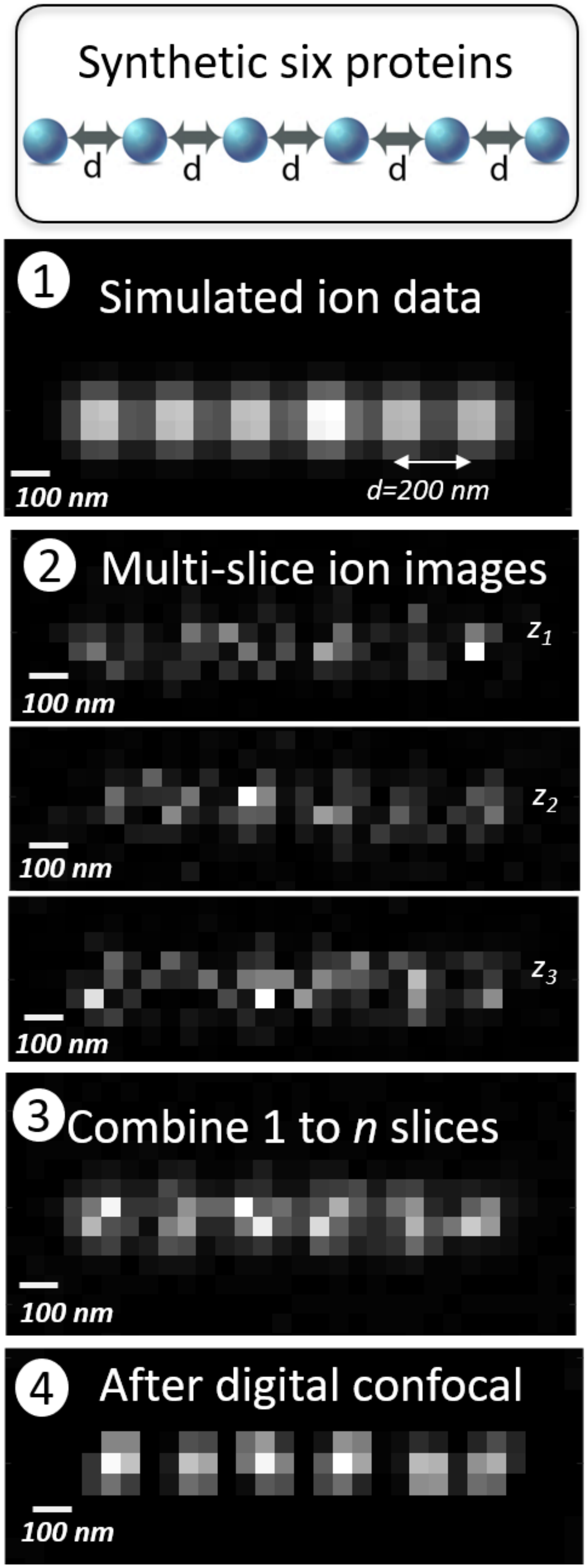

Synthetic six proteins
d
d
d
d
d
1 Simulated ion data
100 nm
d=200 nm
2 Multi-slice ion images
z_1
100 nm
z_2
100 nm
z_3
100 nm
3 Combine 1 to *n* slices
100 nm
4 After digital confocal
100 nm

Supplementary Figure 2. Validation of Deconvolution-IBT using synthetic ion beam data.

① Six simulated protein molecules separated by 200 nm were imaged with 50-nm pixel size and 120-nm ion beamwidth.

② The original ion image was contaminated by digital multiplicative noise, providing simulated ion images at three different depths (z_1, z_2, and z_3).

③ Multiple depth images of 1 to *n* slices (with sliding windows of 5 slices) were summed.

④ Binned ion images were digitally deconvolved to deblur the objects, resolving the neighboring pixels. The resultant image agrees well with the distribution in the synthetic image, validating the accuracy of reconstructions.

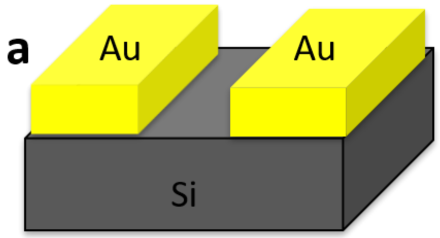

c

	Deconv-IBT	Raw-IBT
D1_3	55 nm	213 nm

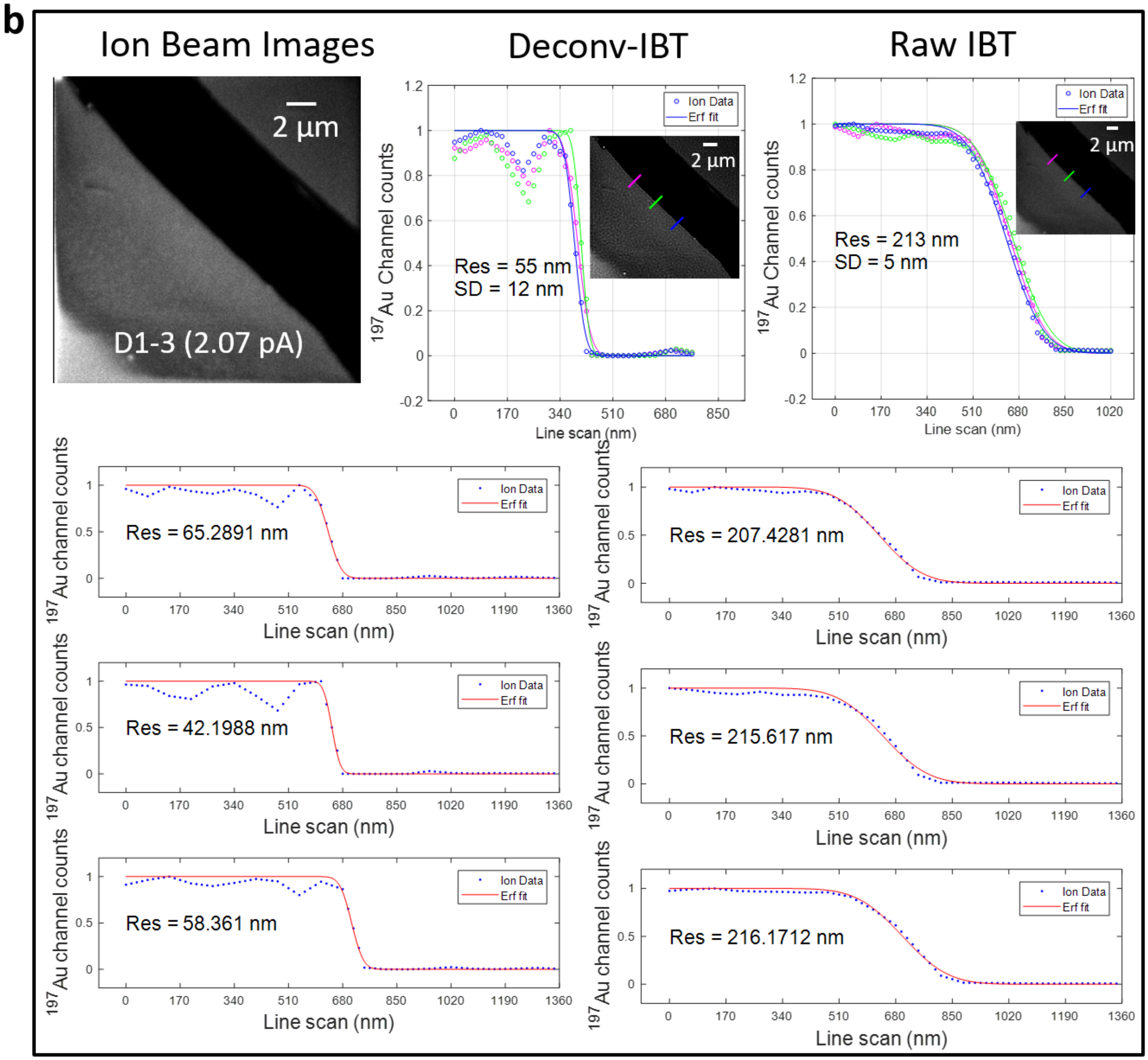

Supplementary Figure 3. Deconvolution-IBT framework results in down to 55-nm lateral resolution.

a Au-coated silicon substrates were used to quantify spatial resolution in ion beam images generated by a cesium source.

b Ion beam images were acquired at 2.07 using D1-3 aperture. Three cross-sections at the edge of Au-Si were used to quantify spatial resolution. Insets show processed ion images with blue, green, and magenta line plots, respectively.

c Summary of resolution limits of raw IBT and deconvolution IBT images, providing a 4-fold enhancement in the lateral resolution of SIMS imaging.

To quantify resolution, error-function (Erf) analysis was done by normalizing the ion intensity levels to 1 and fitting the following function:

(fit= 0.5 * (1±erf(sqrt(2) * (x -x0)/ w))

Where x is the position of the edge scan, x0 is the center position, and w is the width of the ion beam that was used for image scanning.

A similar analysis was performed in the Passarelli et al. Analytical Chemistry 2014, Development of an Organic Lateral Resolution Test Device for Imaging Mass Spectrometry.

a

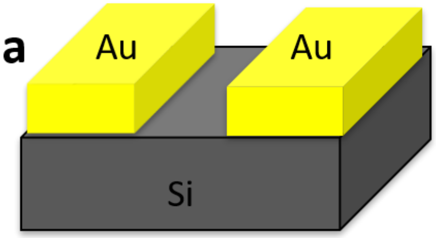

c

	Deconv-IBT	Raw-IBT
D1_2	67 nm	249 nm

b

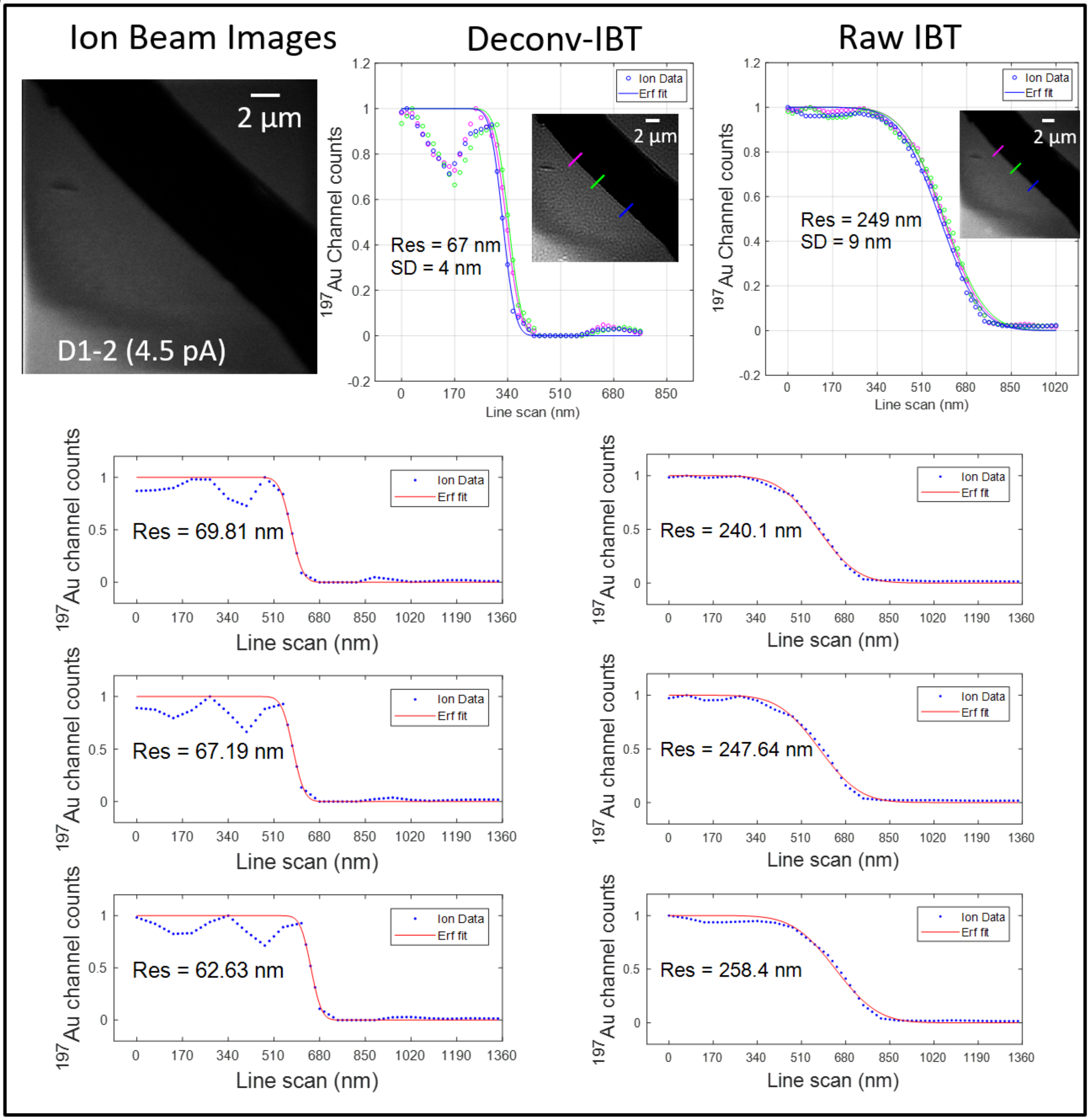

Supplementary Figure 4. Deconvolution-IBT framework results in down to 67-nm lateral resolution with a higher current level.

a Au-coated silicon substrates were used to quantify spatial resolution in ion beam images generated by a cesium source.

b Ion beam images were acquired at 4.5 using D1-2 aperture. Three cross-sections at the edge of Au-Si were used to quantify spatial resolution. Insets show processed ion images with blue, green, and magenta line plots, respectively.

c Summary of resolution limits of raw IBT and deconvolution IBT images, providing a 4-fold enhancement in the lateral resolution of SIMS imaging.

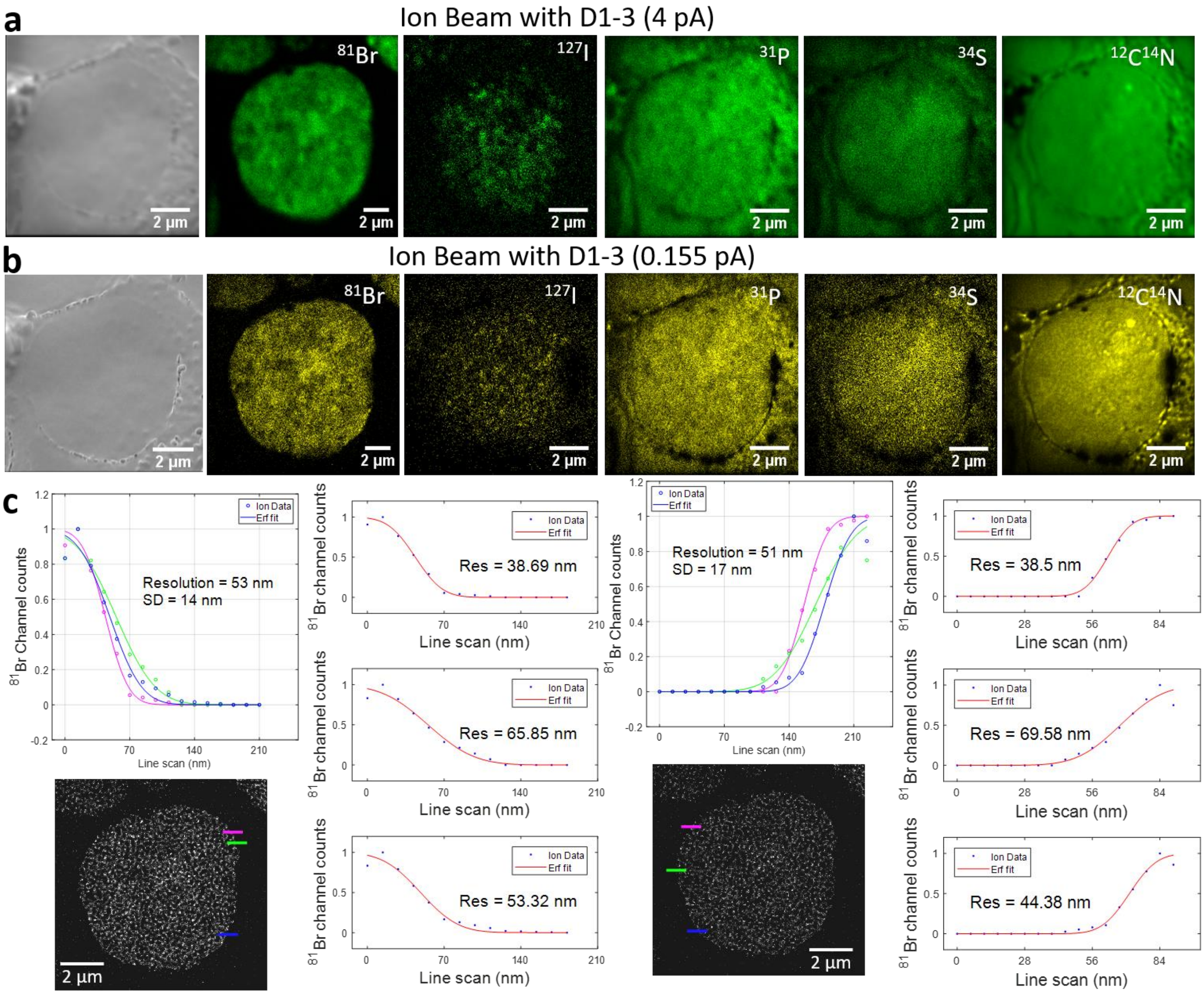
a
Ion Beam with D1-3 (4 pA)
81Br
127I
31P
34S
12C14N
2 µm
b
Ion Beam with D1-3 (0.155 pA)
81Br
127I
31P
34S
12C14N
2 µm
c
Resolution = 53 nm
SD = 14 nm
81Br Channel counts
Line scan (nm)
Ion Data
Erf fit
Res = 38.69 nm
Res = 65.85 nm
Res = 53.32 nm
81Br channel counts
Resolution = 51 nm
SD = 17 nm
Res = 38.5 nm
Res = 69.58 nm
Res = 44.38 nm
2 µm

Supplementary Figure 5. Six-color ion beam imaging experiment for endogenous elements (^{12}C, ^{14}N, ^{34}S: total proteins; ^{31}P: DNA backbone) and metabolite labels and replication loci (labeled with ^{127}I-dU for 24 hours and ^{81}Br-dU for 30 minutes) in Jurkat cells.

a Seven channel ion beam image of a Jurkat cell by D1_3 setting corresponding to 4 pA of ion current.

b The same cell was imaged at 0.155 pA current, highest resolution setting with the smallest feasible ion beamwidth.

c Three distinct line scans were measured across the right and left edges of the image detected at the ^{81}Br channel. Blue, red, and magenta colors correspond to the line scan positions on the cell image and cross-section plots. In both edges, the distance through which the %88 ion signal drops to 12% of the maximum yielded 53 nm±13 nm (STD) spatial resolution, agreeing well with the gold edge on the silicon substrate experiment shown in **Supplementary Fig. 3**.

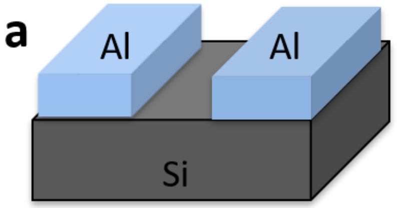

c

	Deconv-IBT	Raw-IBT
D1_3	498 nm	522 nm

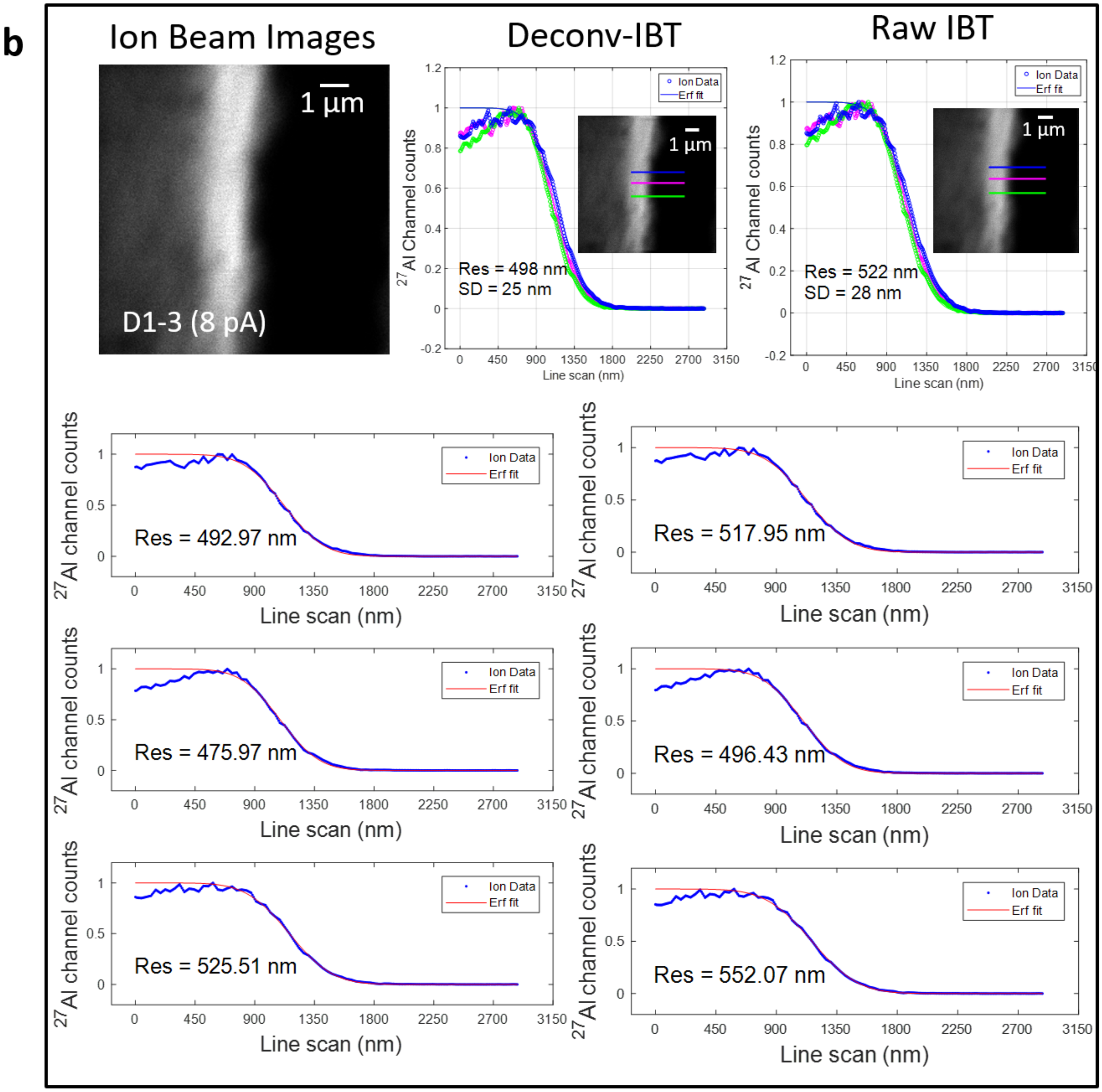

Supplementary Figure 6. Resolution characterization for ion images acquired using an oxygen source using deconvolution and analysis parameters similar to those described in **Supplementary Fig. 3**.

a An Al-coated silicon substrate was used to measure the edge profile.

b Ion beam images were acquired at 8 pA using D1-3 aperture. Three cross-sections at the edge of Al-Si were used to quantify spatial resolution. Insets show processed ion images with blue, green, and magenta line plots for 8pA.

c Summary of resolution limits of raw MIBI and reconstructed IBT images by deconvolution analysis of MIBI data (Deconvolution-IBT).

Spatial resolution levels down to 498 nm were achieved. Larger iteration numbers will reduce these resolution levels while image artifacts may increase as a result of the processing. Due to the large beam width, computation faces challenges to decouple overlapping ion signals in digital processing. Had the edge of this test substrate been sharper more stringent resolution criteria could have been obtained. Oxygen beam experiments are not the focus of this paper, but the IBT algorithm works on these images with decent resolution enhancements. Sub-500 nm resolution levels are sufficient for the study of tumor samples obtained from biopsies.

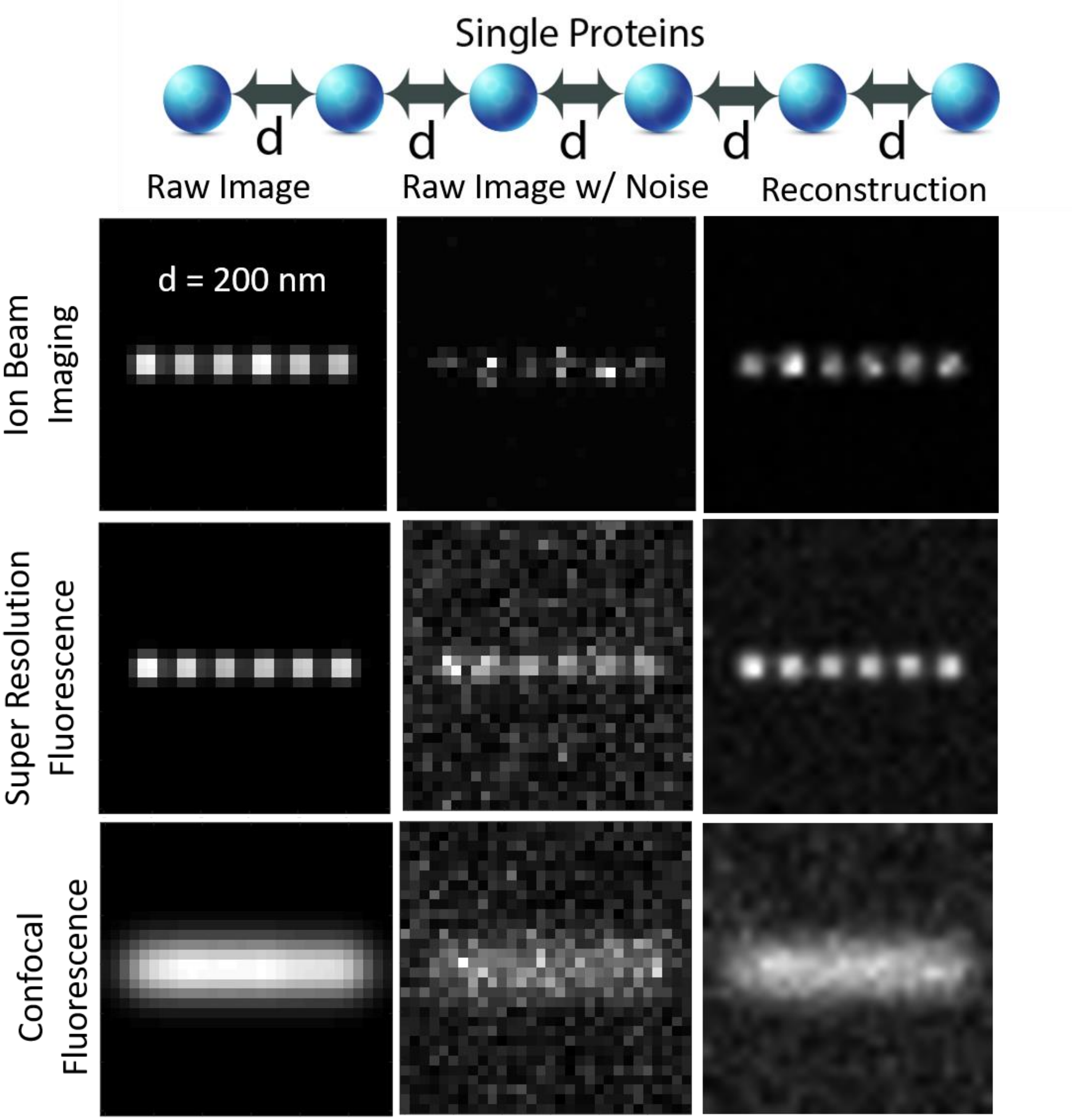
Single Proteins
d
d
d
d
d
Raw Image
Raw Image w/ Noise
Reconstruction
d = 200 nm
Ion Beam Imaging
Super Resolution Fluorescence
Confocal Fluorescence

Supplementary Figure 7. A simulated array was used to show that Deconvolution-IBT bridges the gap between wide-field confocal imaging and super-resolution optical microscopy.

Six protein signatures with a separation of 200 nm were generated for

Ion beam imaging (PSF: 120 nm; pixel size: 50 nm)
Super-resolution fluorescence imaging (PSF: 50 nm; pixel size: 50 nm)
Confocal fluorescence microscopy (PSF: 250 nm; pixel size: 100 nm).

Super-resolution was assumed to be a STORM imaging platform for a computational PSF of 50 nm after reconstructions (real PSF size and localization process are not detailed here).

The noise was then measured from experimental images and used to contaminate each synthetic image.

Ion beam images have higher noise variation than fluorescence images.

The IBT mathematical pipeline was then validated after reconstruction of the noisy ion beam and fluorescence images.

Axial resolution determination by a single replication fork

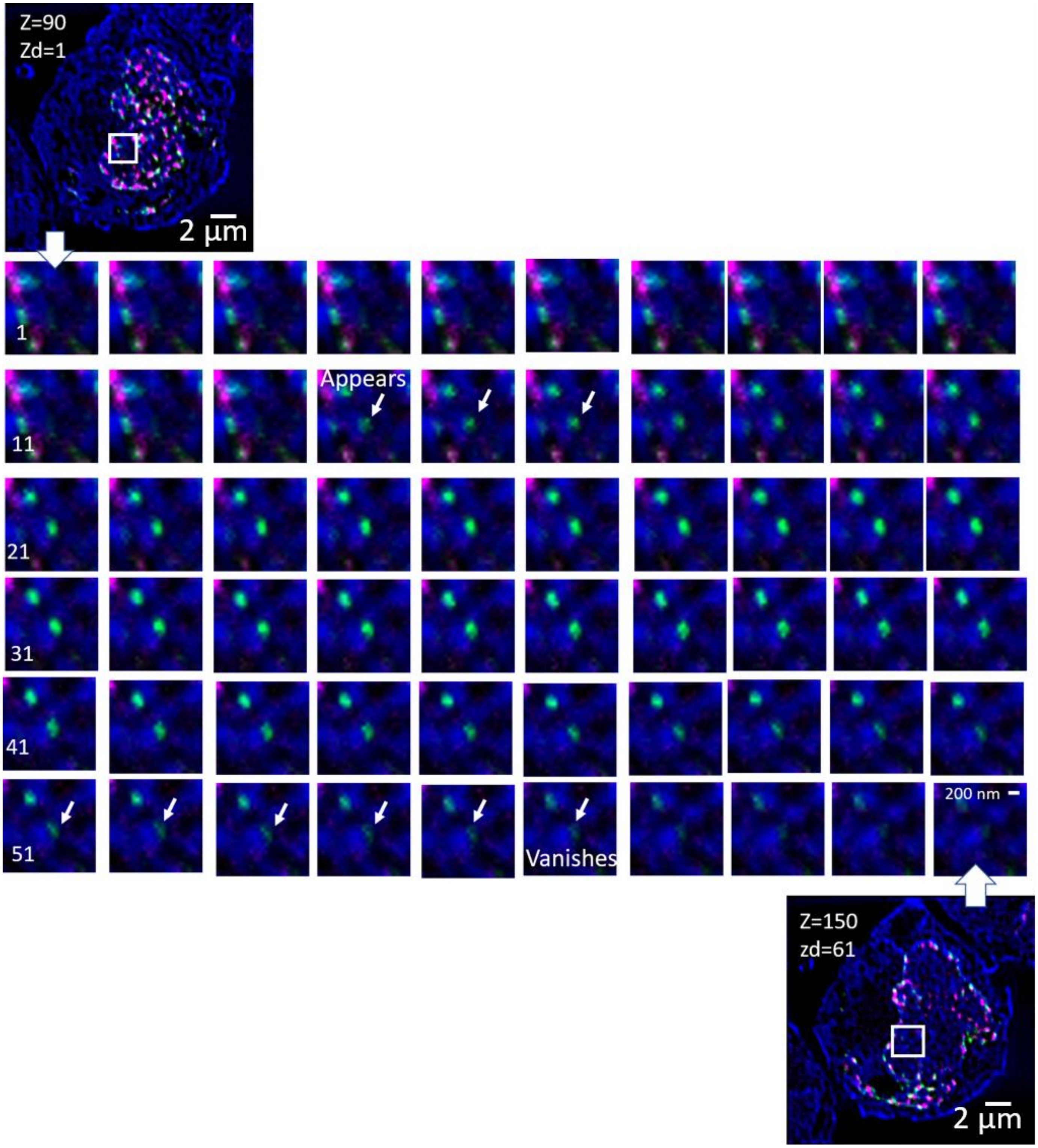

Supplementary Figure 8. Estimation of axial resolution by replication fork imaging.

^{31}P channel (largely chromatin, referred to as DNA) in blue, ^{127}I-dU was a replication site in green, and ^{81}Br-dU was another replication site in magenta. Each replication corresponds to a result of 30 minutes of incorporation.

Sixty slices of two replication forks in ^{127}I-dU channel reveal a 3-pixel replication site (size=175 nm in one direction) appearing around the 15th slice and vanishing around the 55th slice.

From this image, the axial resolution was estimated to be approximately 5 nm as the 175-nm replication site extends over 40 slices.

High-resolution depth imaging of replication forks by ion beam tomography

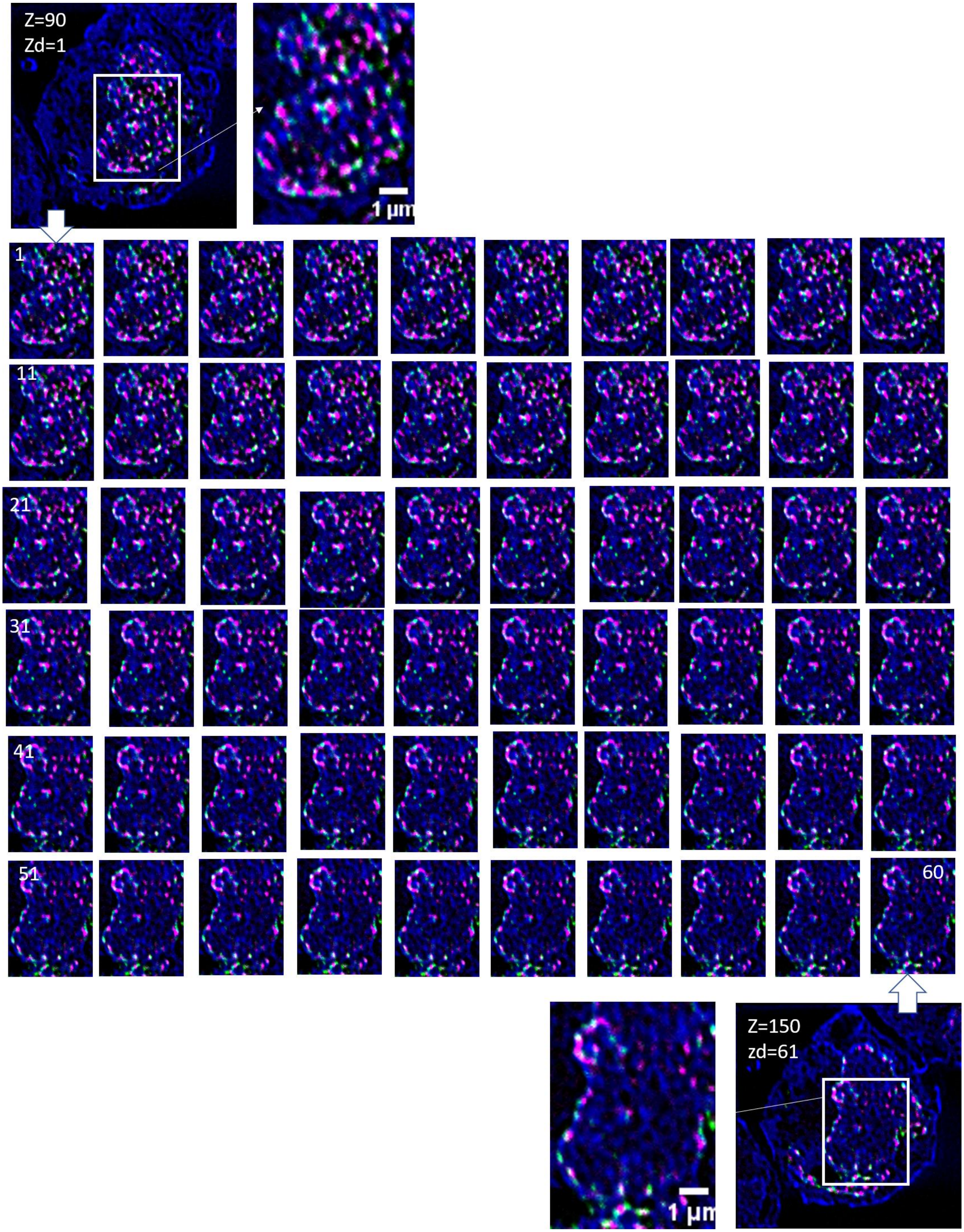

Supplementary Figure 9. Estimation of axial resolution by imaging of a single cell.

Similar to the previous figure: ^{31}P channel (blue), ^{127}I-dU (green), and ^{81}Br-dU (magenta).

Sixty consecutive depth images of a Nalm6 cell shows slight differences at every scan with 5-nm slice thickness indicative of approximately 5-nm axial resolution for IBT.

The tomographic thickness of a cell for axial resolution analysis

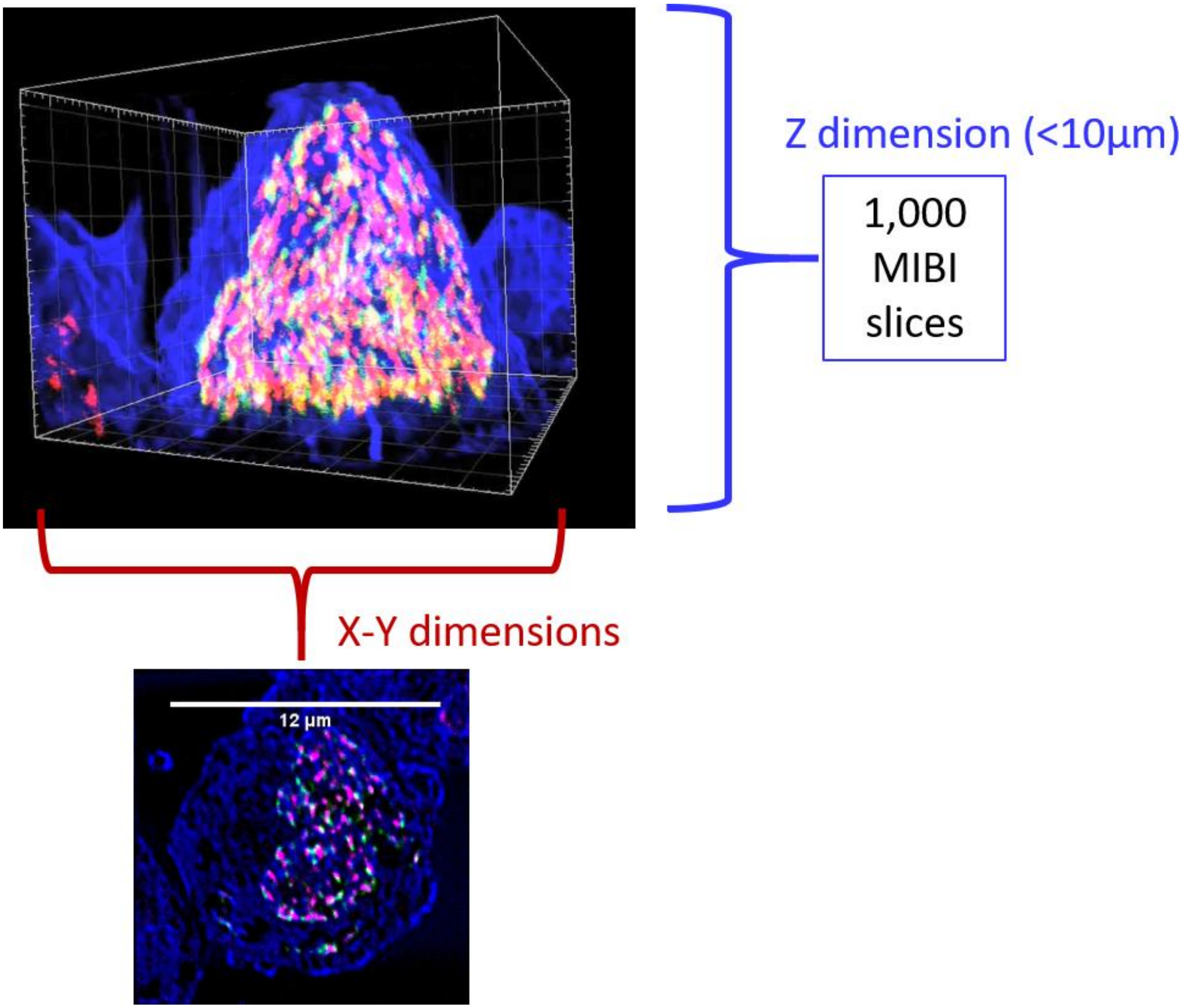

Supplementary Figure 10. Estimation of axial resolution by imaging of a single cell.

One thousand scans were etched through a Nalm6 cell. The lateral extent of the cell is between 10 and 12 µm.

Due to the dehydration process and spin coating of cells during preparation, the axial extent is between 5 and 10 µm, yielding an estimate of 5-10 nm axial resolution.

Localization based (SILM) Ion beam tomography pipeline

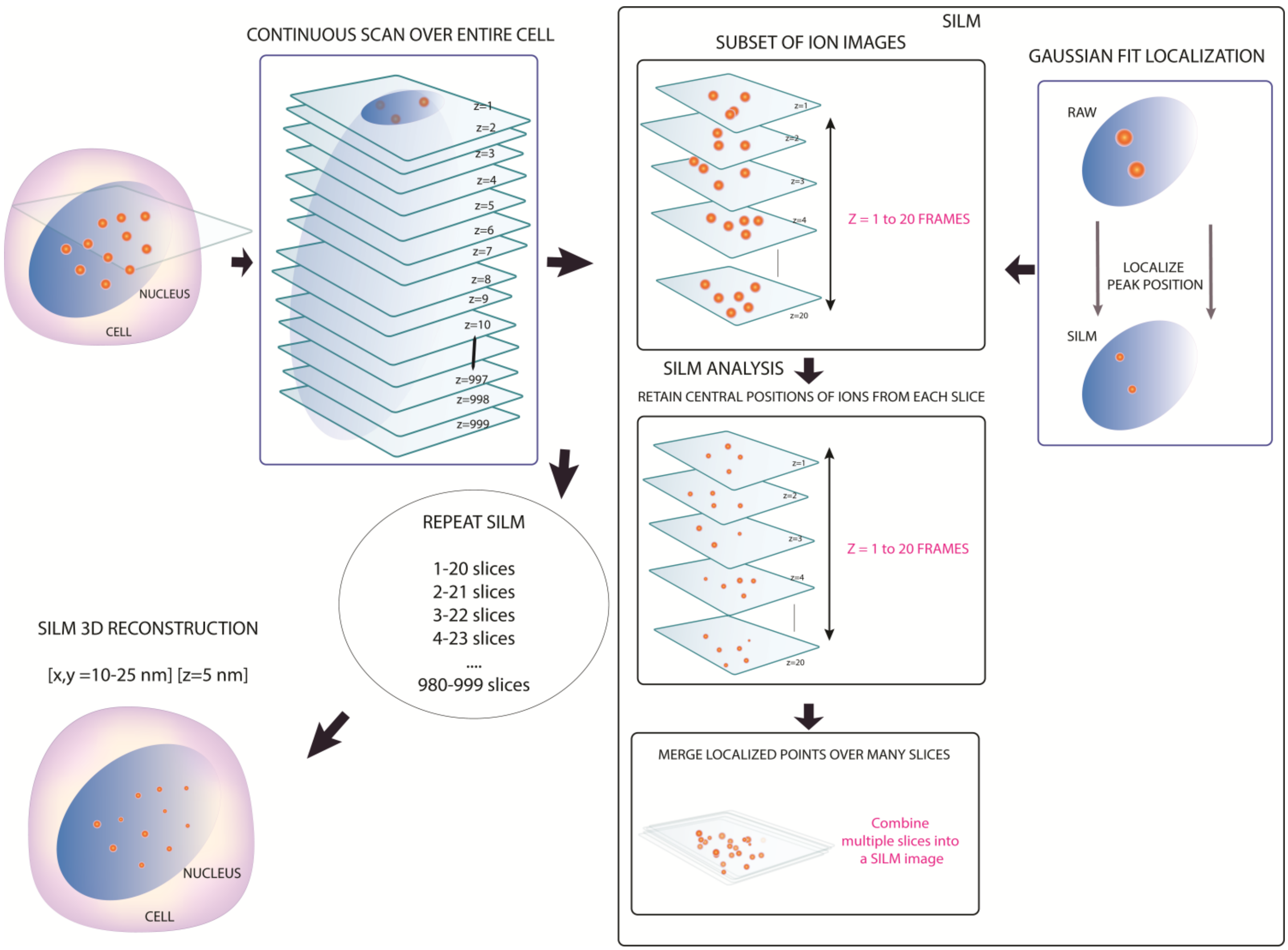

Supplementary Figure 11. Inspired by the STORM analysis in fluorescence, we use the localization of isolated ion molecules in the NanoSIMS scans from the entire cell.

A subset of image stacks (e.g., 20-100) is used for SILM analysis.

Each slice exhibits blurred ion distributions due to the finite ion-beam width. And thus, central positions of isolated ions were calculated using Gaussian fits. These localized positions can be as small as 5 nm with the smaller size of the ion beam than the optical beams.

Localized points were then merged across the image stacks, providing a high-precision SILM ion beam image.

Similar to deconvolution-IBT, by using a sliding window, we can then map 3D cell volume with a high-precision SILM image at each section.

Credit: Images were edited from Designua, Timonina, and Alejo Miranda/Shutterstock.com.

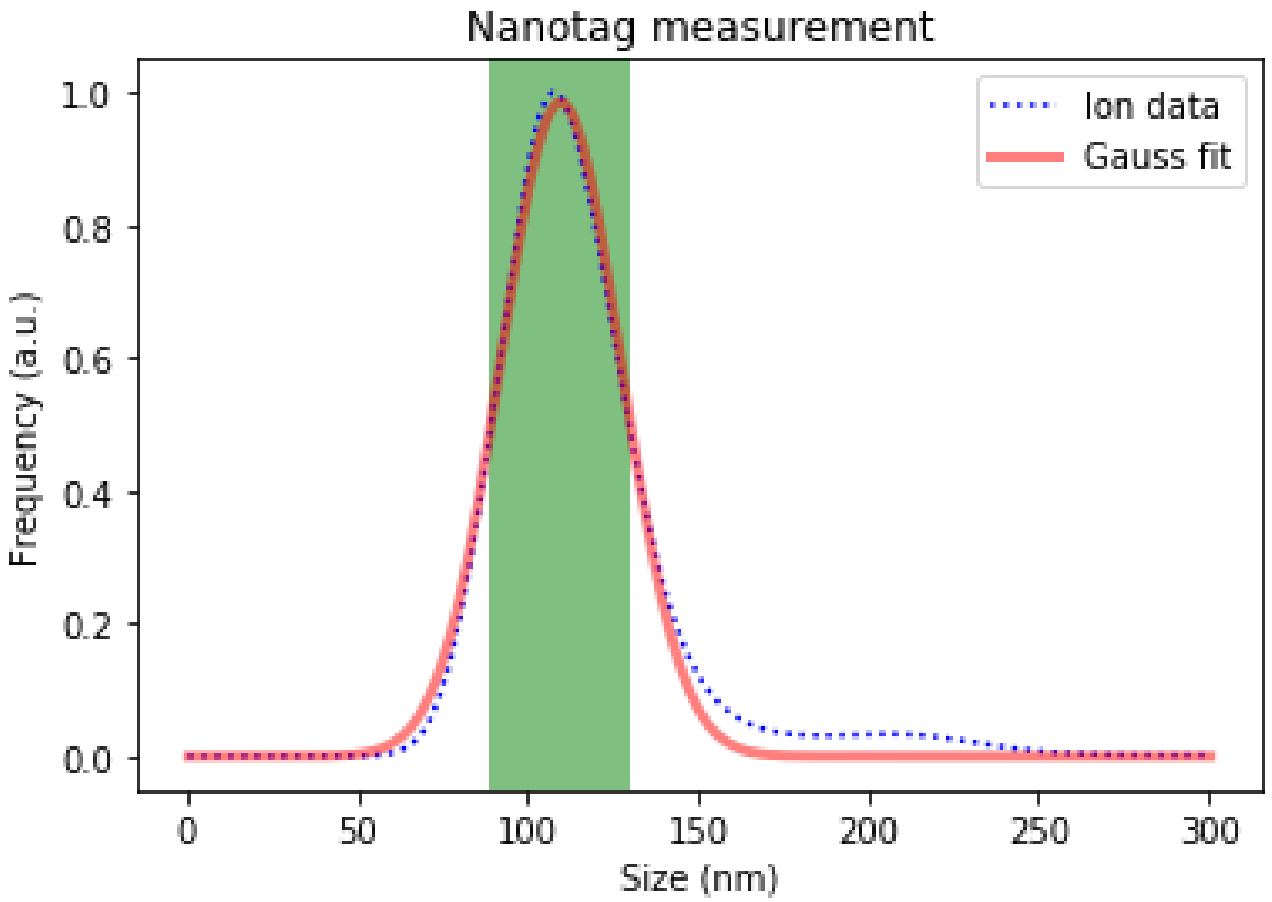

Nanotag measurement
1.0
0.8
0.6
0.4
0.2
0.0
Frequency (a.u.)
0
50
100
150
200
250
300
Size (nm)
Ion data
Gauss fit

Supplementary Figure 12. The size distribution of nanotags yielded a fit function centered around 109.8 nm ± 17.6 nm using dynamic light scattering measurement in a solution.

The Gaussian fit was defined as follows.

*Gaus(x,a,x0,sigma): a*exp(-(x-x0)**2/(2*sigma**2))*

Blue dots: Raw measurements from the light scattering.

Redline: Fit line from Gaussian plot over the data.

Green region: The FWHM (41.46 nm) for the Gaussian fit.

The analysis was done by Jupyter notebook, Python pipeline.

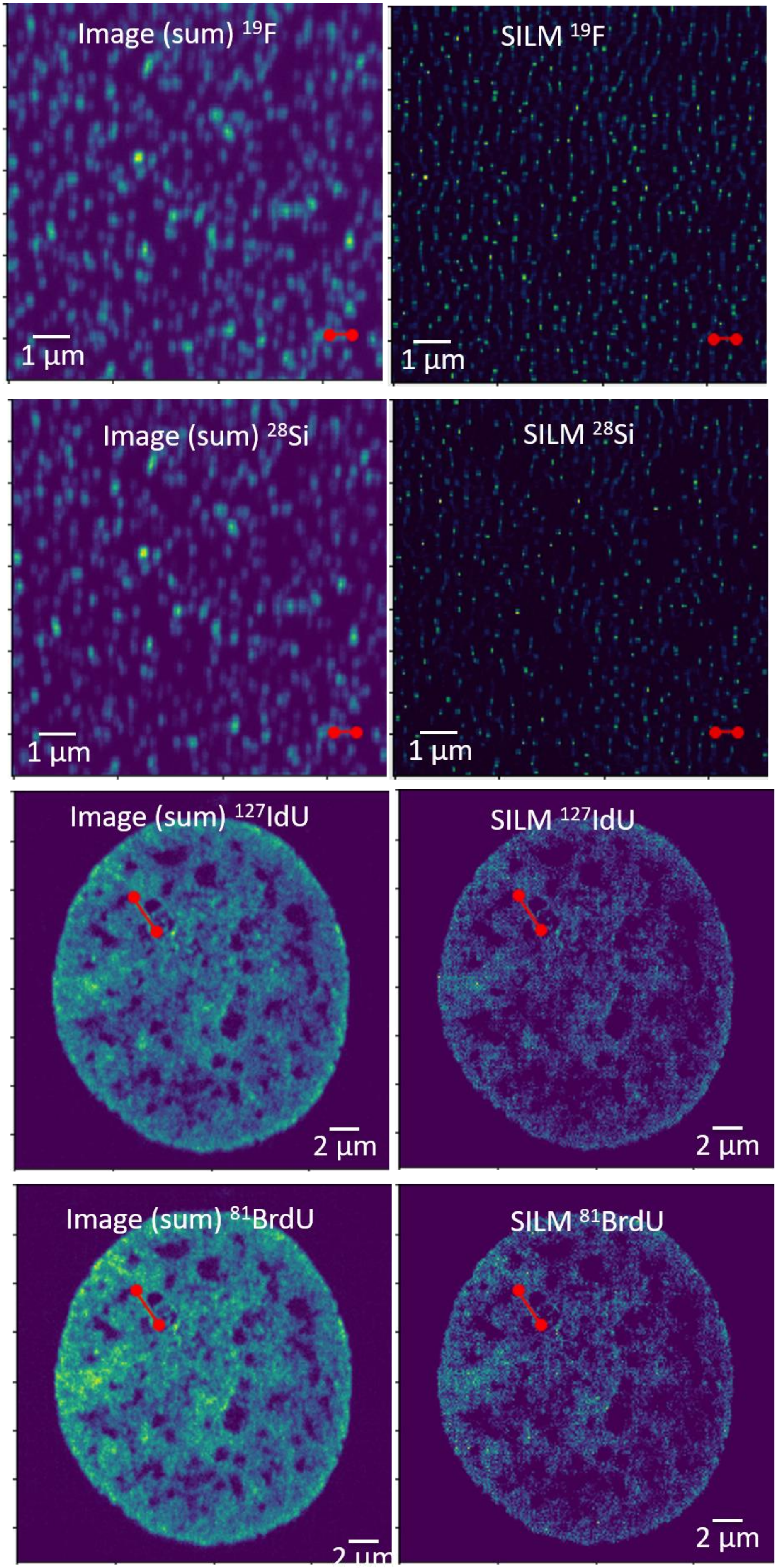

Image (sum) 19F
SILM 19F
1 µm
1 µm
Image (sum) 28Si
SILM 28Si
1 µm
1 µm
Image (sum) 127IdU
SILM 127IdU
2 µm
2 µm
Image (sum) 81BrdU
SILM 81BrdU
2 µm

Supplementary Figure 13. Cross-sections across image sum and SILM-IBT results.

Features showed resolution improvements in the line scans for both nanotags (top two rows) and chromatin (bottom two rows).

SILM PRECISION

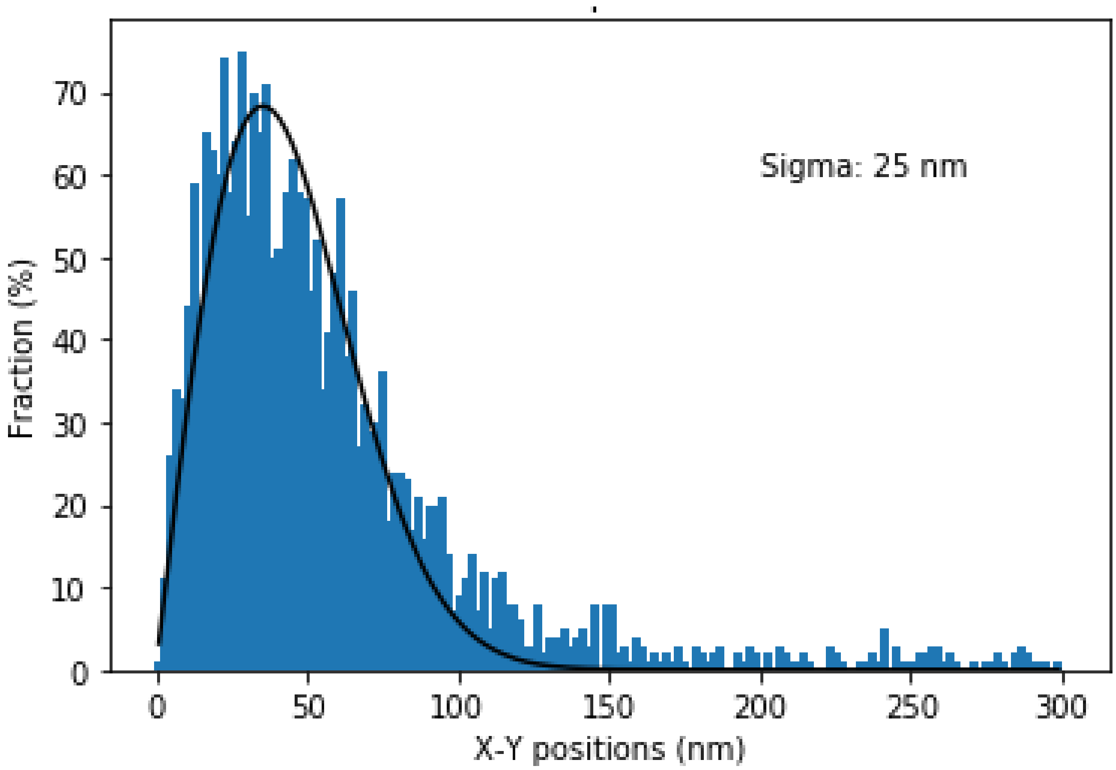

Supplementary Figure 14. Localization accuracy analysis of the Secondary ion beam localization microscopy (SILM) reconstructions.

Precision measurement was done on the 100-nm nanotag image series.

Localized positions of fitted ion signatures yielded a histogram with a sigma (σ) value of 25-nm in X-Y directions from 25 images.

This precision accuracy suggests the practical spatial enhancement level of SILM-IBT.

STORM	SILM
"signal" blinks	Localize subset of ions
Reducing reagents	Vacuum conditions
$\sigma_{STORM} = \frac{\omega_{fluo}}{\sqrt{N_{photons}}}$	$\sigma_{SILM} = \frac{\omega_{ion\ beam}}{\sqrt{N_{ions}}}$
Resolution=10-50 nm	Resolution=5-25 nm

Supplementary Figure 15. Significant differences in localization analysis in fluorescence and ion beam microscopy.

(1) Signal blinks whereas ion beam imaging localizes a subset of ions at each z-scan.

(2) Special reagents needed for STORM but SILM is recorded in vacuum conditions

(3) Precision accuracy is dependent on beam width and the number of photons/ions.

(4) The theoretical precision limit of SILM can be much smaller than optical imaging, as it starts with a smaller ion beam-width (50-100 nm), given enough ion counts are detected.

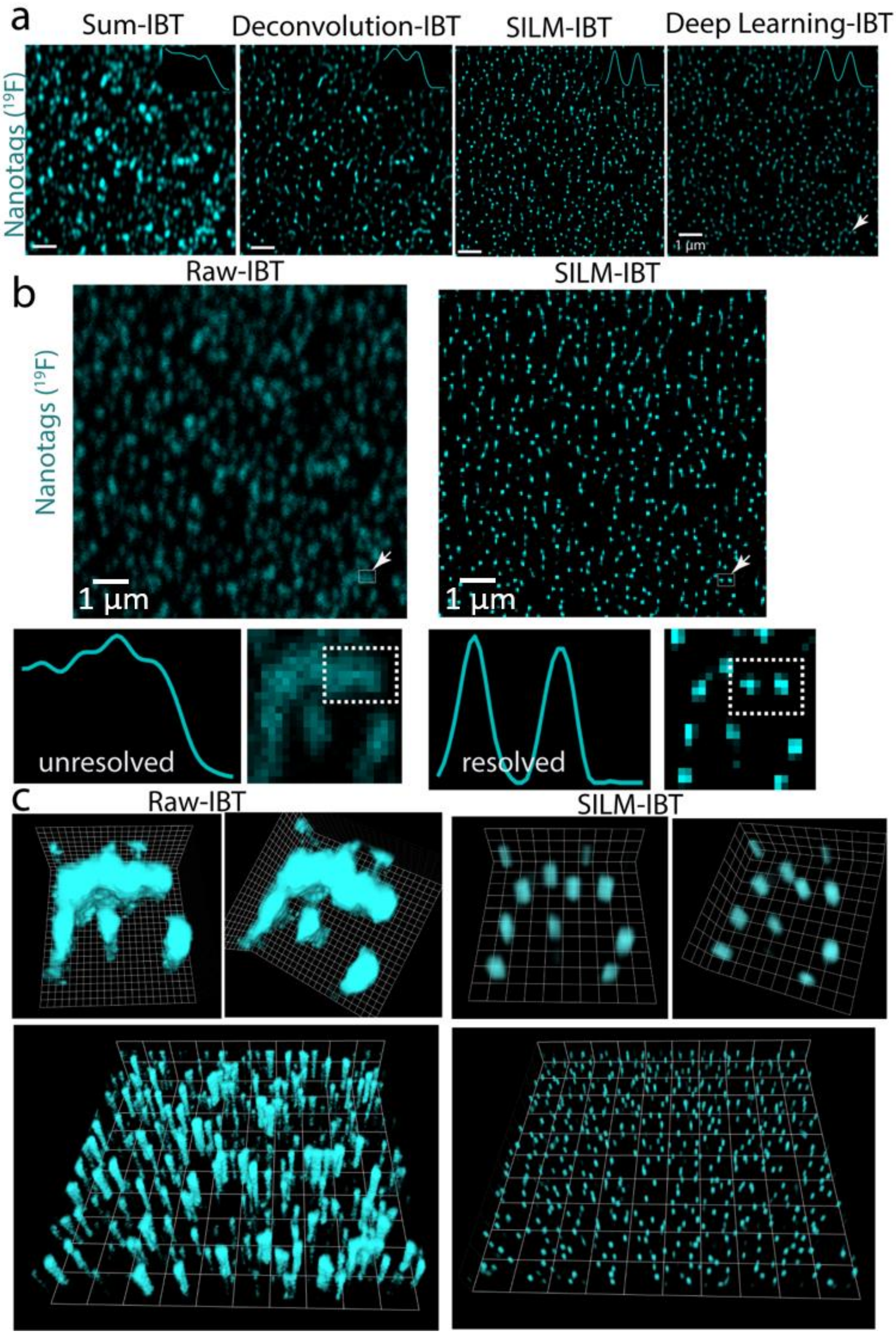
a
Sum-IBT
Deconvolution-IBT
SILM-IBT
Deep Learning-IBT
Nanotags (19F)
1 µm
b
Raw-IBT
SILM-IBT
Nanotags (19F)
1 µm
1 µm
unresolved
resolved
c
Raw-IBT
SILM-IBT

Supplementary Figure 16. Raw-IBT and SILM-IBT comparisons.

a Summary of nanotags analyzed by four distinct methods from Fig. 1d.

b Raw-IBT scan of ^{19}F nanotags and its corresponding image from SILM-IBT.

Cross-sections across neighboring nanotags demonstrate that nanotag pairs were not resolved in the Raw IBT images, but resolved in the SILM-IBT localization-based reconstructions.

c 3D representation of Raw-IBT images across 26 slices, once again, fails to resolve the nanotags. However, SILM-IBT resolves bead groups in this large cluster.

(First row) Smaller regions of nanotags for Raw-IBT and SILM-IBT, respectively.

(Second row) The larger region of nanotags for comparisons.

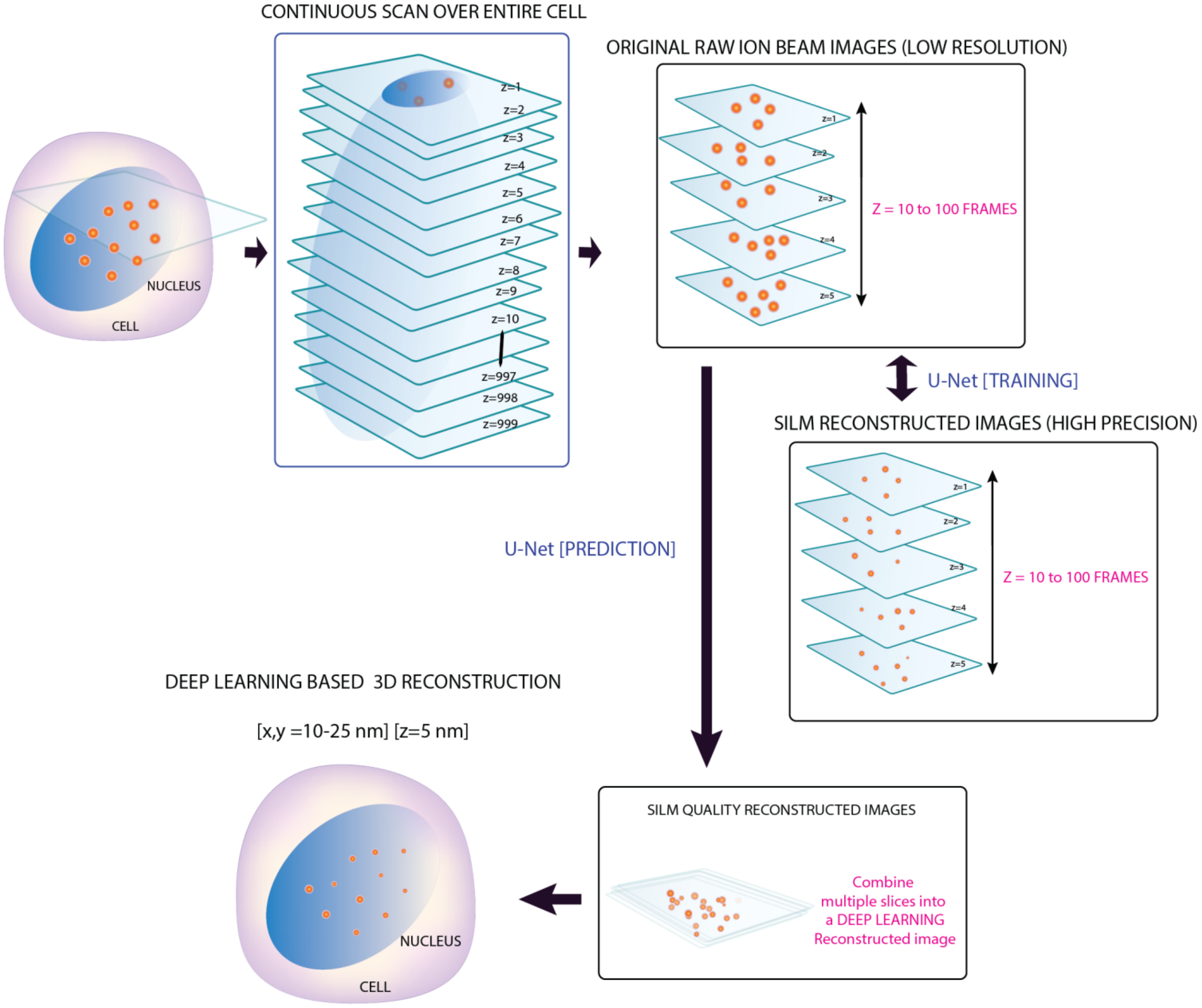
CONTINUOUS SCAN OVER ENTIRE CELL
NUCLEUS
CELL
z=1
z=2
z=3
z=4
z=5
z=6
z=7
z=8
z=9
z=10
z=997
z=998
z=999
ORIGINAL RAW ION BEAM IMAGES (LOW RESOLUTION)
Z = 10 to 100 FRAMES
U-Net [TRAINING]
SILM RECONSTRUCTED IMAGES (HIGH PRECISION)
Z = 10 to 100 FRAMES
U-Net [PREDICTION]
DEEP LEARNING BASED 3D RECONSTRUCTION
[x,y =10-25 nm] [z=5 nm]
SILM QUALITY RECONSTRUCTED IMAGES
Combine
multiple slices into
a DEEP LEARNING
Reconstructed image
NUCLEUS
CELL

Supplementary Figure 17. Deep learning analysis of ion beam 3D stacks.

Step 1, Image generation: Organize 3D data and the selected subset of ion beam images. Partition each image into multiple (100-200) smaller windows with 32x32 pixel sizes.

Step 2, Training: The raw ion beam images (low-resolution) were used as “input” images and the SILM reconstructed image (high-precision) as “target” for the same field-of-view.

Step 3, Prediction: Using the training model, an arbitrary image acquired at a similar condition was then processed to “predict” a high-resolution network “output”.

Deep learning-based IBT allows parameter-free reconstruction of features similar to the SILM details. Besides, the reconstructions are independent of the density of the target biomolecules.

Credit: Images were edited from Designua, Timonina, and Alejo Miranda/Shutterstock.com.

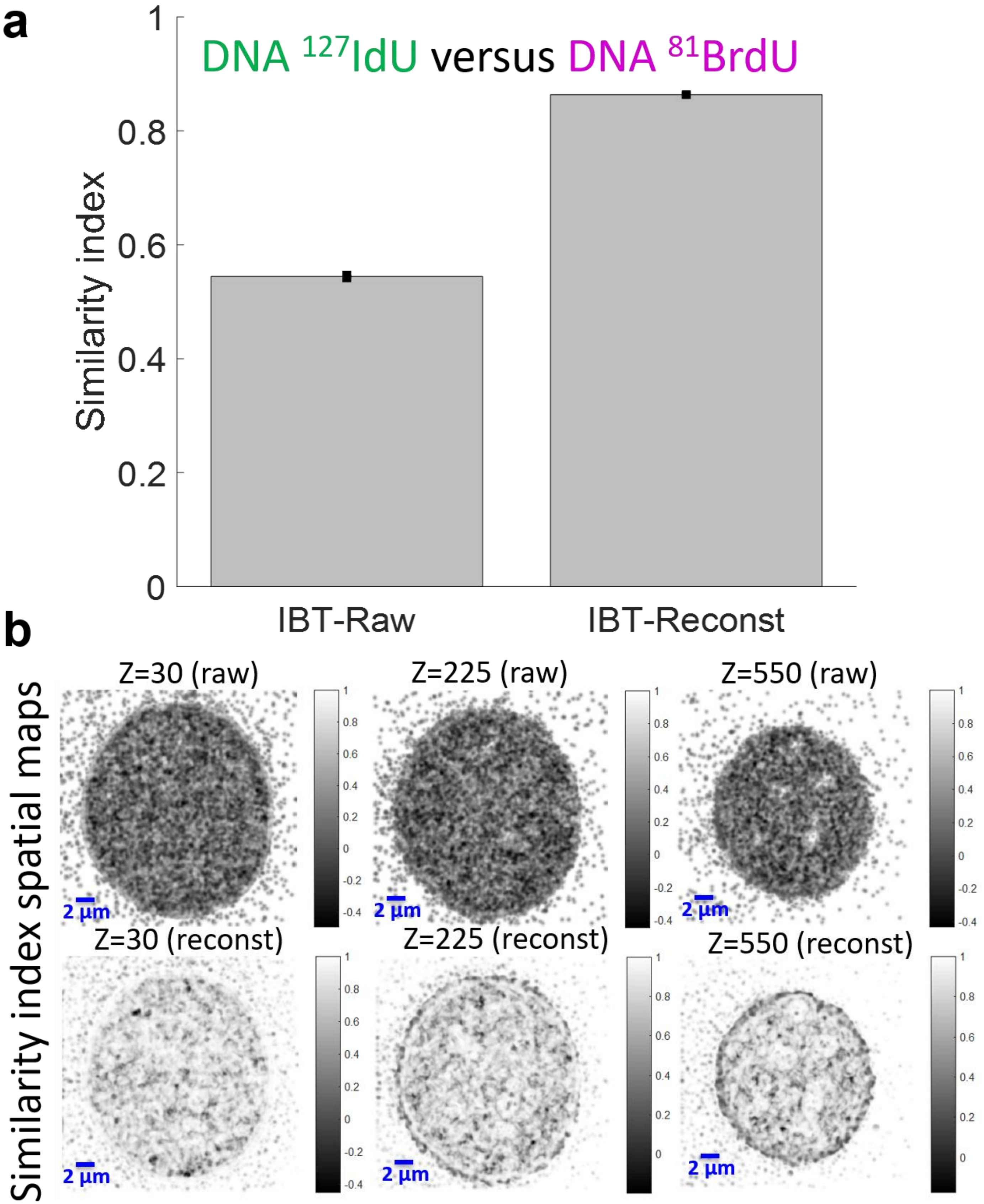
a
DNA 127IdU versus DNA 81BrdU
Similarity index
1
0.8
0.6
0.4
0.2
0
IBT-Raw
IBT-Reconst
b
Similarity index spatial maps
Z=30 (raw)
Z=225 (raw)
Z=550 (raw)
Z=30 (reconst)
Z=225 (reconst)
Z=550 (reconst)
2 µm

Supplementary Figure 18. Structural similarity index (SSIM) values were used to quantify the "similarity" measure of DNA images at the ^{127}I-dU and ^{81}Br-dU channels for the raw and reconstructed ion images that were presented in Fig. 3.

a Reconstructed DNA images exhibit significantly higher similarity (SSIM: 0.86 ±SE 0.001) compared to the raw DNA images (0.54 ±SE 0.0037).

IBT-raw: SSIM values for measuring the similarity of the raw DNA images from ^{127}I-dU and ^{81}Br-dU.

IBT-reconst: SSIM values for calculating the similarity of the reconstructed images from ^{127}I-dU and ^{81}Br-dU.

n = 737 sections were used in the bar graph with a mean and standard error of SSIM values.

b Spatial map of SSIM quantification in DNA images for 30th, 225th, and 550th ion beam tomographic slices.

(Top row) SSIM index values per pixel were plotted as a spatial map for raw DNA images. Most pixels average around dark grey SSIM values, suggesting high dissimilarity.

(Bottom row) SSIM spatial values for reconstructed DNA images. Most pixels span around light grey SSIM values, providing a high degree of similarity between images.

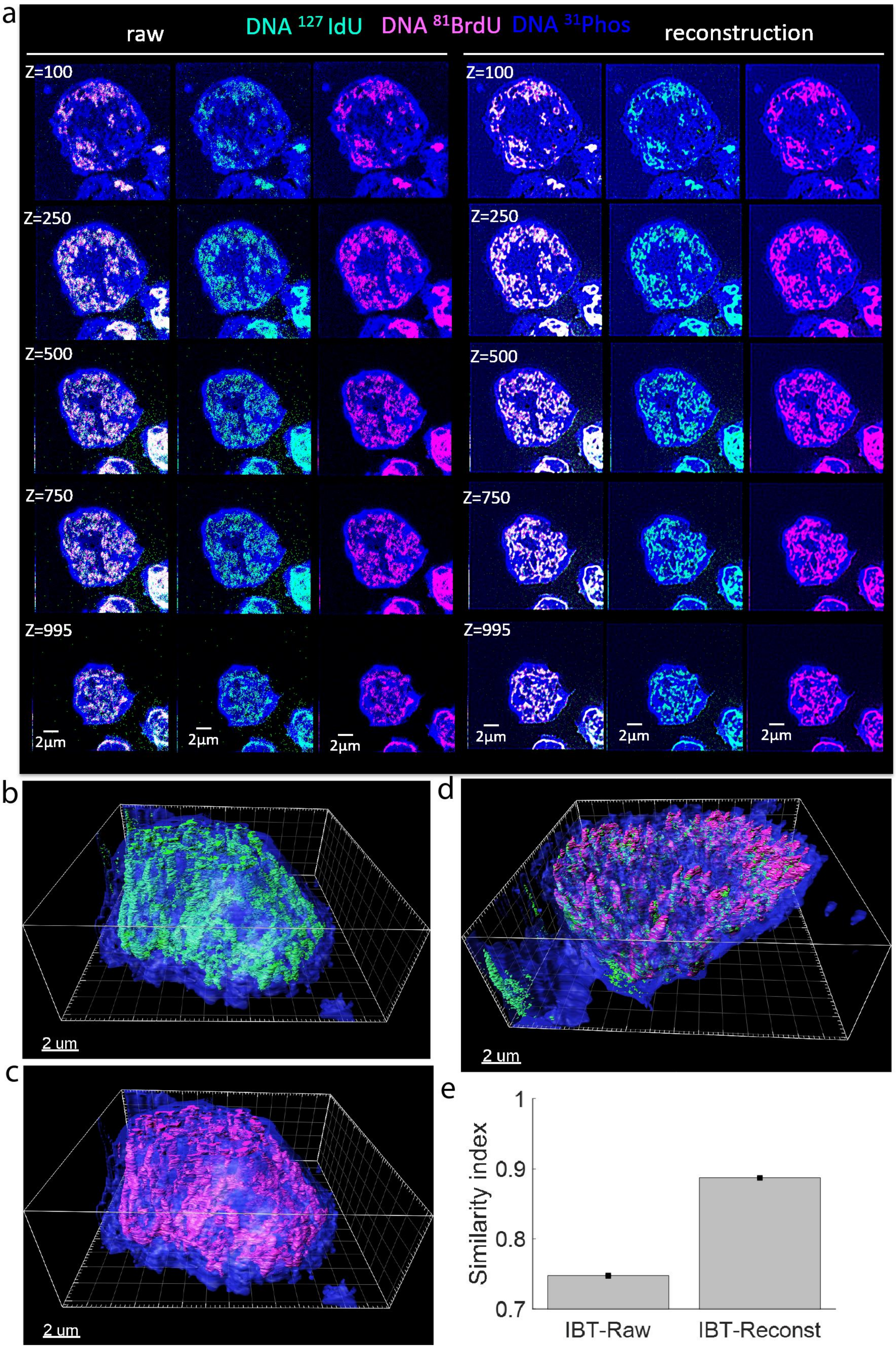
a
raw
DNA 127 IdU
DNA 81BrdU
DNA 31Phos
reconstruction
Z=100
Z=250
Z=500
Z=750
Z=995
2μm
b
c
d
e
2 um
Similarity index
1
0.9
0.8
0.7
IBT-Raw
IBT-Reconst

Supplementary Figure 19. SSIM values of DNA images at the ^{127}I-dU and ^{81}Br-dU channels for the raw and Deconvolution-IBT ion images in a Nalm6 cell.

a Similar to Figure 3, but for Nalm6 cells. Raw images of replicated DNA (green and magenta) and largely chromatin (blue) are noisy and reconstructed images show spatially resolved patterns in the chromatin.

b 3D renders of Nalm6 ion beam tomogram. Replicated DNA by ^{127}I-dU (green) and largely chromatin by ^{31}P (blue).

c 3D visualization of Nalm6 with Replicated DNA by ^{81}Br-dU (magenta) and large chromatin by ^{31}P (blue).

d Inverted 3D position of the same Nalm6 cell with ^{127}I-dU (green), ^{81}Br-dU (magenta), and chromatin by ^{31}P (blue).

e Reconstructed chromatin images exhibit higher similarity (SSIM: 0.88 ±SE 0.001) compared to the raw DNA images (0.74 ±SE 0.001).

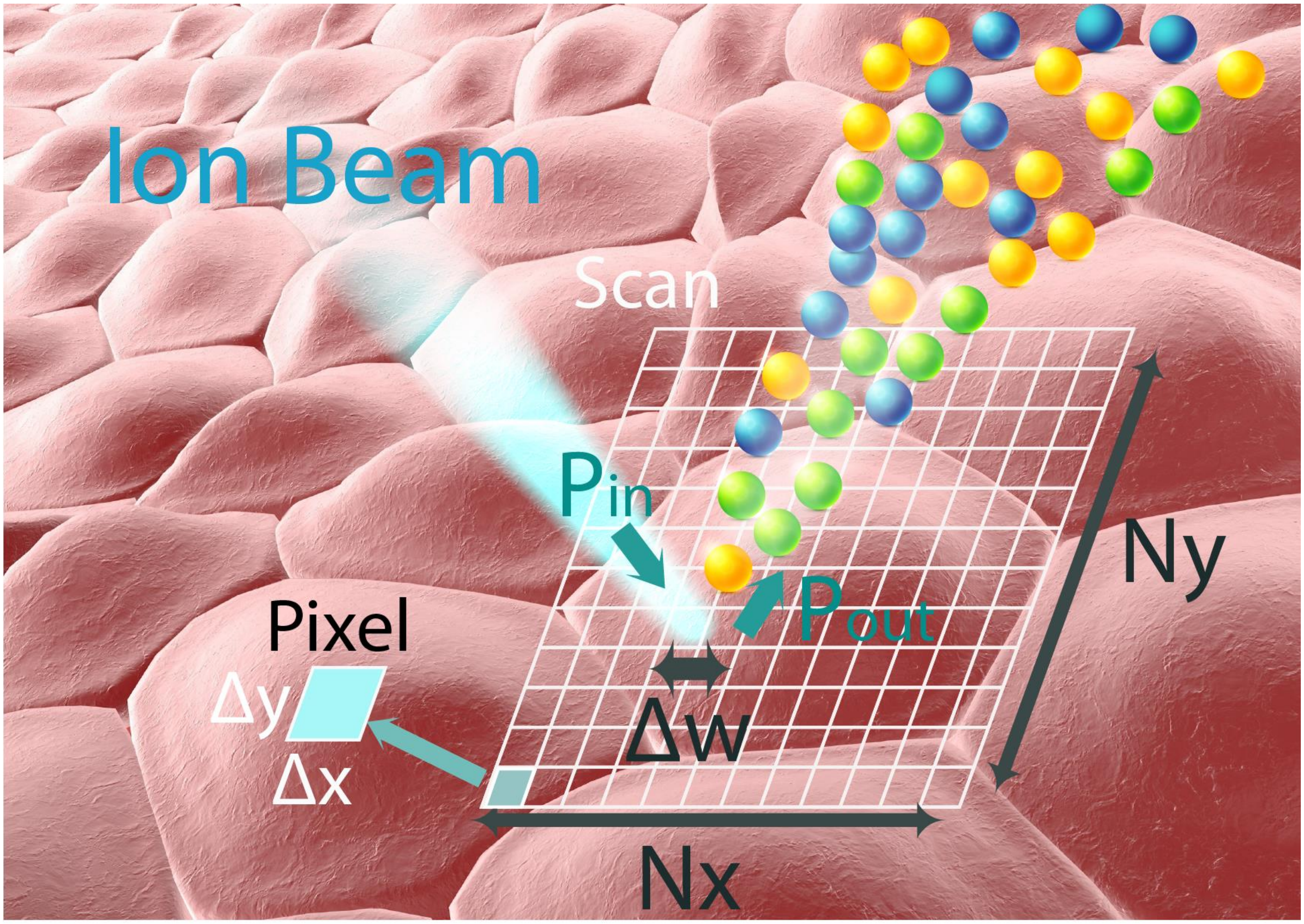
Ion Beam
Scan
P_{in}
P_{out}
Ny
Pixel
Δy
Δx
Δw
Nx

Supplementary Figure 20. Modeling of ion beam imaging parameters.

Ion beam source (blue) corresponds to either Cesium or Oxygen source, impinging on the sample of interest.

After focusing down to sub-100 nm, incoming ion signal (P_{in}) to the sample within a small subcellular voxel.

Total secondary ions extracted from the sample (P_{out}) and were calculated to be 3%. Distinct color (round objects) correspond to different mass labels.

Pixel size dimensions Δx and Δy of these ion beam images: In the range of 50-150 nm in both dimensions.

The total number of pixels per axis, N_x x N_y pixels, typically 256x 256 pixels, 512x 512 pixels.

Ion beamwidth of Δw that is determined by the ion beam optics and aperture settings: 50-500 nm wide.

Credit: Image was edited from Kateryna Kon/Shutterstock.com.

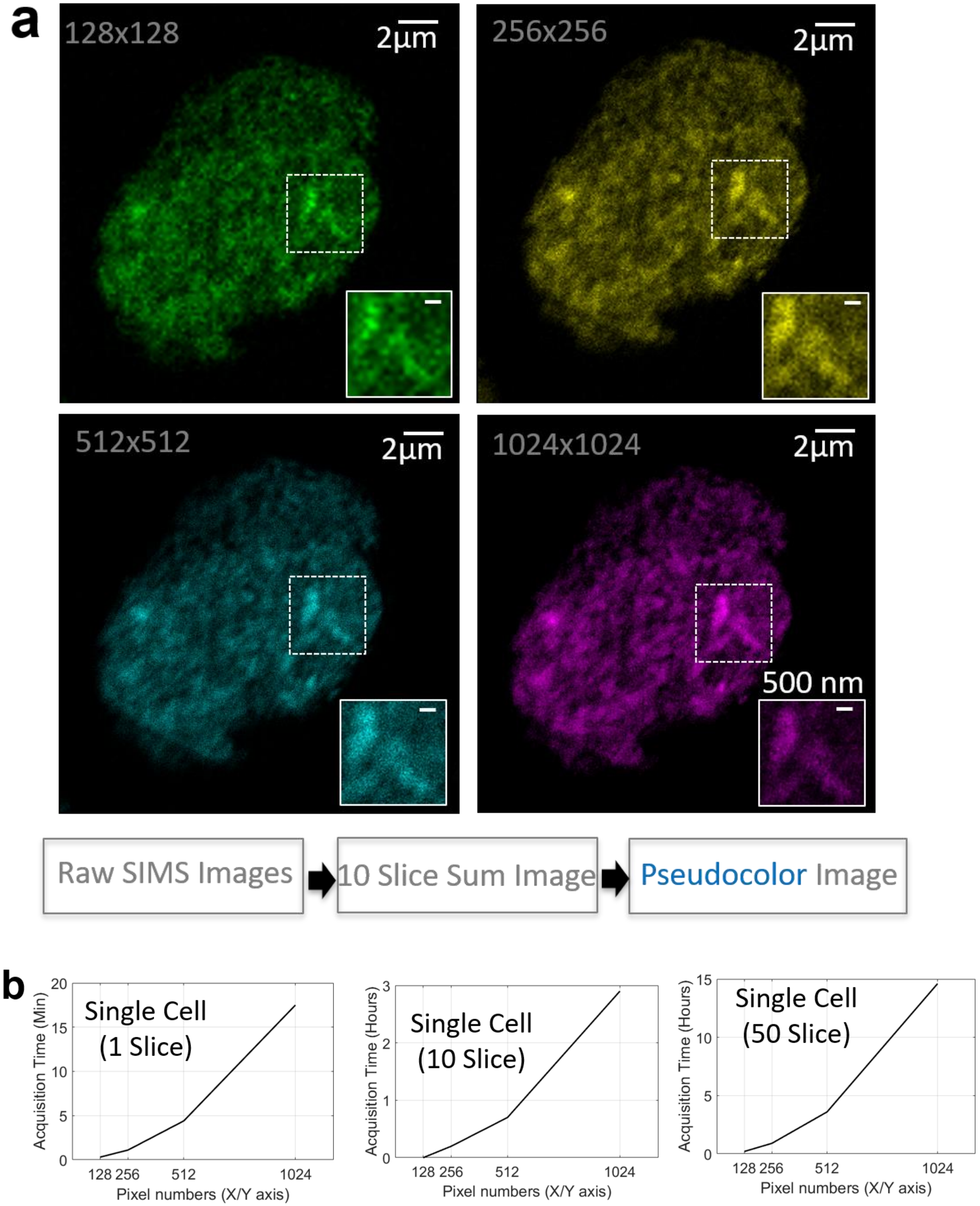
a
128x128
2µm
256x256
2µm
512x512
2µm
1024x1024
2µm
500 nm
Raw SIMS Images
10 Slice Sum Image
Pseudocolor Image
b
Single Cell
(1 Slice)
Acquisition Time (Min)
Pixel numbers (X/Y axis)
Single Cell
(10 Slice)
Acquisition Time (Hours)
Pixel numbers (X/Y axis)
Single Cell
(50 Slice)
Acquisition Time (Hours)
Pixel numbers (X/Y axis)

Supplementary Figure 21. Pixel size effect for ion beam imaging.

a HeLa cell DNA imaged by ^{127}I channel after 24-hour incubation with ^{127}I-dU to label DNA.

Four different scans with unique pixel size values were captured for the same cell.

A 25-µm window was imaged and divided into 128 x 128 pixels, 256 x 256 pixels, 512 x 512 pixels, or 124 x 1024 pixels.

Dividing the imaging window by the number of pixels yielded 156 nm, 78 nm, 39 nm, and 19 nm pixel sizes, respectively.

Ten consecutive frames were then summed and pseudocolored in MATLAB. These images were captured with a 100-nm PSF width. Imaging pixel size must be smaller than 100 nm to record details of interest.

Thus, the 156-nm pixel size image was not able to resolve details whereas 78-, 39-, and 19-nm pixel sizes provided similar quality images. Sub-50-nm pixel size exposes the same area to ion beams multiple times, creating an oversampling effect in the images.

b Image acquisition time scales with numbers of pixels and slices:

Single slice takes more than 15 minutes at 1024x1024 sampling.
Ten slices require up to 3 hours for the same pixel size.
Fifty slices consume about 15 hours with the same pixel dimensions.

A window size of 256 x 256 pixels is optimal for acquisition time, sampling accuracy, and spatial resolution capability.

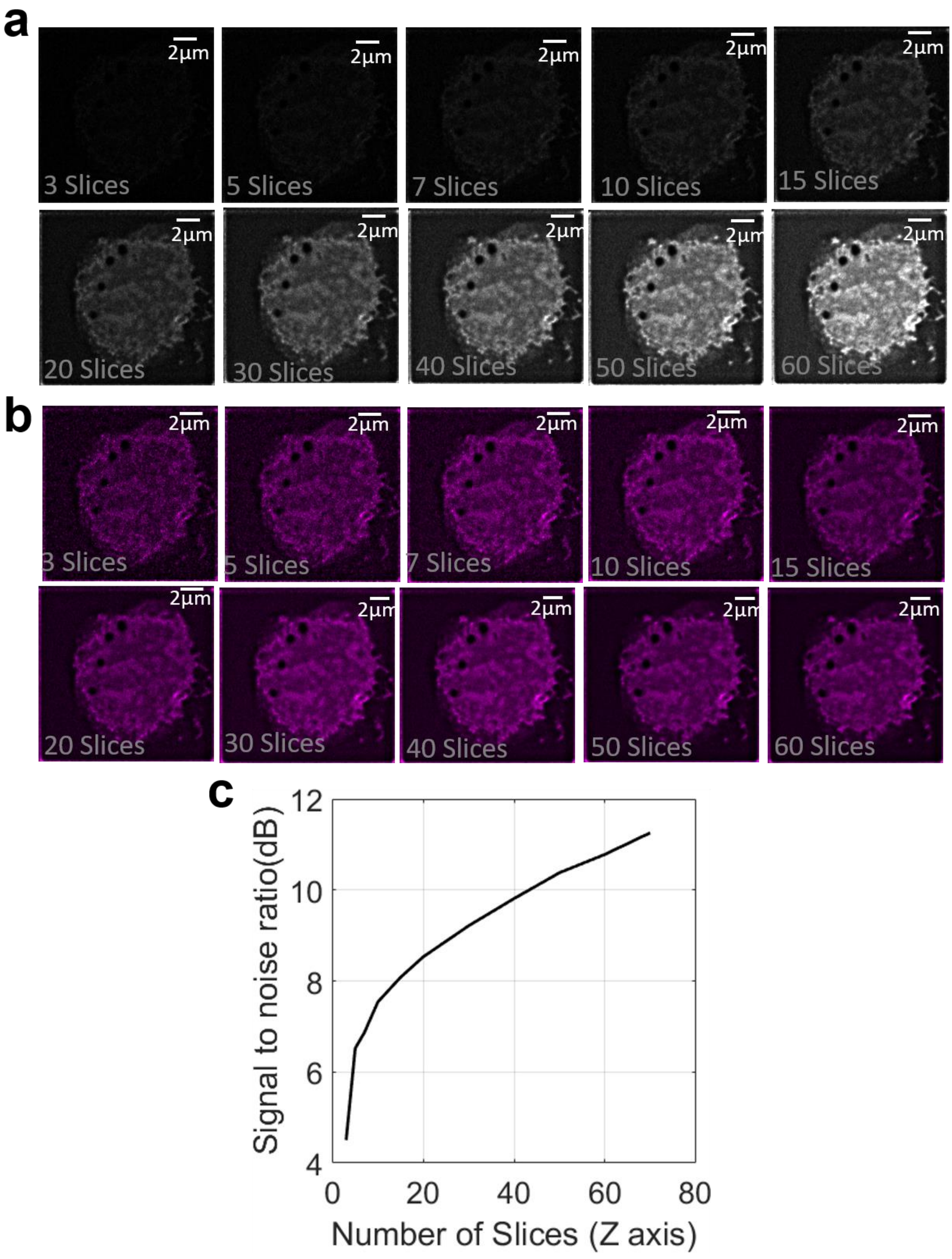
a
2µm
3 Slices
5 Slices
7 Slices
10 Slices
15 Slices
20 Slices
30 Slices
40 Slices
50 Slices
60 Slices
b
3 Slices
5 Slices
7 Slices
10 Slices
15 Slices
20 Slices
30 Slices
40 Slices
50 Slices
60 Slices
c
Signal to noise ratio(dB)
Number of Slices (Z axis)
0
20
40
60
80
4
6
8
10
12

Supplementary Figure 22. Multiple depths binning enhances the signal-to-noise ratio.

a Seventy sections from the same K562 cell were analyzed. The signal-to-noise ratio (SNR) was enhanced as slices were added.

b Separately adjusted contrast levels of ion beam images demonstrated optimal spatial details with 10 to 20 slices summed.

c The plot of SNR versus the number of slices summed indicates that there was exponential enhancement up to 15 slice sums, after which the SNR enhancement provided less improvement.

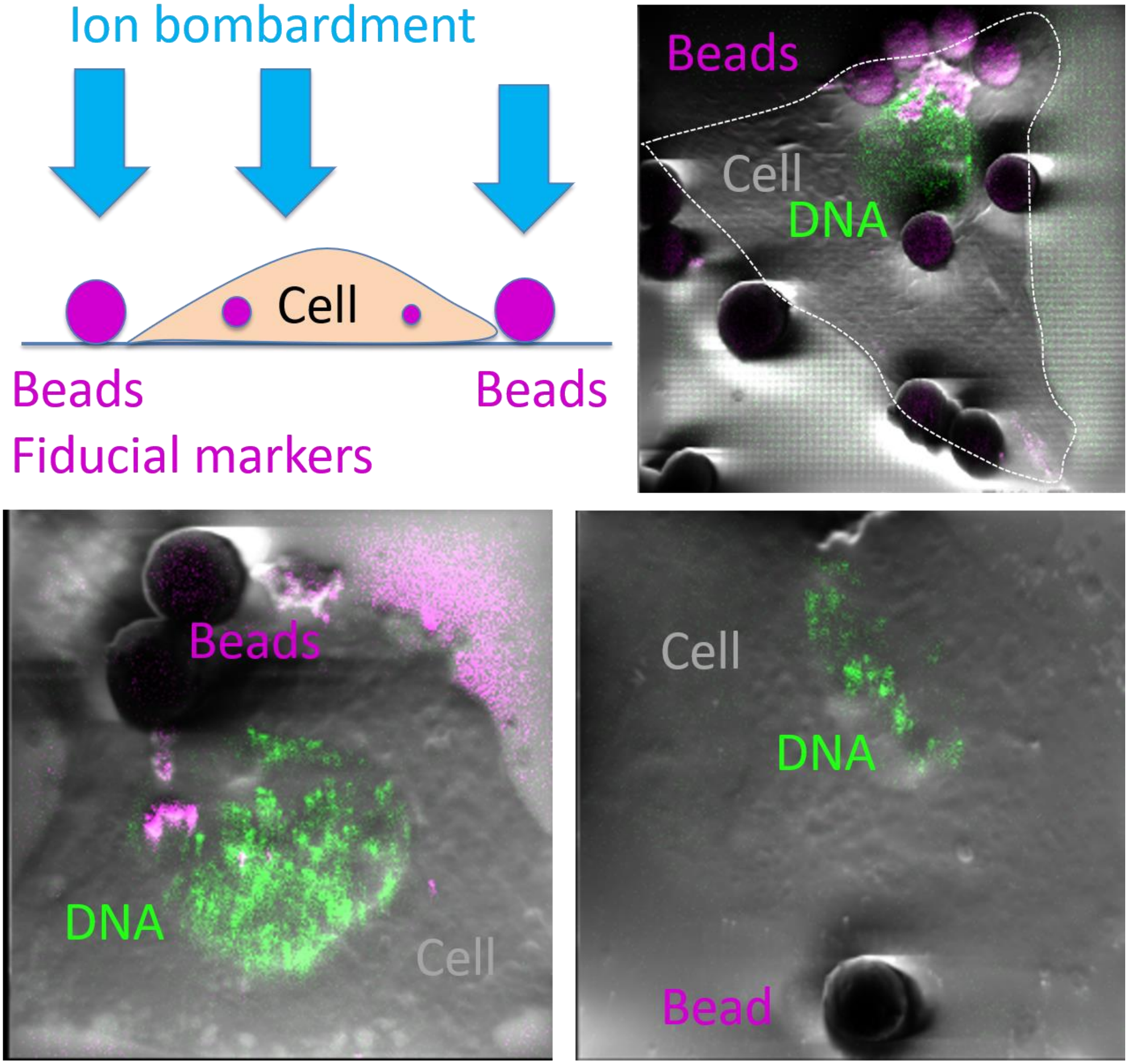
Ion bombardment
Cell
Beads
Beads
Fiducial markers
Beads
Cell
DNA
Beads
DNA
Cell
Cell
DNA
Bead

Supplementary Figure 23. Microbead addition enables correction for spatial offsets for each depth image. Beads can be used either outside the cell or inside the cell.

As the bead is made up of harder material than cellular contents, the etching of the bead requires a longer time than the etching of the cellular material. As a result of the differences in etch rates, the bead pattern looks similar across different scans.

The edge or the center of the bead is then used to correct the lateral shifts.

To reduce coagulation, beads are washed with water and ethanol before the experiments. The bead solution is then loaded onto the silicon substrate and spin-coated at 125 RCF for 5 minutes.

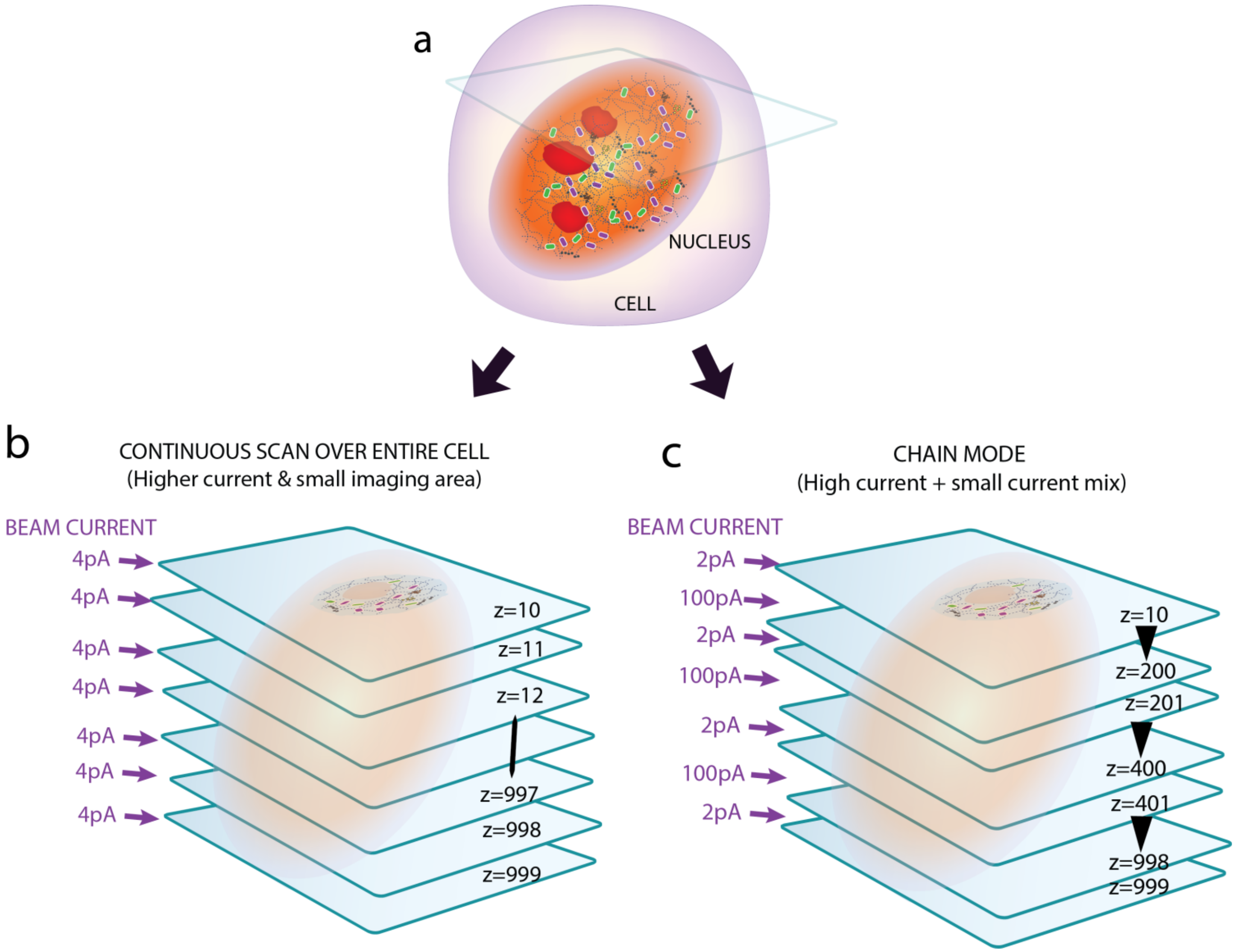
a
NUCLEUS
CELL
b
CONTINUOUS SCAN OVER ENTIRE CELL
(Higher current & small imaging area)
BEAM CURRENT
4pA
4pA
4pA
4pA
4pA
4pA
4pA
z=10
z=11
z=12
z=997
z=998
z=999
c
CHAIN MODE
(High current + small current mix)
BEAM CURRENT
2pA
100pA
2pA
100pA
2pA
100pA
2pA
z=10
z=200
z=201
z=400
z=401
z=998
z=999

Supplementary Figure 24. Alternative imaging modes are available.

a A single cell is imaged from top to bottom by ion beam tomography.

b 3D image acquisition by continuous scans using low current (in the range of 0.2 to 4 pA) only for high-resolution mapping of the entire cellular volume.

c Another ion imaging modality is the chained mode.

Depth scans were acquired by a mix of high current (100pA) and low current (e.g., 2 pA), enhancing overall IBT throughput.

Credit: Images were edited from Designua, Timonina, and Alejo Miranda/Shutterstock.com.

HeLa Cells (IdU Labeled DNA at ^{127}I Channel)

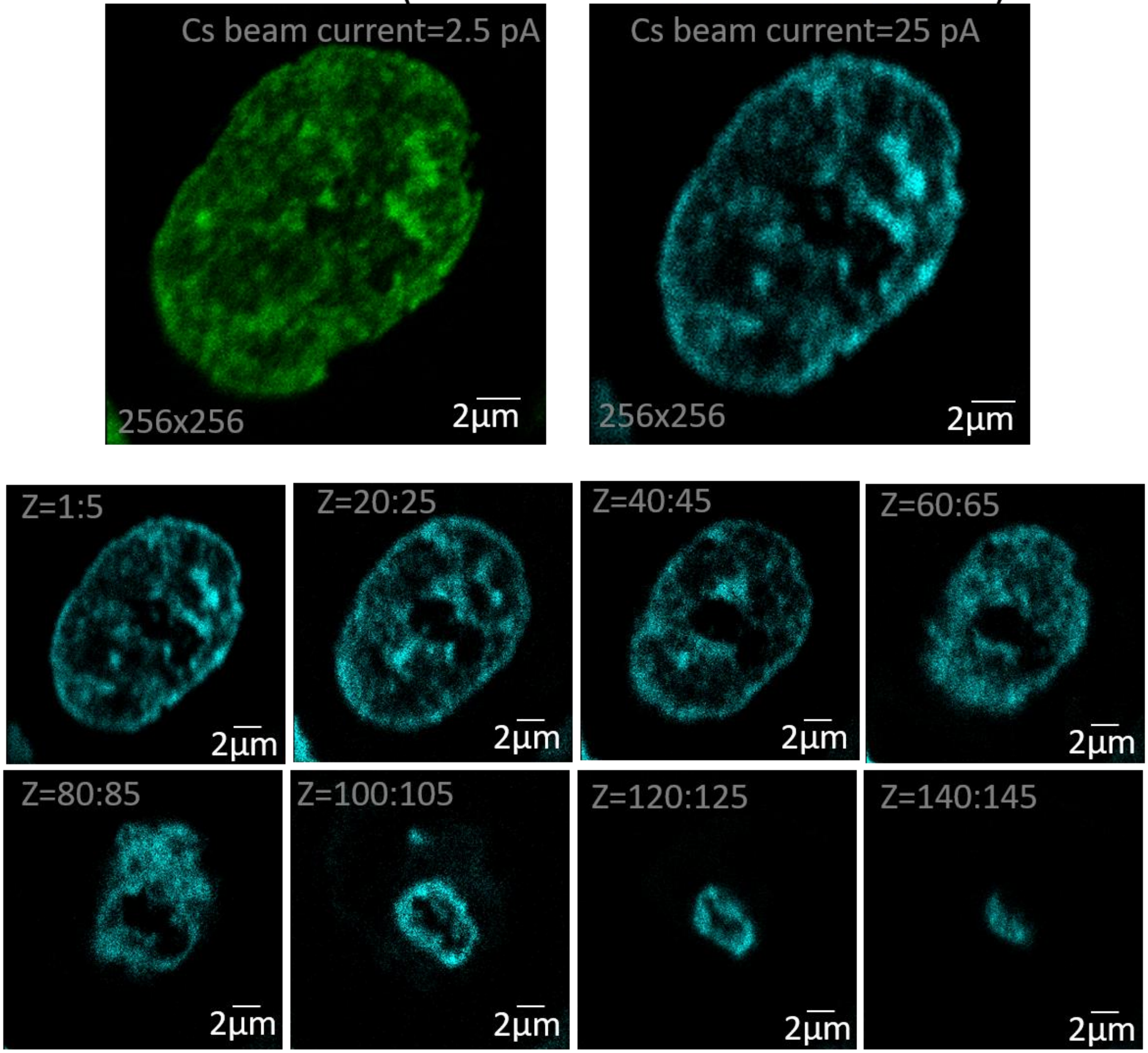

Supplementary Figure 25. Rapid depth imaging at decent spatial resolution.

HeLa cell was labeled with ^{127}I-dU for tagging DNA to be imaged by ion beams.

A higher current level (25 pA) was used to acquire the cell across 150 slices, rather than capturing up to 1,000 at 2.5 pA.

This approach reduced imaging time per cell, allowing to scan more cells in a shorter duration.

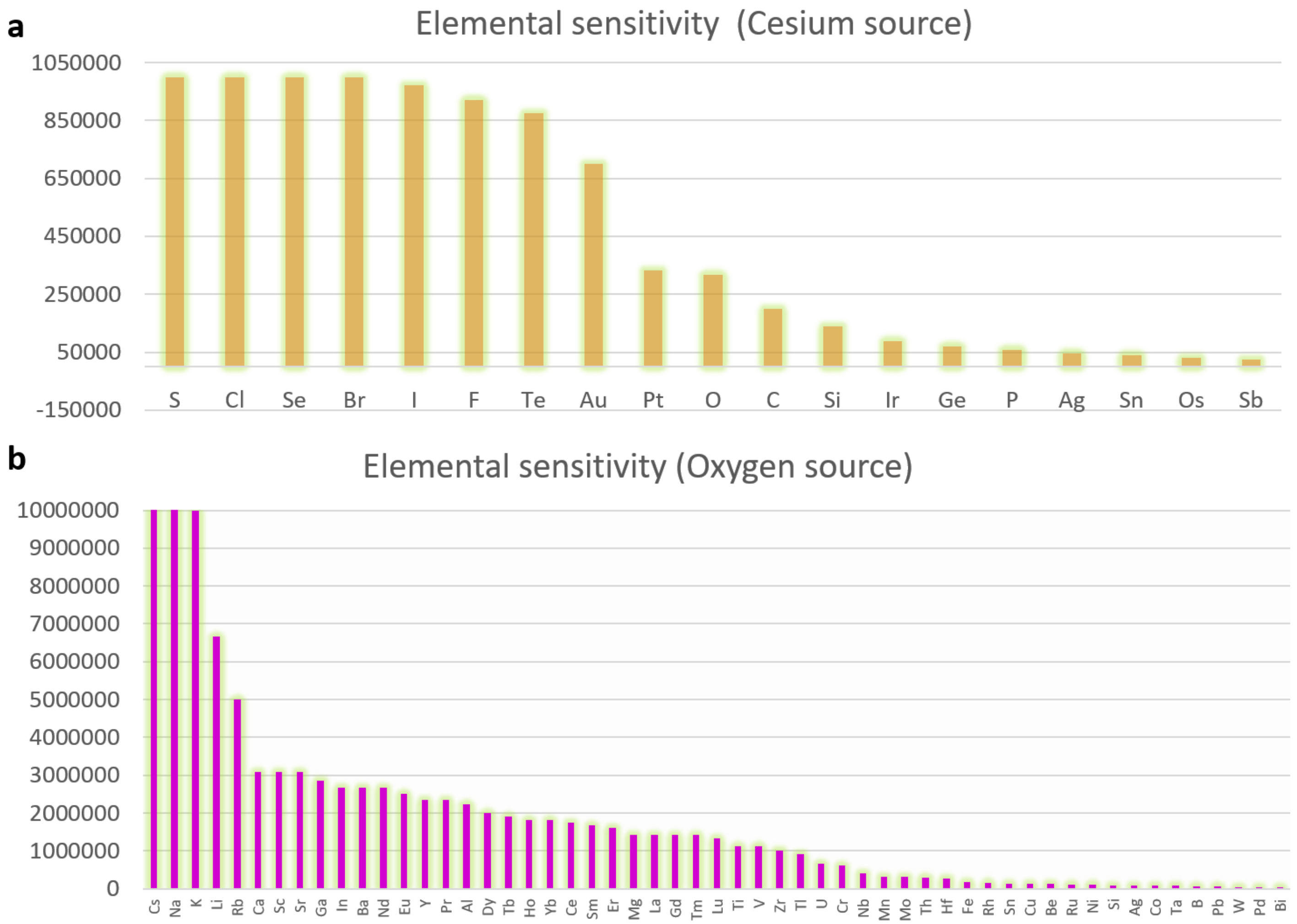

a
Elemental sensitivity (Cesium source)
1050000
850000
650000
450000
250000
50000
-150000
S Cl Se Br I F Te Au Pt O C Si Ir Ge P Ag Sn Os Sb
b
Elemental sensitivity (Oxygen source)
10000000
9000000
8000000
7000000
6000000
5000000
4000000
3000000
2000000
1000000
0
Cs Na K Li Rb Ca Sc Sr Ga In Ba Nd Eu Y Pr Al Dy Tb Ho Yb Ce Sm Er Mg La Gd Tm Lu Ti V Zr Tl U Cr Nb Mn Mo Th Hf Fe Rh Sn Cu Be Ru Ni Si Ag Co Ta B Pb W Pd Bi

Supplementary Figure 26. Elemental sensitivity analysis of ion images acquired using cesium and oxygen sources.

The raw values were extracted from a previous publication (*Wilson. SIMS quantification in Si, GaAs, and diamond – an update. International Journal of Mass spectroscopy and Ion Processes, 143, 43-49, 1995; Table 1*).

For the cesium source-based ion beam imaging, the relative sensitivity factors (RSF) of E^- RSF[b] (Negative secondary ions measured by 14.5 keV Cs+ source) were divided by the max value of the RSF (lowest sensitivity number), providing a direct measure of sensitivity.

For the oxygen source, E^+ RSF[a] (Positive secondary ions measured by 4 keV O+ source) values were also normalized by the largest number to enable sensitivity comparisons.

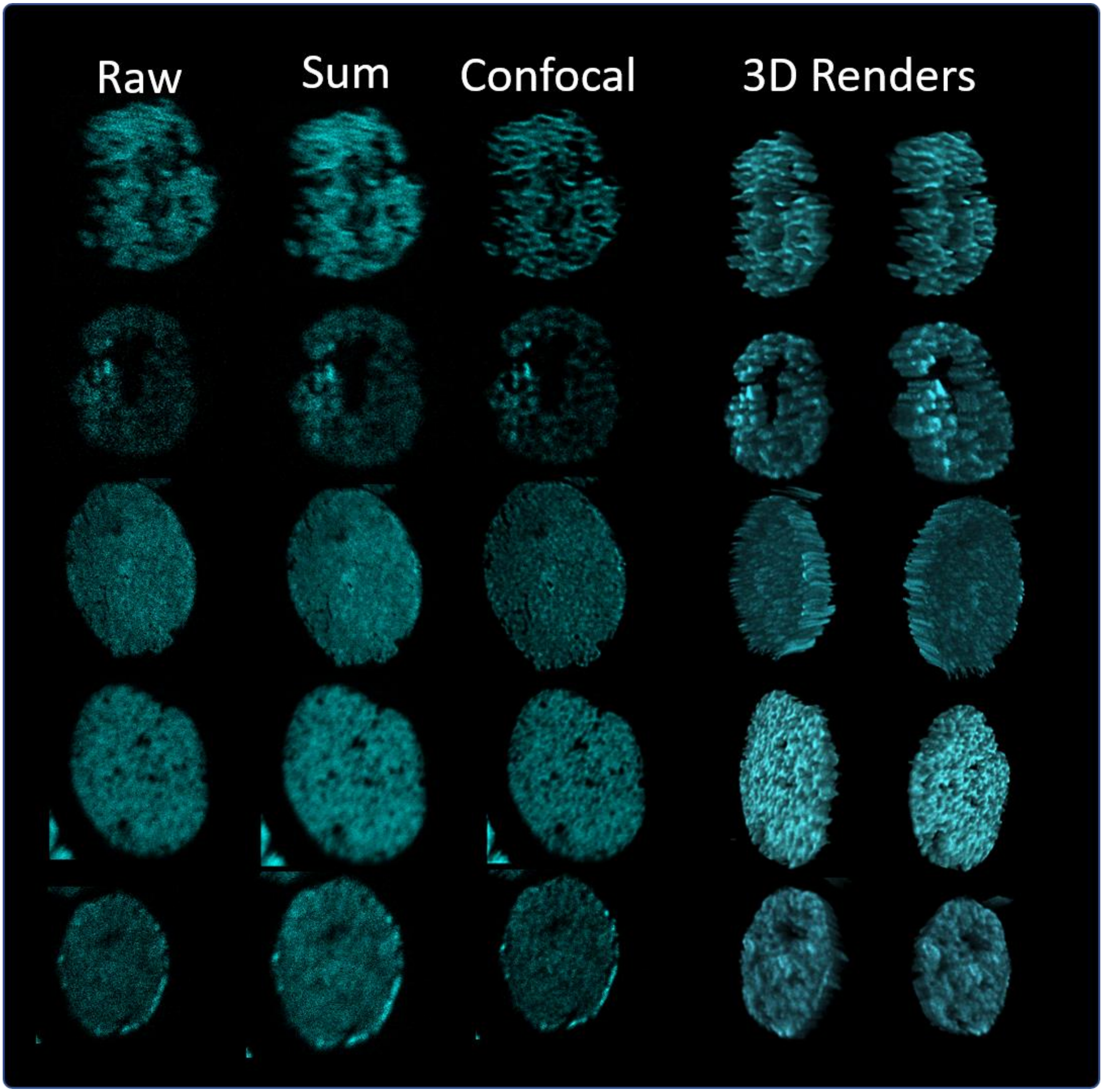
Raw
Sum
Confocal
3D Renders

Supplementary Figure 27. Deconvolution-IBT approach was validated in genomic images by ^{127}I channel using 24 hours of ^{127}I-dU labeling in Jurkat cells (top 2 rows) and HeLa cells (bottom 3 rows).

Raw: Acquired ion beam at each depth scan.

Sum: Binning of 5 subsequent depth slices.

Confocal: Digitally deconvolved images on binned sections.

3D Renders from the reconstructed ion beam tomographic slices from two different viewing angles.

The high quality of final images compared to the initial ion images demonstrated the benefit of the presented mathematical analysis pipeline.

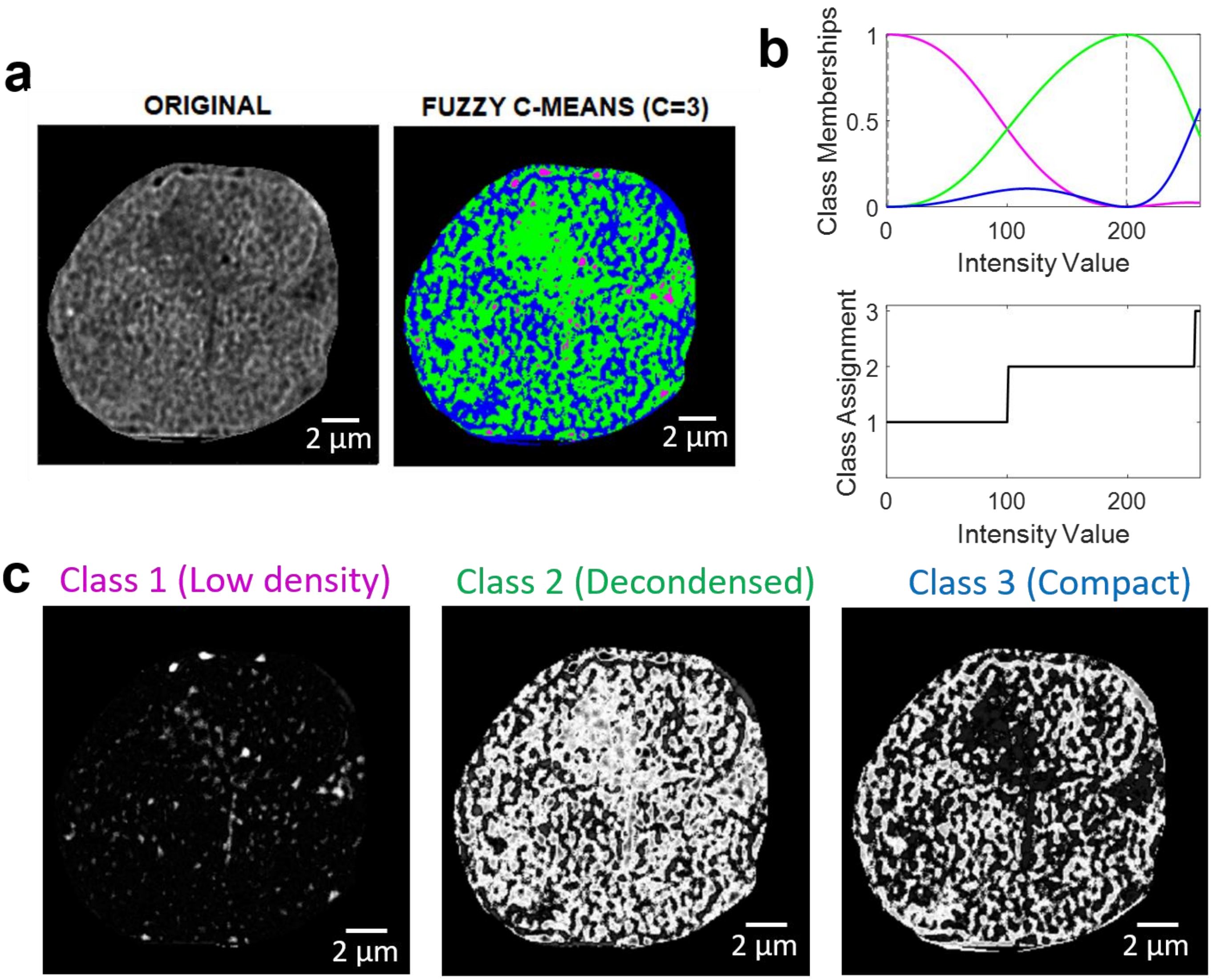
a
ORIGINAL
FUZZY C-MEANS (C=3)
2 µm
2 µm
b
Class Memberships
1
0.5
0
0
100
200
Intensity Value
Class Assignment
3
2
1
0
100
200
Intensity Value
c
Class 1 (Low density)
Class 2 (Decondensed)
Class 3 (Compact)
2 µm
2 µm
2 µm

Supplementary Figure 28. Fuzzy logic segmentation in a Nalm6, B cell lymphoblast cell, for 50th slice.

a The original image (improved by mathematical pipeline) was used for the segmentation process. A fuzzy C-means clustering (C=3) was calculated for the ^{31}P image based on intensity values.

b Pixel values were sorted into class memberships based on the density of chromatin distribution.

c Each ion-channel image corresponding to the assignments of class 1 (low chromatin density), class 2 (decondensed chromatin), and class 3 (compact chromatin).

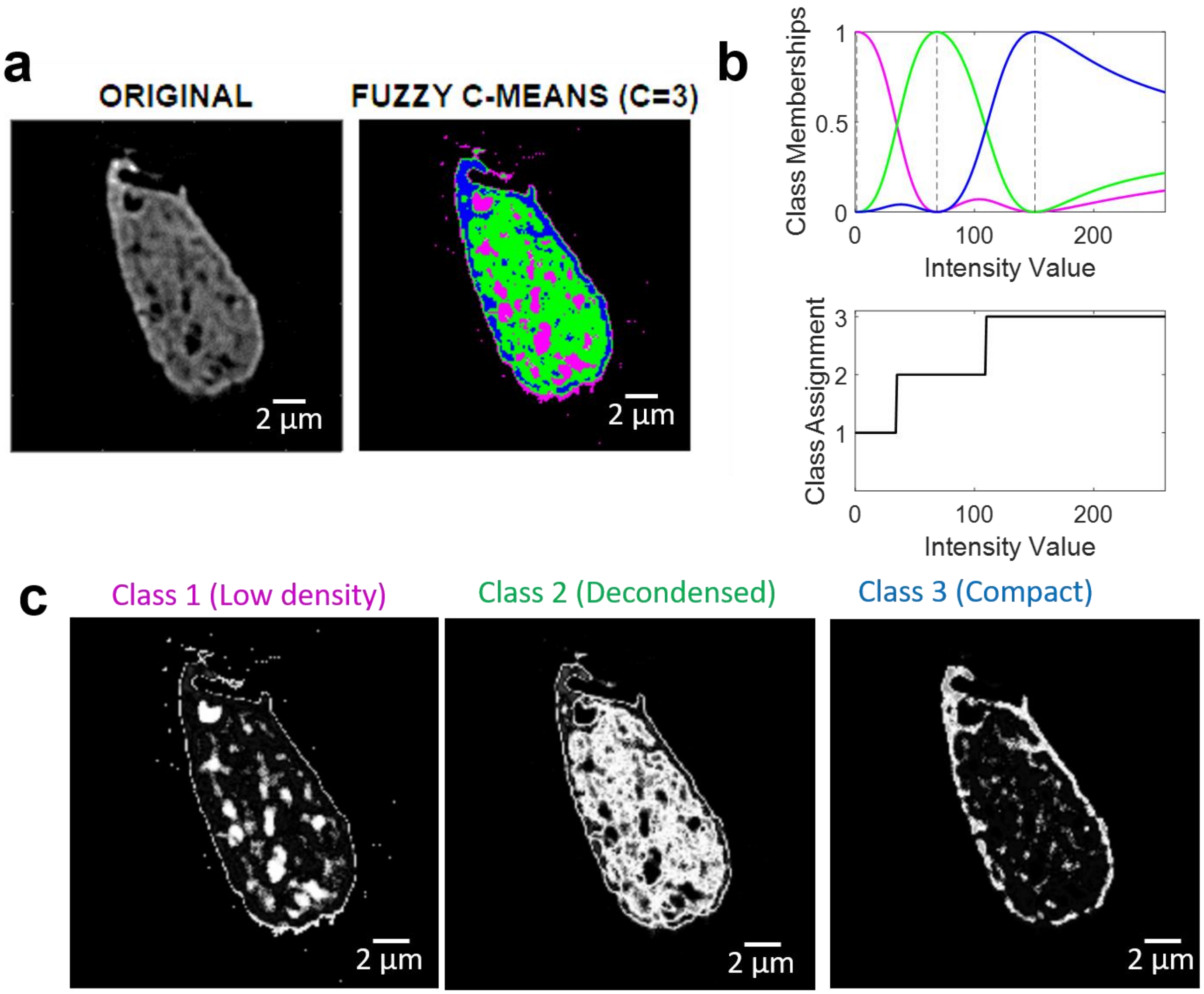
a
ORIGINAL
FUZZY C-MEANS (C=3)
2 µm
2 µm
b
Class Memberships
1
0.5
0
0
100
200
Intensity Value
Class Assignment
3
2
1
0
100
200
Intensity Value
c
Class 1 (Low density)
Class 2 (Decondensed)
Class 3 (Compact)
2 µm
2 µm
2 µm

Supplementary Figure 29. Fuzzy logic segmentation in a HeLa cell for 500th slice.

Similar to the previous **Supplementary Fig. 28**.

a C-means clustering (C=3) was used to sort the pixel values,

b class assignments for three density levels of chromatin, and

c segmented images of chromatin for classes 1-3.

Instead of using direct calculation of intensity values, Fuzzy C-means based clustering of ion images provided a determination of chromatin states without manually changing intensity threshold levels for segmentation.

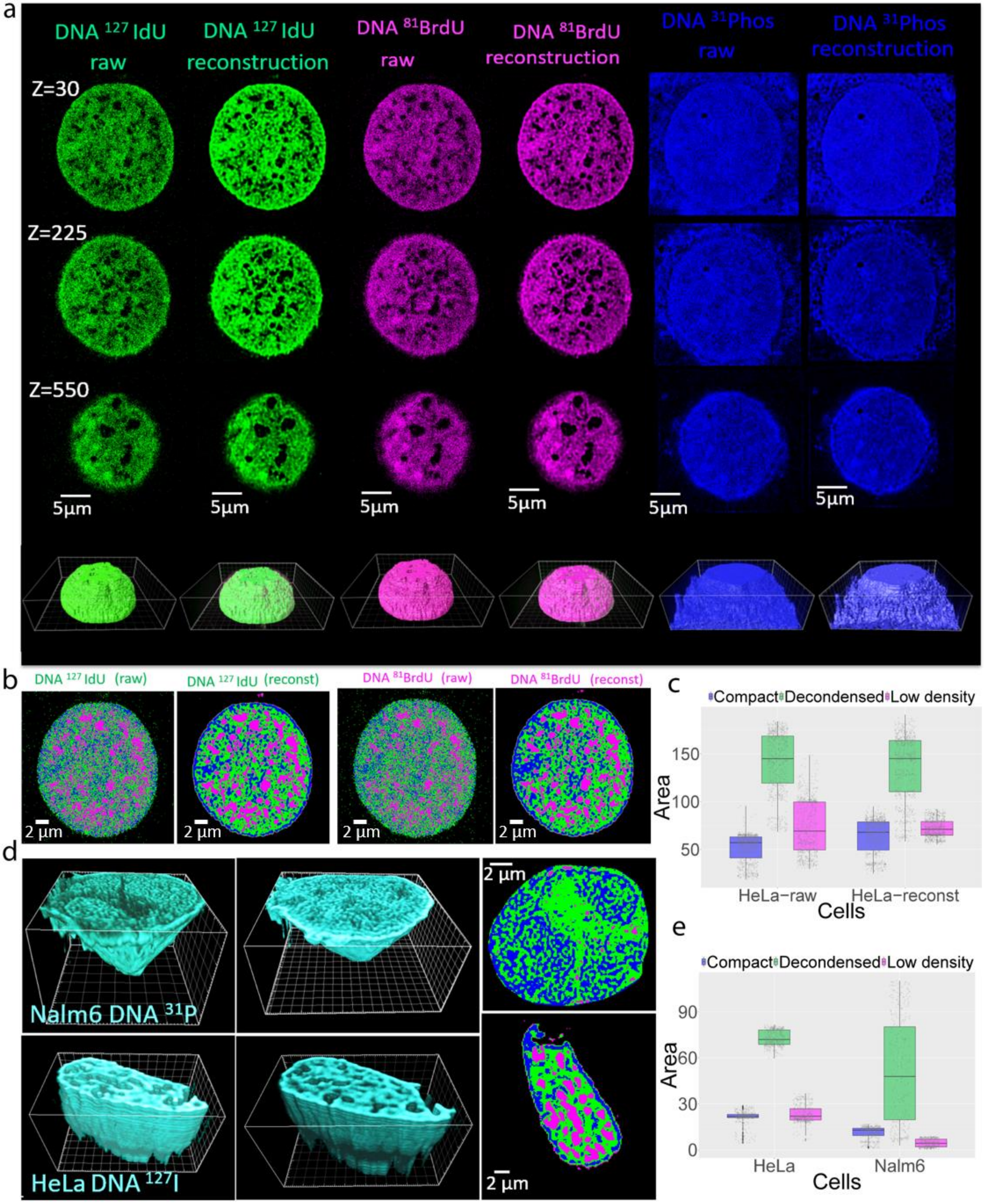

a
DNA 127 IdU raw
DNA 127 IdU reconstruction
DNA 81BrdU raw
DNA 81BrdU reconstruction
DNA 31Phos raw
DNA 31Phos reconstruction
Z=30
Z=225
Z=550
5µm
b
DNA 127 IdU (raw)
DNA 127 IdU (reconst)
DNA 81BrdU (raw)
DNA 81BrdU (reconst)
2 µm
c
Compact
Decondensed
Low density
Area
150
100
50
HeLa-raw
HeLa-reconst
Cells
d
Nalm6 DNA 31P
HeLa DNA 127I
2 µm
e
Compact
Decondensed
Low density
Area
90
60
30
0
HeLa
Nalm6
Cells

Supplementary Figure 30. Chromatin density analysis of Deconvolution-IBT and Raw-IBT images.

a Thymidine analogues ^{127}I-dU and ^{81}Br-dU were simultaneously incorporated into cells for 24 hours. This allowed chromatin visualization in a single HeLa cell in two different mass channels, providing two distinct datasets to test our mathematical pipeline. The first column shows typical raw ion images from 30th, 225th, and 550th depths from the ^{127}I-dU channel (green). At the bottom of each column, a 3D render of 700 slices is shown as an ion beam cell tomogram. The second column presents reconstructed ^{127}I-dU images; these are sharper and better reveal spatially resolved features of chromatin fibers than the raw images. The third and fourth columns correspond to raw and reconstructed images of the chromatin obtained by analysis of the ^{81}Br-dU channel (magenta). Reconstructed images in the second and fourth columns agree well with each other, validating the mathematical analysis. The raw images in the first and third columns show significant spatial differences, even though both I-dU and Br-dU label replicating DNA. The 3D renders in the bottom row show strong chromatin features only in the renders from reconstructed ion images. The renders based on the raw images show random fluctuations due to the noise levels of each ion beam image. The fifth and sixth columns show the ^{31}P channel, which is dominated by signals due to phosphate in the DNA backbone of the nucleus. As proteins and RNAs in both cytosol and nucleus contribute to this signal there are differences compared to the ^{127}I-dU and ^{81}Br-dU channel images.

b To explore local regions of chromatin, image segmentation was performed on the raw and reconstructed images of the ^{127}I-dU and ^{81}Br-dU images. A 225th depth image was partitioned into three colors based on the density of the chromatin using a fuzzy logic segmentation algorithm: compact chromatin (blue), decondensed chromatin (green), and low-density chromatin (magenta). Raw images in both channels failed to resolve fibers, whereas the three colors are present in similar spatial patterns in ^{127}I-dU and ^{81}Br-dU reconstructed images. Each data point corresponds to a single ion beam depth slice from top to bottom of a single cell (n=1,000).

c A box plot showing area occupied by chromatin forms in raw and reconstructed images, wherein median, first and third quartile, and 95% confidence interval of the median are shown. n = 542 slices over a long IBT scan for each chromatin state were included in the analysis.

d Upper images: Chromatin features in a Nalm6 cell imaged using the ^{31}P channel. Lower images: HeLa cell imaged using the ^{127}I-dU channel, followed by reconstructions and segmentation. The first and second columns show cross-sections of the chromatin at the 500th slice and the 400th slice, respectively. Segmented images of ^{31}P in the Nalm6 cell exhibits minimal low-density chromatin signal that might be due to other background signals from proteins and RNA, whereas segmentation in ^{127}I provided all three partitions of the chromatin in the HeLa cell.

e Box plot of areas occupied by chromatin forms across about 350 sections (each data point represents a single section) in these the Nalm6 cell imaged in the ^{31}P channel and the HeLa cell imaged using the ^{127}I channel. Cell-type differences and two different channels contribute to the heterogeneity in the compact, decondensed, and low-density regions. Box plot indicates the median, first and third quartile, and 95% confidence interval of the median. n = 542 slices over a long IBT scan for each chromatin state were included in the analysis.

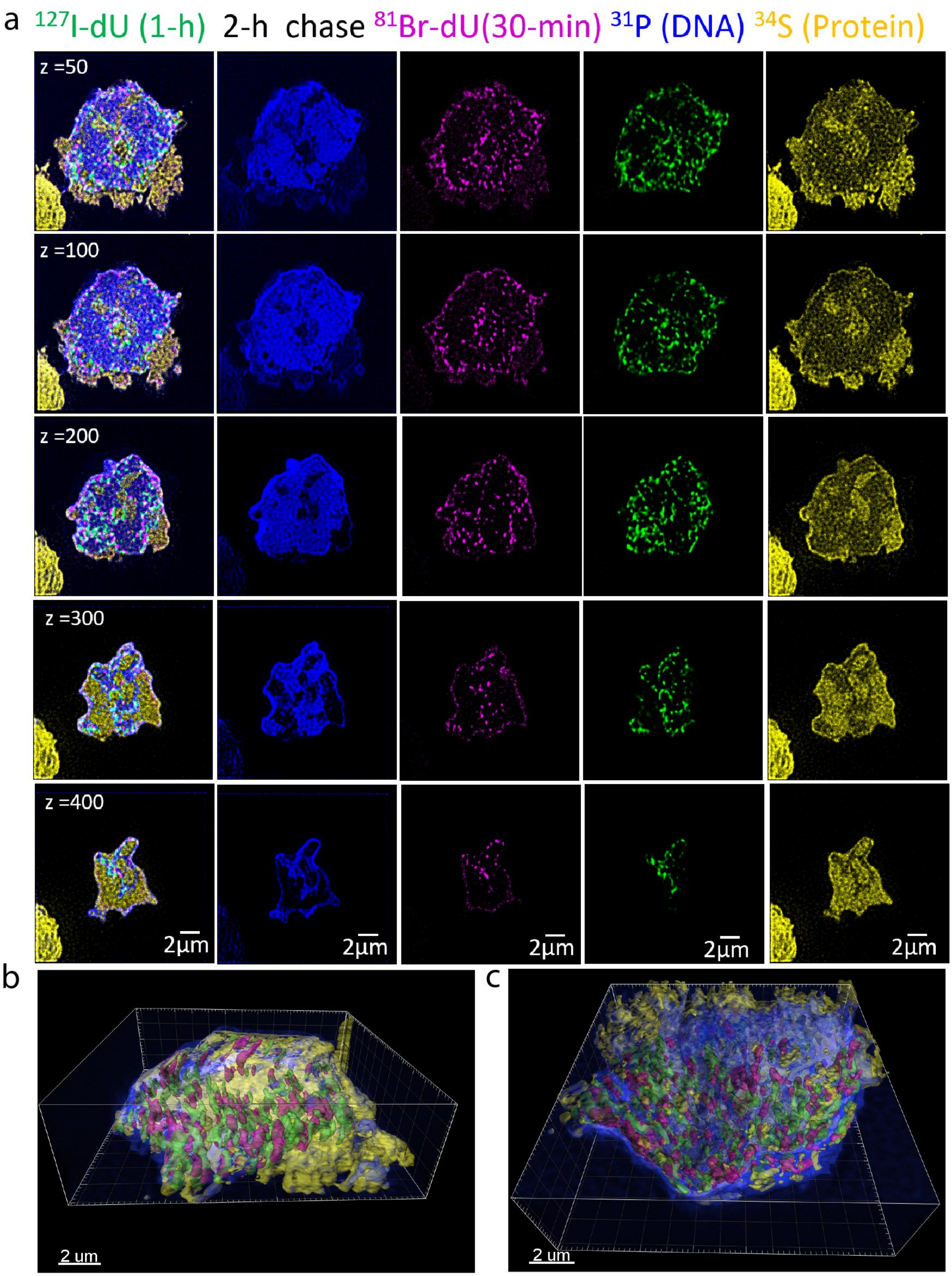
a
127I-dU (1-h) 2-h chase 81Br-dU(30-min) 31P (DNA) 34S (Protein)
z =50
z =100
z =200
z =300
z =400
2μm
b
c
2 um

Supplementary Figure 31. Four-channel representation of replication data in Figure 4 (2-h chase).

a Nalm6 cell was pulsed with ^{127}I-dU (green) for 1 hour and then chased in culture media for 2 hours, followed by a second pulse with ^{81}Br-dU (magenta) for 30 minutes. IBT image sections for 50th, 100th, 200th, 300th, and 400th slices were shown. ^{31}P (blue) was used for chromatin analysis. ^{34}S (yellow) was utilized as an indicator of protein concentration.

b 3D visualization of 600 slices in a Nalm6 cell. Replication sites (green and magenta), chromatin (blue), and proteins (yellow). A higher concentration of replication forks was around the half bottom of the cell.

c An inverted 3D renders of the same Nalm6 cell with four colors as denoted.

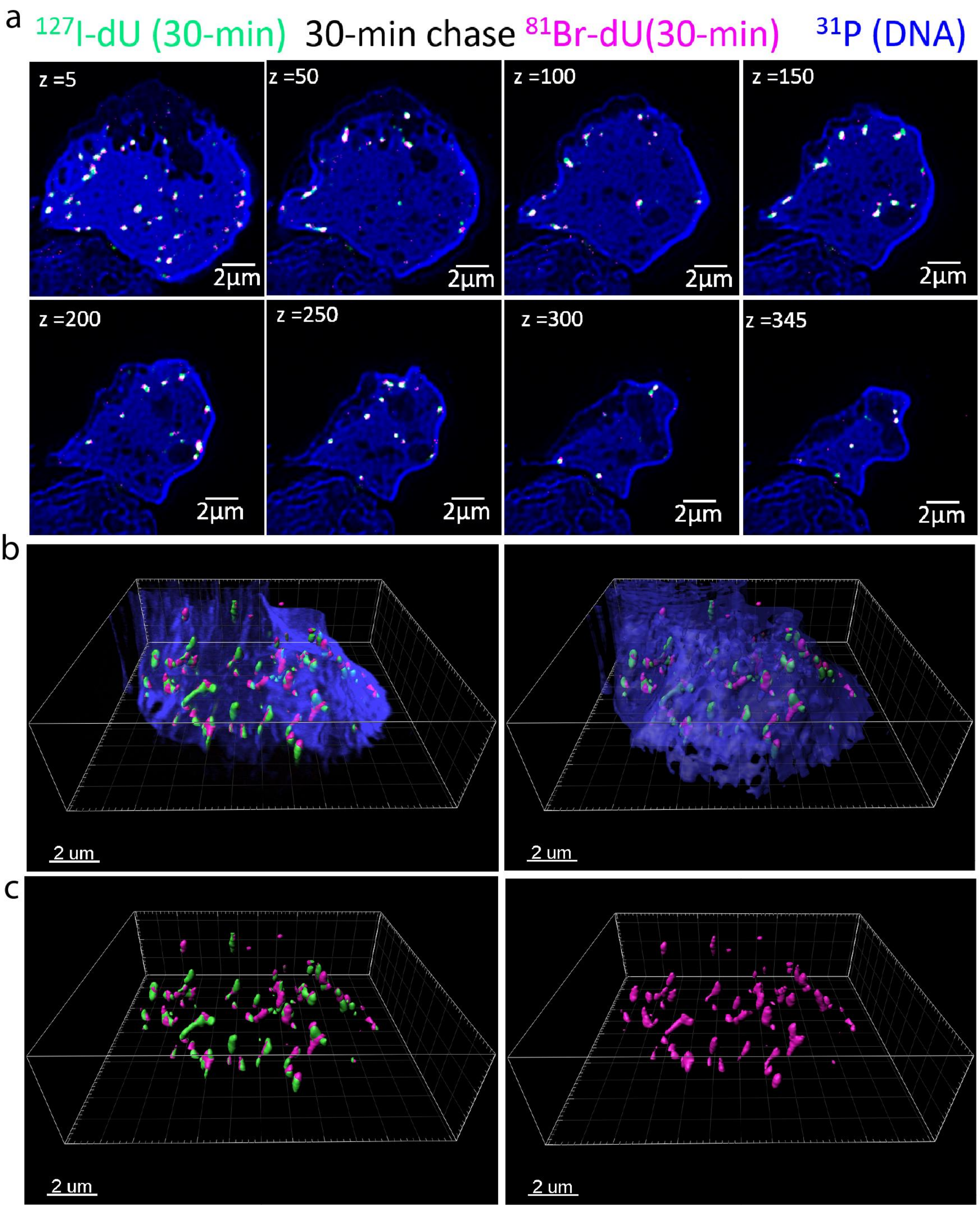
a
127I-dU (30-min) 30-min chase 81Br-dU(30-min) 31P (DNA)
z =5
z =50
z =100
z =150
z =200
z =250
z =300
z =345
2μm
b
2 um
c
2 um

Supplementary Figure 32. Biological replicate experiment for replication dynamics in **Figure 4** (30-min chase).

a Nalm6 cell was pulsed with ^{127}I-dU (green) for 30 minutes and then chased in culture media for 30 minutes, followed by a second pulse with ^{81}Br-dU (magenta) for 30 minutes. Digitally processed IBT image sections for 5th, 50th, 100th, 150th, 200th, 250th, 300th, and 345th slices were shown. ^{31}P (blue) was used to identify largely chromatin.

b 3D renders of 350 sections in the Nalm6 cell. The surface function was used to colorize replication site images. The 31P channel was original data (left) and surface renders with 50% transparency settings in the 3D render.

c Only replication forks (green and red) were highlighted in 3D, showing significant overlaps and variability in the spatial distribution in cellular volumes.

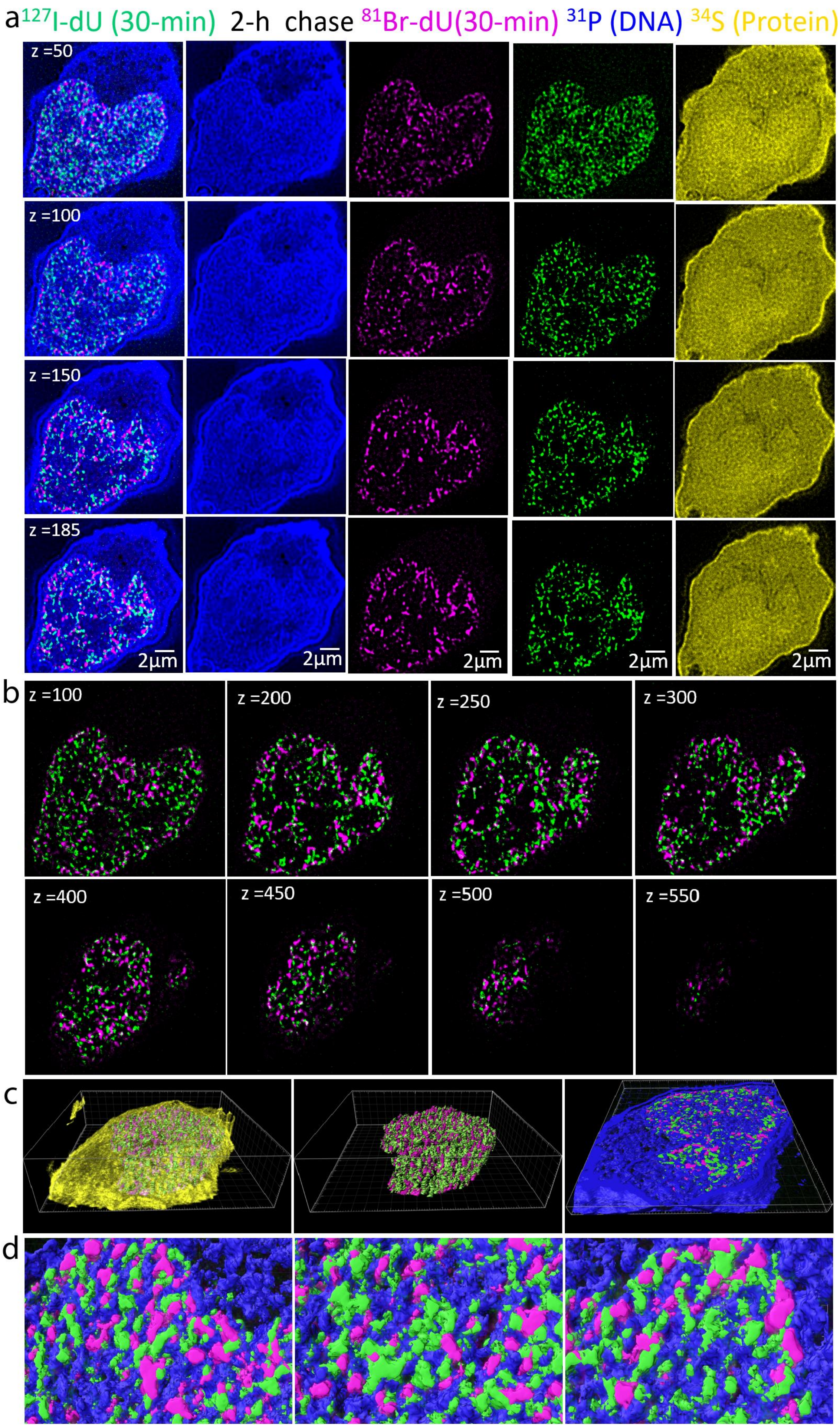

a
127I-dU (30-min) 2-h chase 81Br-dU(30-min) 31P (DNA) 34S (Protein)
z =50
z =100
z =150
z =185
2μm
2μm
2μm
2μm
2μm
b
z =100
z =200
z =250
z =300
z =400
z =450
z =500
z =550
c
d

Supplementary Figure 33. Biological repeat for replication dynamics in **Figure 4** (2-h chase).

a Nalm6 cell was pulsed with ^{127}I-dU (green) for 30-min and then chased in culture media for 2 hours, followed by a second pulse with ^{81}Br-dU (magenta) for 30 minutes. IBT image sections for 50th, 100th, 180th, and 185th slices were shown. ^{31}P (blue) was used for chromatin and ^{34}S (yellow) was imaged for proteins.

b Overlaps of replication forks are minimal. 2D sections from 100th, 200th, 250th, 300th, 400th, 500th, and 550th ion images.

c 3D renders of 600 slices in a Nalm6 cell. Replication sites (green and magenta), proteins (yellow), and chromatin (blue).

d Zoomed images from chromatin and replication forks, extracted from a 3D render with 185 slices.

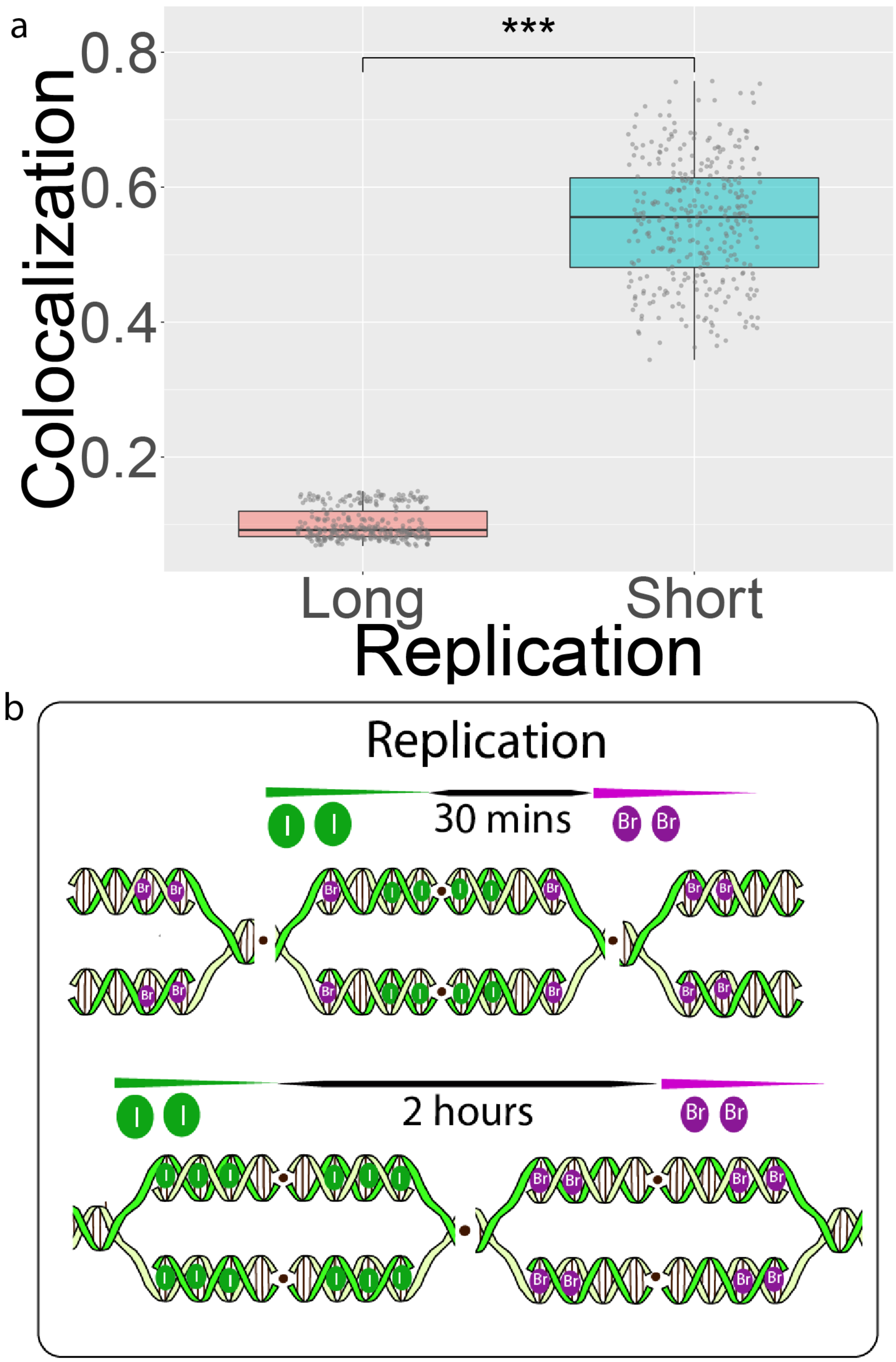
a
***
Colocalization
0.8
0.6
0.4
0.2
Long
Short
Replication
b
Replication
30 mins
I
Br
2 hours

Supplementary Figure 34. Spatial overlaps of replication forks were higher in short chase experiments.

a The correlation coefficient (CC) of 30-min chase experiments and the 2-h chase experiment was computed. Long chase provided 0.55 CC and short chase exhibited 0.09 CC values, demonstrating significantly more overlaps in 30-min compared to the 2-h chase. Each dot corresponds to a single depth slice from the ion beam tomographic data. Box plot indicates the median, first and third quartile, and 95% confidence interval of the median. The difference in the box plots had a significant p-value < 2.2e-16 *(***)* by the Wilcox test (Two-sided). *n* = 350 slices over a long IBT scan for each chase condition were included in the analysis.

b Schematic model of spatial overlaps in replication dynamics. The first pulse (Green, ^{127}I-dU) and second pulse (^{81}Br-dU) occurs in similar replication loci when the time difference between the two is shorter.

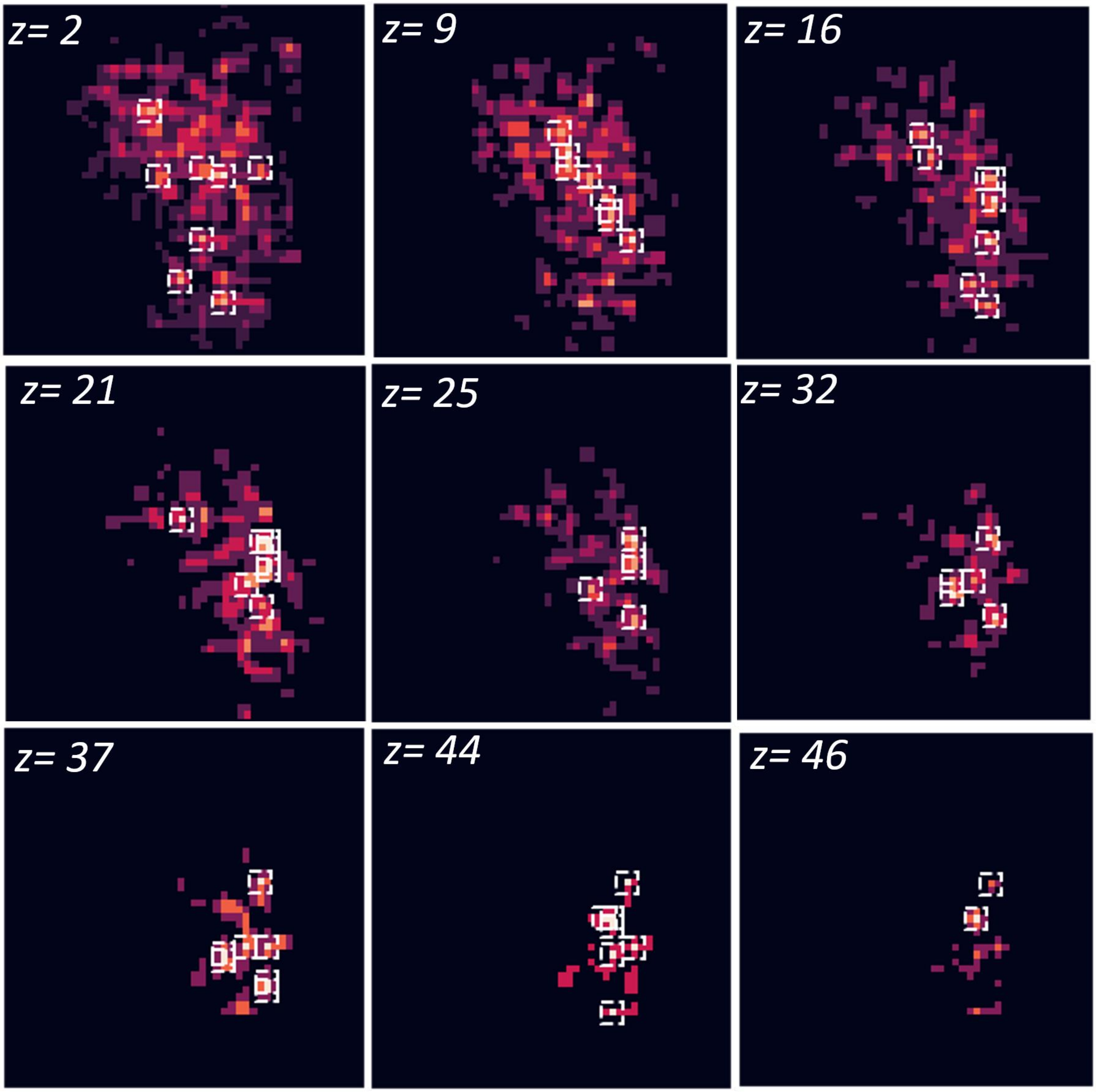
z= 2
z= 9
z= 16
z= 21
z= 25
z= 32
z= 37
z= 44
z= 46

Supplementary Figure 35. Peak detection of replication domains in ^{127}I-dU replication DNA sites.

Dashed squares show detected peaks, corresponding to brightest pixels over the threshold levels, which were overlaid by the original SILM-IBT images that are shown in the hot colormap.

This single replication site is from Figure 4b (30-min chase).

Randomly selected slices from a series of fifty IBT-slices were included in this analysis to visualize up to 10 individual replication domains.

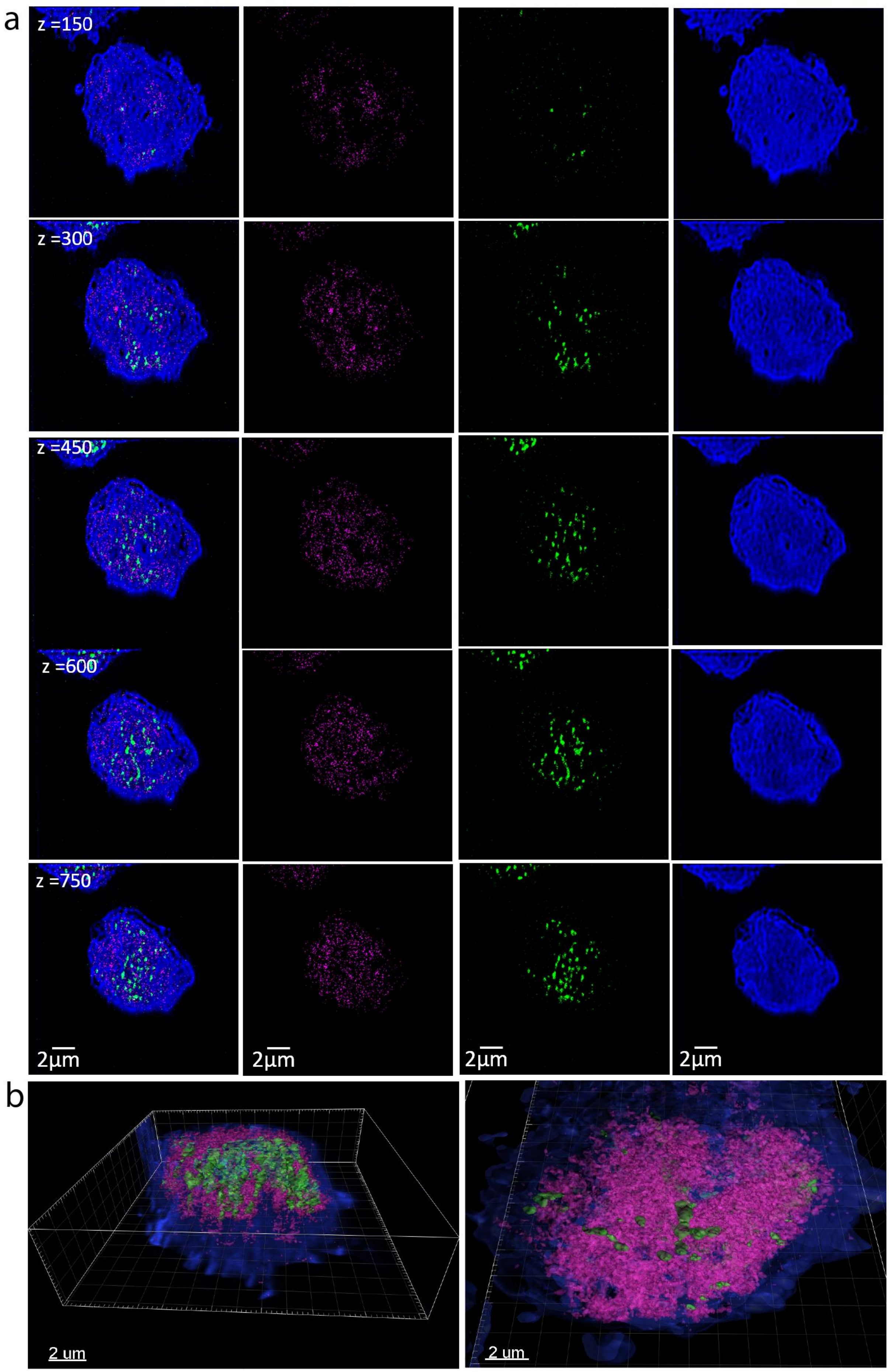
a
z =150
z =300
z =450
z =600
z =750
2μm
2μm
2μm
2μm
b
2 um
2 um

Supplementary Figure 36. Biological replicate data for nascent transcripts and replication (30-min pulse).

a Nalm6 cell was simultaneously incubated with ^{127}I-dU (green) to visualize replication and ^{81}Br-rU (magenta) to monitor transcription for 30 minutes. Tomographic ion slices for 150th, 300th, 450th, 600th, and 750th depths were presented. ^{31}P (blue) showed folding chromatin patterns.

b 3D renders of 750 slices for the Nalm6 cell. Replication sites (green), nascent transcripts (magenta), and chromatin (blue). Spatial isolation of RNA transcripts and replication forks was detected.

Note that spatial regions that look slightly darker in the rightest column (chromatin) exhibited higher transcription, yielding a direct visualization of the globally decondensed chromatin landscape.

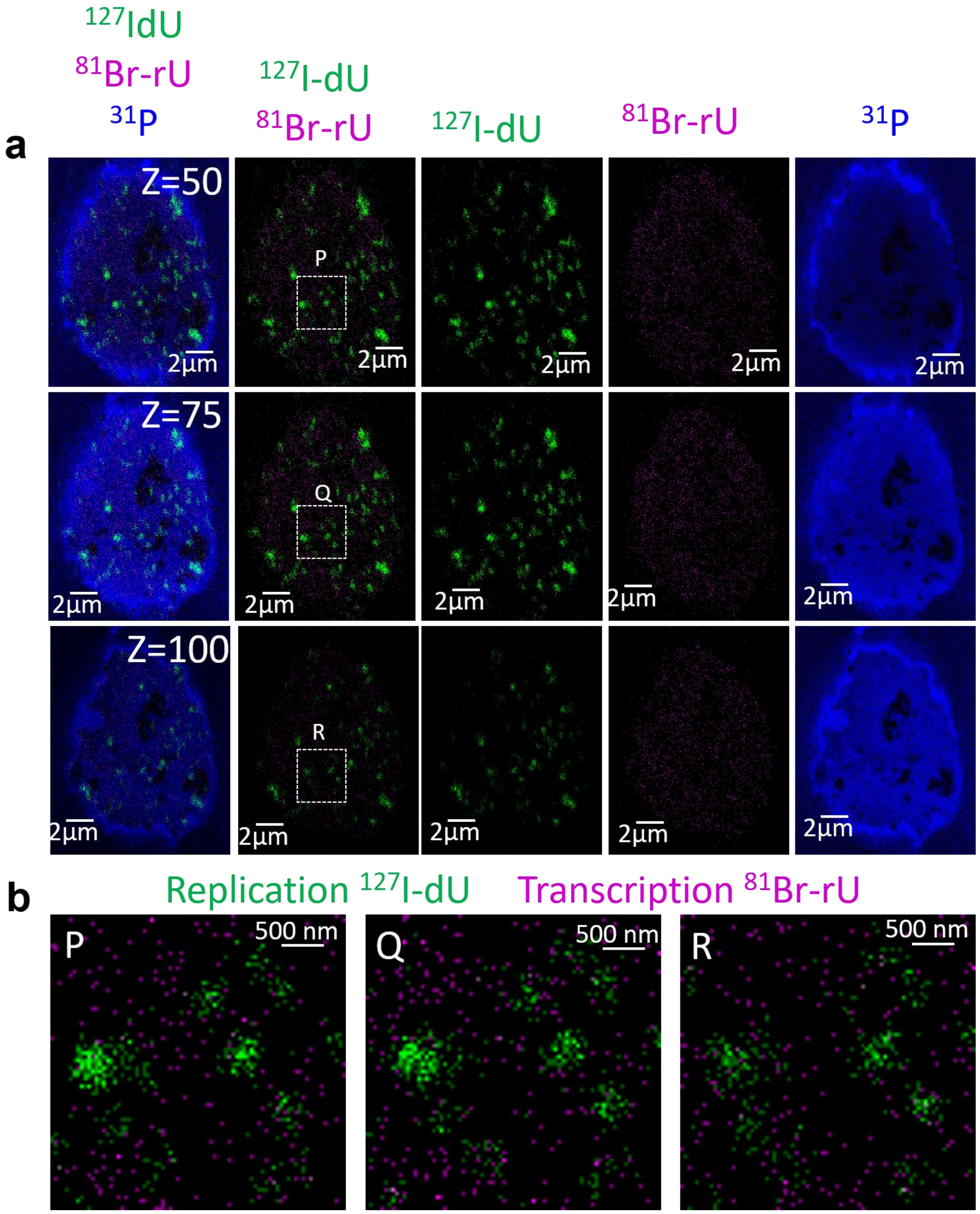

a
^{127}IdU
^{81}Br-rU
^{31}P
^{127}I-dU
^{81}Br-rU
^{127}I-dU
^{81}Br-rU
^{31}P
Z=50
Z=75
Z=100
P
Q
R
2µm
b
Replication ^{127}I-dU
Transcription ^{81}Br-rU
P
Q
R
500 nm

Supplementary Figure 37. Another replicate for the simultaneous analysis of transcription and replication by IBT.

a Nalm6 cells were incubated with ^{127}I-dU and ^{81}Br-rU for 30 minutes in culture. Ion images for each channel were presented in each row for 50th, 75th, and 100th slices.

b Pixels from two distinct images for newly synthesized RNAs (magenta) and DNAs (green) do not overlap in the zoomed regions (P, Q, and R), demonstrating spatial segregation of transcription and replication.

Note that these images were not processed by the mathematical pipeline, rather raw images are presented. 512x512 scans were used at 2 pA imaging current.

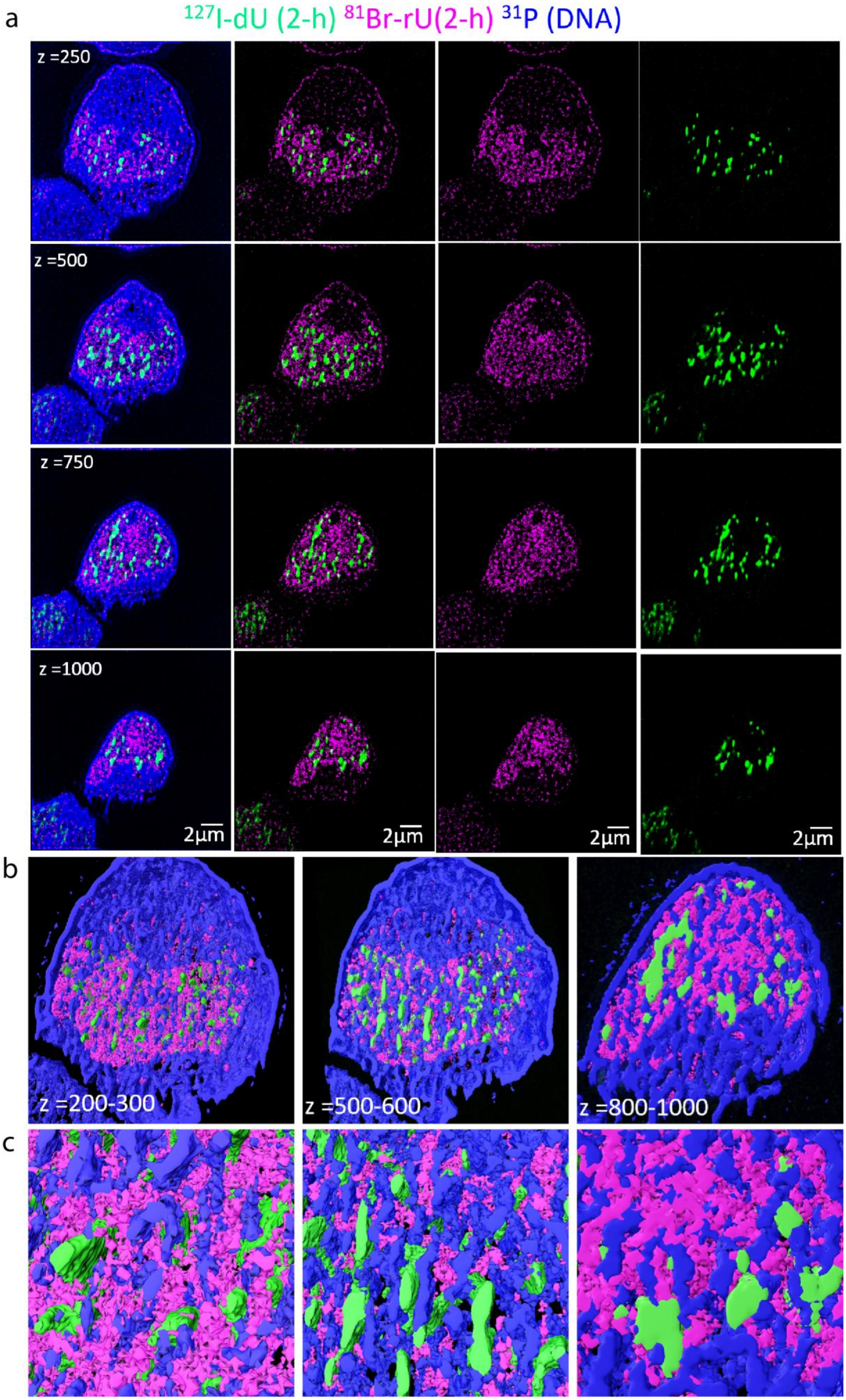
a
^{127}I-dU (2-h) ^{81}Br-rU(2-h) ^{31}P (DNA)
z =250
z =500
z =750
z =1000
2µm
2µm
2µm
2µm
b
z =200-300
z =500-600
z =800-1000
c

Supplementary Figure 38. Visualization of transcription and replication after 2-h pulse with ^{127}I-dU and ^{81}Br-rU.

a Digitally processed images for IBT at 250th, 500th, 750th, and 1000th slices. Chromatin was detected at ^{31}P (blue), replication forks by ^{127}I-dU (green), and nascent transcripts by ^{81}Br-rU channel (magenta). Long incubation time led to longer replication sites and higher transcription signals. Spatial isolation of replication and transcription was preserved even at this long pulse experiment.

b Partial 3D renders from more than 100 slices: 200-300, 500-600, and 800-1000 depths. Chromatin patterns and the spatial distribution of newly synthesized RNA and DNA around original chromatin were visualized.

c Zoomed images from the previous 3D render. Fine features of replication and transcription were detected. The spatial segregation model is further validated in these images from 2-h incorporation of metabolic labeling.

a

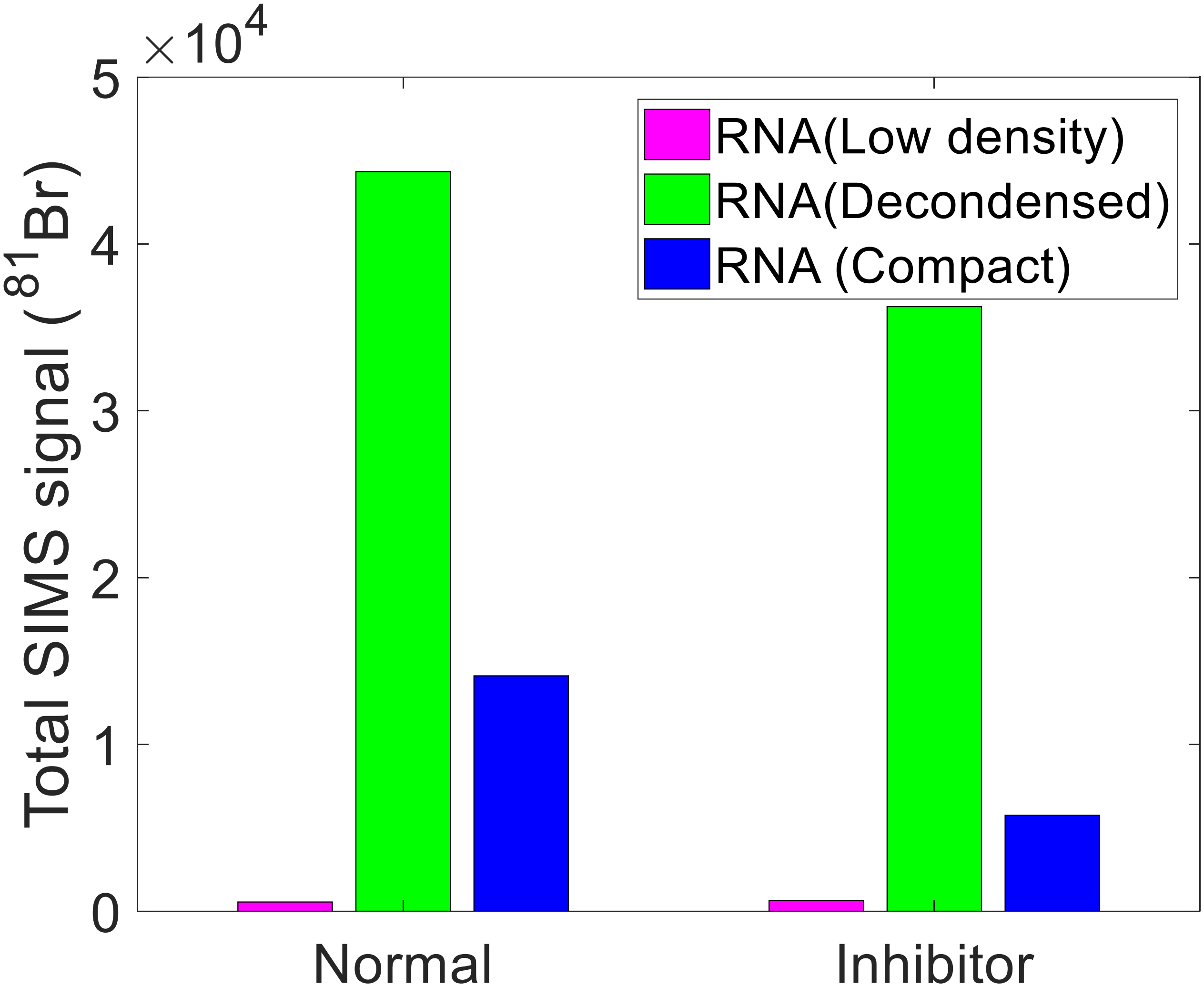

×10^4
5
4
3
2
1
0
Total SIMS signal (81Br)
RNA(Low density)
RNA(Decondensed)
RNA (Compact)
Normal
Inhibitor

b

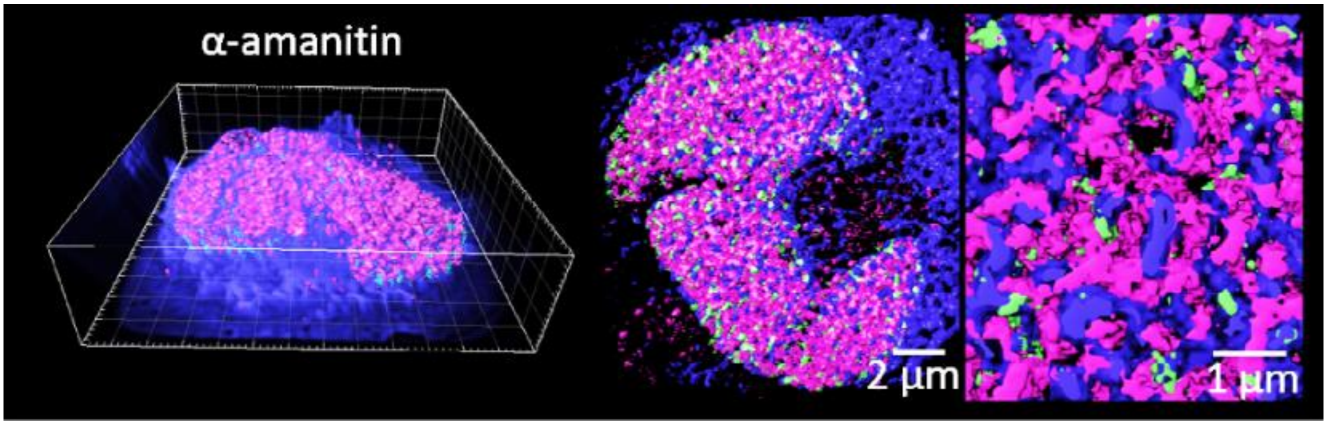

α-amanitin
2 μm
1 μm

Supplementary Figure 39. Newly synthesized RNAs are enriched in the decondensed region of the chromatin. 30 minutes of ^{81}Br-rU and ^{127}I-dU incorporations into Nalm6 cells.

a Nascent RNA distribution in the 15th section of the first Nalm6 cell (Normal) showed a high concentration in the decondensed region. Lower RNA concentration was in the compact and low-density chromatin regions.

b In the existence of α-amanitin drug for 30 minutes after the RNA labeling in the second Nalm6 cell (inhibitor), newly synthesized RNA distribution was reduced in the decondensed as well as low-density chromatin regions. Nascent RNA transcripts were partially repressed due to the degradation.

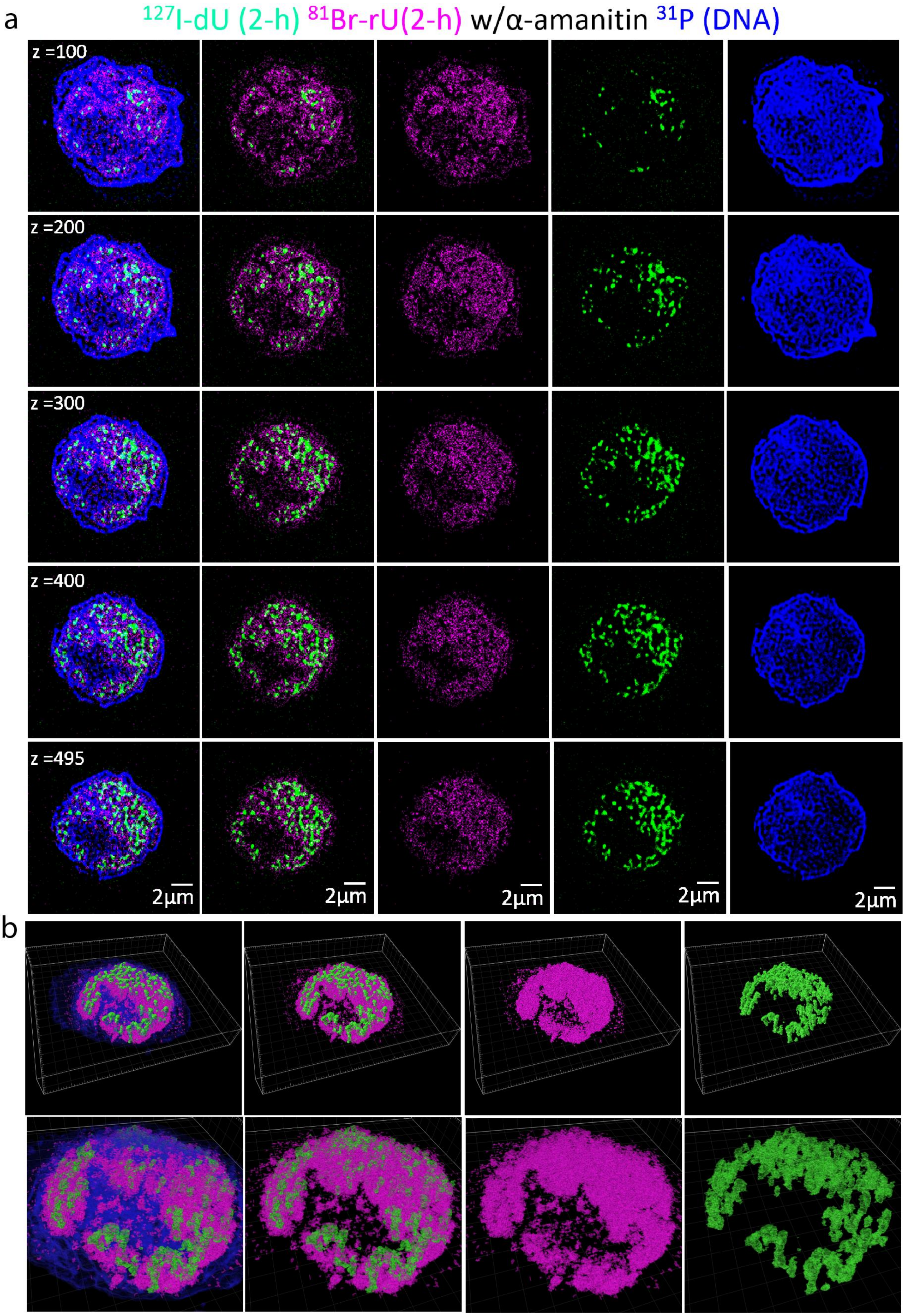
a
127I-dU (2-h) 81Br-rU(2-h) w/α-amanitin 31P (DNA)
z =100
z =200
z =300
z =400
z =495
2μm
2μm
2μm
2μm
2μm
b

Supplementary Figure 40. Transcription inhibition during the labeling by α-amanitin for 2 hours at 10-μM.

a Visualization of transcription (magenta) by ^{81}Br-rU, replication (green) by ^{127}I-dU, and chromatin (blue) by ^{31}P signal. Processed ion images from 100th, 200th, 300th, 400th, and 495th slices were presented.

b 3D Renders of transcription and replication around chromatin at a viewing angle. The top row corresponds to solid surfaces and the bottom row shows partially transparent surfaces in the IBT images.

Spatial distribution of replication and distribution was consistent with previous results:

- Isolation of transcribing DNA and replicating DNA in 3D.
- Enrichment of transcription in decondensed chromatin regions.

The effect of α-amanitin in active transcription:

- The total transcription signal was reduced compared to **Supplementary Fig. 36** (same conditions, 2-h).
- Partial repression but not total transcription block.
- Replication did not get affected by transcription inhibitors.

a

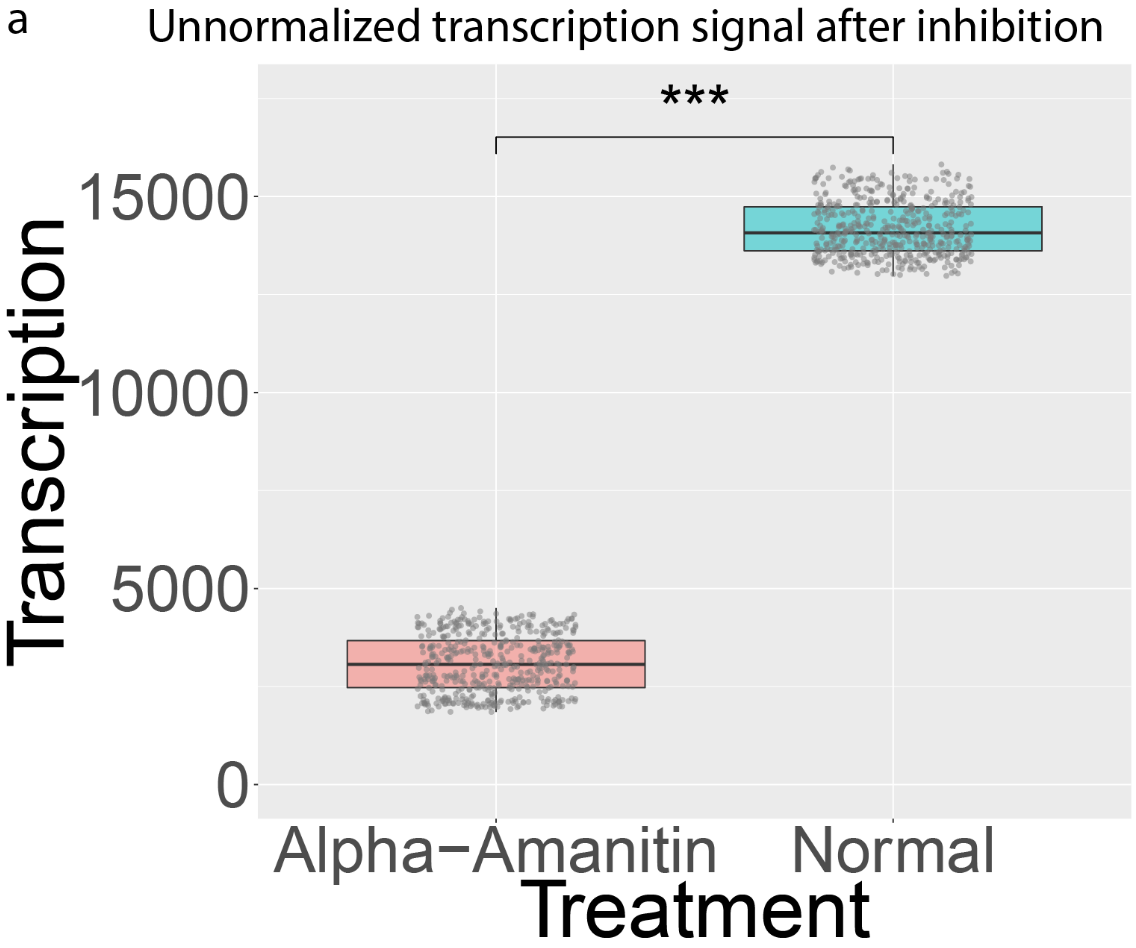
Unnormalized transcription signal after inhibition
***
Transcription
15000
10000
5000
0
Alpha−Amanitin
Normal
Treatment

b

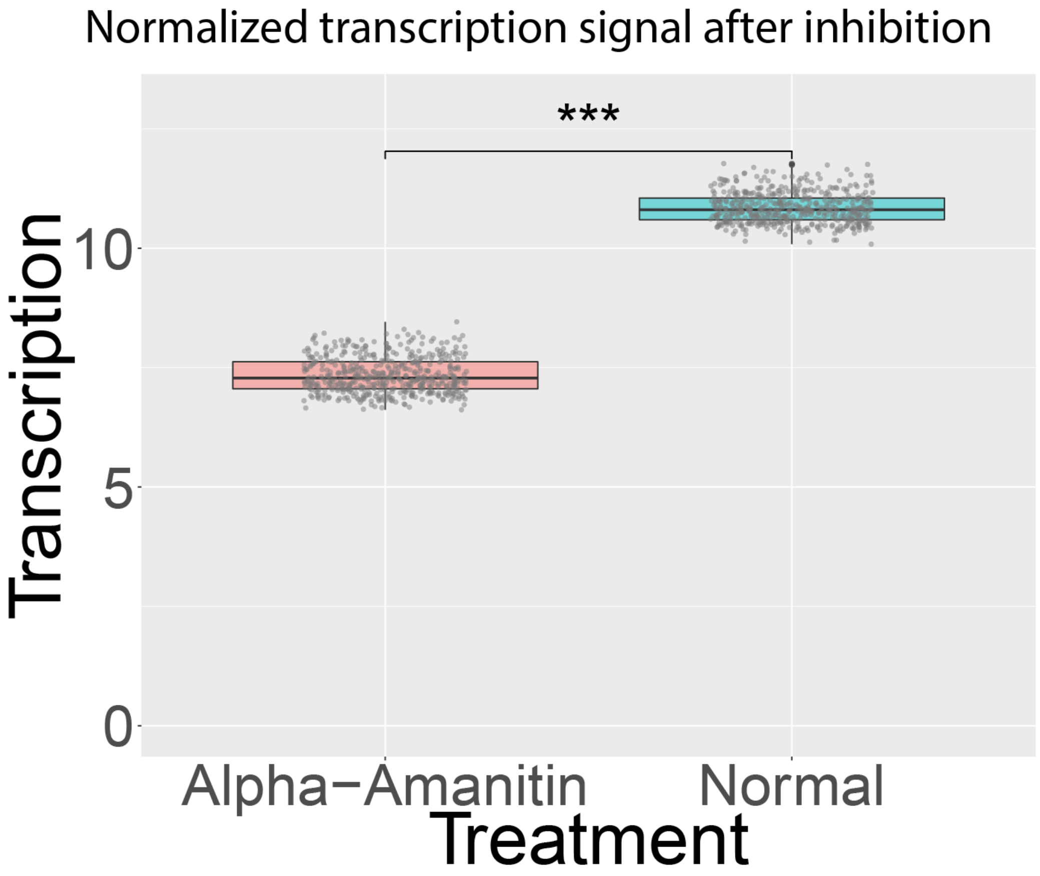
Normalized transcription signal after inhibition
***
Transcription
10
5
0
Alpha−Amanitin
Normal
Treatment

Supplementary Figure 41. Transcription inhibition reduced the nascent transcriptome signal.

a Statistics of total transcription signal at the ^{81}Br-rU channel. Each data point corresponds to a single depth from ion beam tomograms. It exhibited a reduced signal down to the 0.21-fold of the transcription without inhibition. Each scatters point corresponds to a single depth slice from the ion beam tomographic dataset. Box plot includes median, first and third quartile, and 95% confidence interval of the median. The difference in the box plots had a significant p-value < 2.2e-16 *(***)* by the Wilcox test (Two-sided). *n* = 495 slices over a long IBT scan for each transcription condition were included in the analysis.

b Normalization of the transcription signal to the cell area per depth image. This step alleviates the issues of cell size on transcription signal analysis. It showed a reduction down to 0.67-fold of the original transcription signal without inhibition. Each dot is for the single slice in ion tomograms. Box plot indicates the median, first and third quartile, and 95% confidence interval of the median. The difference in the box plots had a significant p-value < 2.2e-16 *(***)* by the Wilcox test (Two-sided). *n* = 495 slices over a long IBT scan for each transcription condition were included in the analysis.

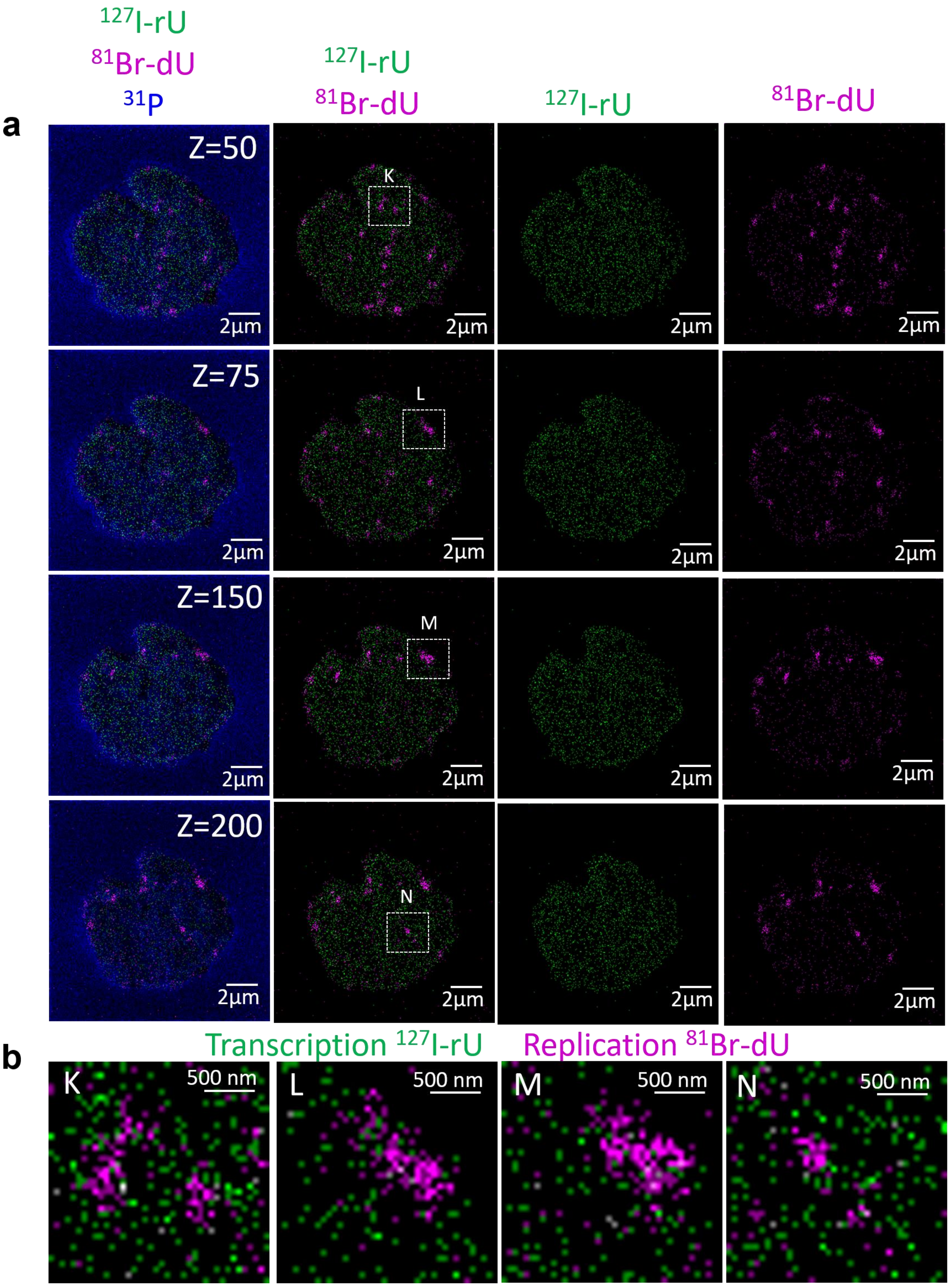

a
b
127I-rU
81Br-dU
31P
127I-rU
81Br-dU
127I-rU
81Br-dU
Z=50
Z=75
Z=150
Z=200
K
L
M
N
2µm
Transcription 127I-rU
Replication 81Br-dU
500 nm

Supplementary Figure 42. IBT results do not depend on the label. The experiment is the same as described in **Supplementary Fig. 36** except that transcripts were labeled with ^{127}I-rU and DNA was labeled with ^{81}Br-dU.

a Ion image for each row corresponds to a 50th, 75th, 150th, and 200th slices.

Note that these images were not processed by the mathematical pipeline, rather raw images were presented to describe spatial differences of individual pixels. 256x256 scans were used at 2 pA imaging current.

b Zoomed images from distinct slices (K, L, M, and N) showed non-overlapping pixels for newly synthesized transcripts and DNAs.

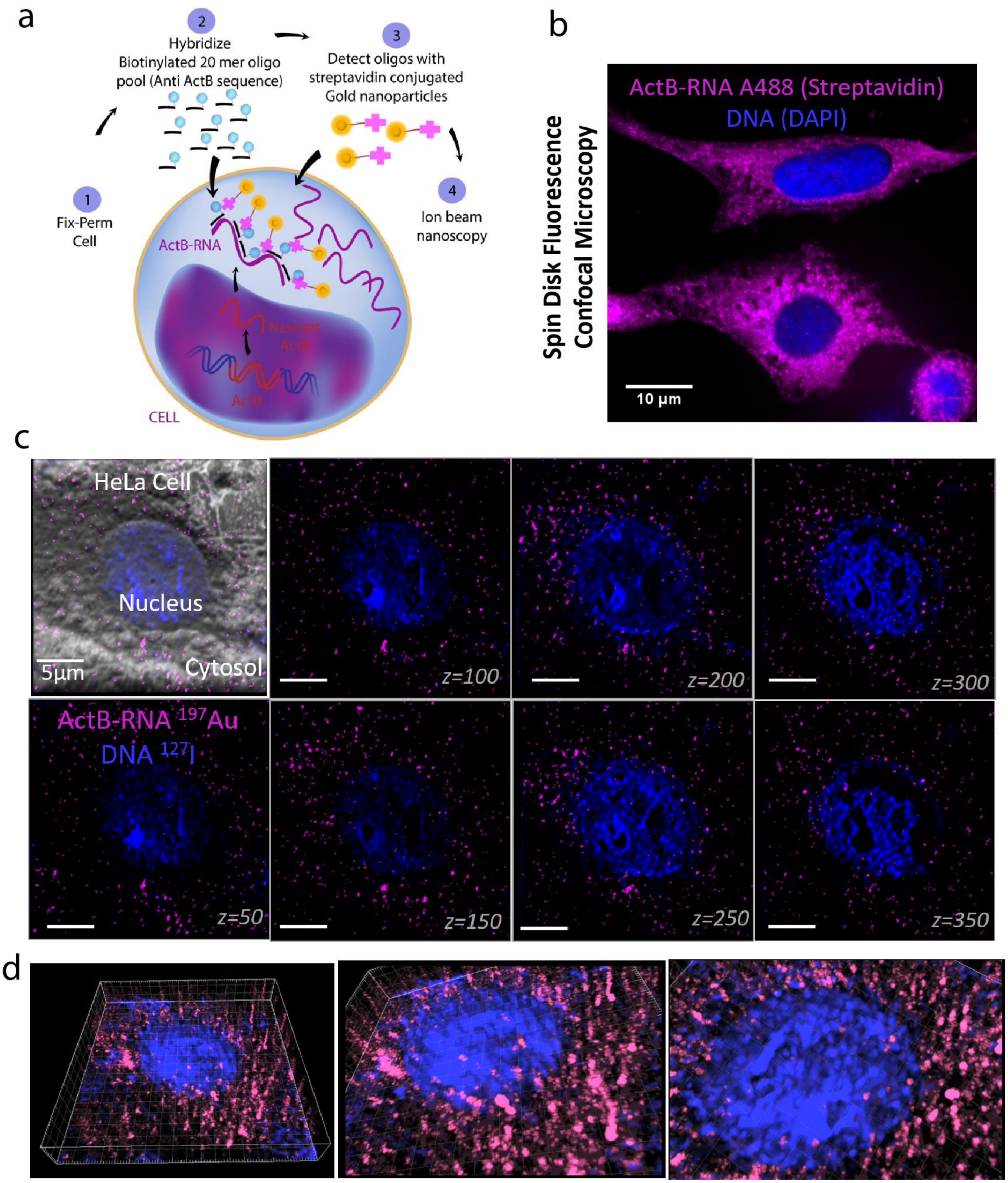

a
2
Hybridize
Biotinylated 20 mer oligo
pool (Anti ActB sequence)
3
Detect oligos with
streptavidin conjugated
Gold nanoparticles
1
Fix-Perm
Cell
4
Ion beam
nanoscopy
ActB-RNA
Nascent
ActB
ActB
CELL
b
Spin Disk Fluorescence
Confocal Microscopy
ActB-RNA A488 (Streptavidin)
DNA (DAPI)
10 μm
c
HeLa Cell
Nucleus
5μm
Cytosol
z=100
z=200
z=300
ActB-RNA 197Au
DNA 127I
z=50
z=150
z=250
z=350
d

Supplementary Figure 43. Ion beam tomography maps transcriptional activity in individual HeLa cells.

a RNA FISH was used to visualize *ActB* mRNA in a HeLa cell. After fixing and permeabilization of the cells, twenty-four biotinylated DNA probes designed to hybridize to the *ActB* mRNA were added to the cells. Streptavidin-conjugated gold nanoparticles were then incubated with the cells. Ion beam imaging was used to detect the probes in the ^{197}Au channel.

b Using streptavidin-A488 secondary conjugate against oligonucleotides, we validated the detection of *ActB* (magenta) in HeLa cells by spin disk fluorescence confocal microscopy. DAPI was used to visualize DNA (blue). Scale bar 10 µm. Most of these transcripts were mature, but we captured a few transcripts in nuclear regions.

c IBT images of *ActB* mRNA in the 25-µm wide central part of a HeLa cell acquired across 450 slices. ^{127}I-dU (blue) was used to label DNA. Scale bars, 5 µm.

d 3D renders of ion beam tomographic RNA images for *ActB* in a single HeLa cell from different viewing angles.

Credit: Image was edited from Alila Medical Media/Shutterstock.com.

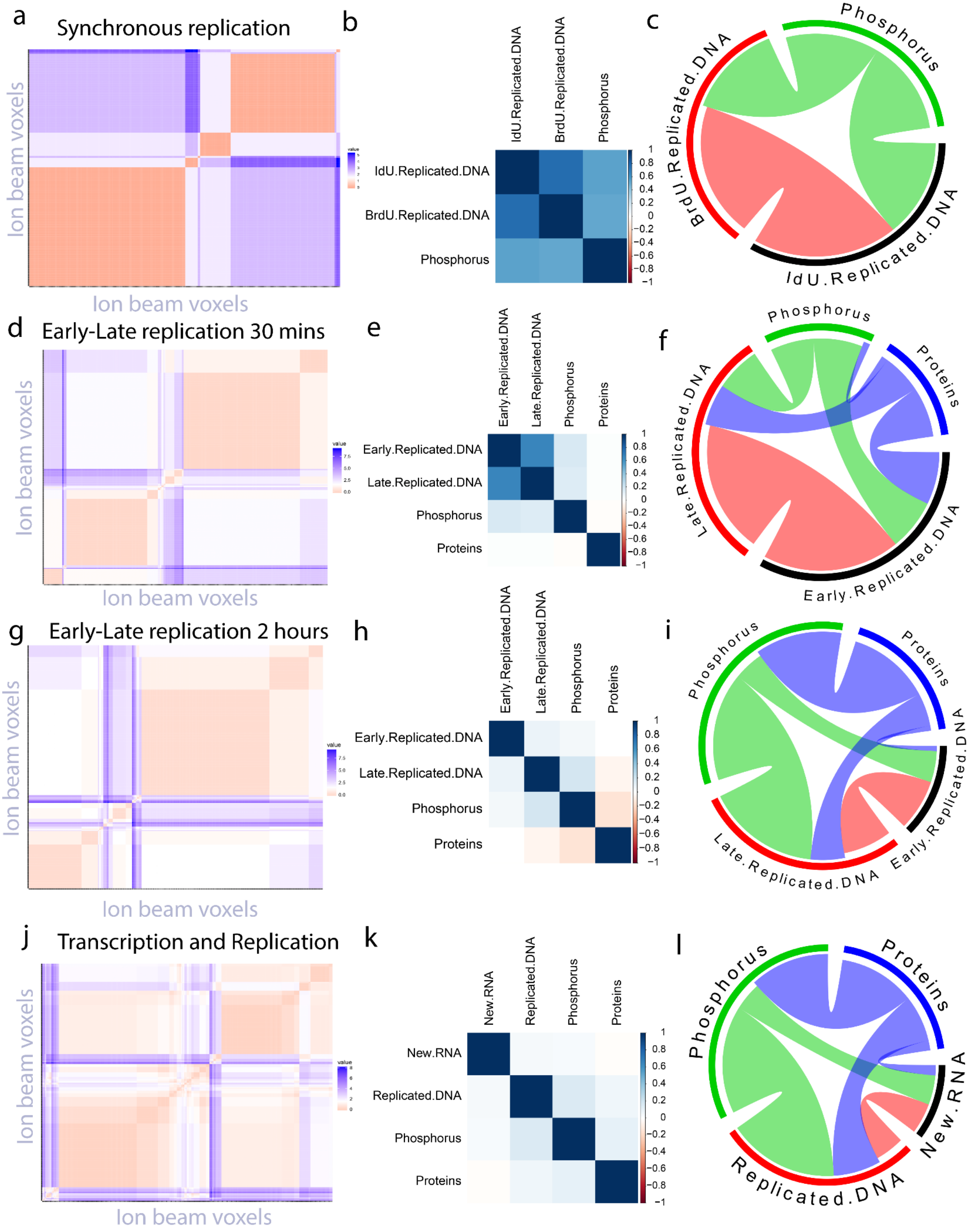
a
Synchronous replication
Ion beam voxels
Ion beam voxels
b
IdU.Replicated.DNA
BrdU.Replicated.DNA
Phosphorus
1 0.8 0.6 0.4 0.2 0 −0.2 −0.4 −0.6 −0.8 −1
c
Phosphorus
BrdU.Replicated.DNA
IdU.Replicated.DNA
d
Early-Late replication 30 mins
Ion beam voxels
Ion beam voxels
e
Early.Replicated.DNA
Late.Replicated.DNA
Phosphorus
Proteins
f
Phosphorus
Proteins
Late.Replicated.DNA
Early.Replicated.DNA
g
Early-Late replication 2 hours
Ion beam voxels
Ion beam voxels
h
Early.Replicated.DNA
Late.Replicated.DNA
Phosphorus
Proteins
i
Phosphorus
Proteins
Early.Replicated.DNA
Late.Replicated.DNA
j
Transcription and Replication
Ion beam voxels
Ion beam voxels
k
New.RNA
Replicated.DNA
Phosphorus
Proteins
l
Phosphorus
Proteins
New.RNA
Replicated.DNA

Supplementary Figure 44. Hierarchical clusters, association, and correlation maps revealed a subcellular structural organization of replicated DNA, synthesized RNA, natural phosphorus, and total proteins from ion beam tomographic information cubes.

Correlation analysis in a HeLa cell with simultaneous incorporation of ^{127}I-dU and ^{81}Br-dU, presented in **Figure 3a**.

a Euclidean distance-based clustered data suggests high degree similarity (strong red color, two significant clusters) between ^{127}I-dU and ^{81}Br-dU images.

b Correlation plot of replicated DNA in ^{127}I-dU and ^{81}Br-dU with phosphorus (^{31}P) channel. ^{127}I-dU and ^{81}Br-dU voxels across 3D cell volume exhibited a high correlation (dark blue). Phosphorous exhibited a slightly lower correlation with ^{127}I-dU and ^{81}Br-dU.

c Chord diagrams showed a higher association of ^{127}I-dU and ^{81}Br-dU compared to the Phosphorus.

Structural organization of early and late replication forks separated by 30 minutes chase in a Nalm6, shown in **Supplementary Fig. 31**.

d Clustered data between 30-min separated ^{127}I-dU and ^{81}Br-dU showed slightly fewer correlation patterns (two significant clusters but smaller size) compared to the synchronous replication-based map, as expected.

e Early and late replication sites remain correlated (lighter blue) with each other, while much less with phosphorus. Total proteins (^{34}S) in the cell partially correlated with replication.

f A direct association between ^{127}I-dU and ^{81}Br-dU (red) exhibited similar circular areas compared to the synchronous replication map in (a). Proteins (blue) interacted with replicated DNAs and partially with phosphorus.

Similar hierarchical mapping of ^{127}I-dU and ^{81}Br-dU separated by 2 hours chase, demonstrated in **Figure 4a**.

g Strong patterns (red) in the clustered map reduced compared to the map from 30-min chase in **d** and blue values suggested higher dissimilarity.

h Now the early-late replication forks are slightly correlated (very light blue) and decent correlation with phosphorous. Proteins were weakly correlated with replication and phosphorus signal.

i Chord diagram showed consistent circular patterns with phosphorous (green), protein (blue), early replicating DNA (black), and late replicating DNA (red). The association between early and late replicated DNA was significantly reduced.

Correlation maps of transcription and replication analysis (pulsed 2 hours) in a Nalm6, detailed in **Figure 5a**.

j Multiple square patterns appeared in the clustered data from ^{127}I-dU (replication) and ^{81}Br-rU (transcription), suggesting isolation of the two.

k Correlation of newly synthesized RNA and replicated DNA is now almost negligible (faint blue). New RNAs are weakly correlated with phosphorus, whereas replicated DNA is correlated with phosphorous signals. Protein exhibited a considerable correlation with others.

l A similar Chord diagram was also obtained as in **i**. New RNAs have an association with replicated DNAs (red) but less the previous association of early-late replicating DNAs in **i**.

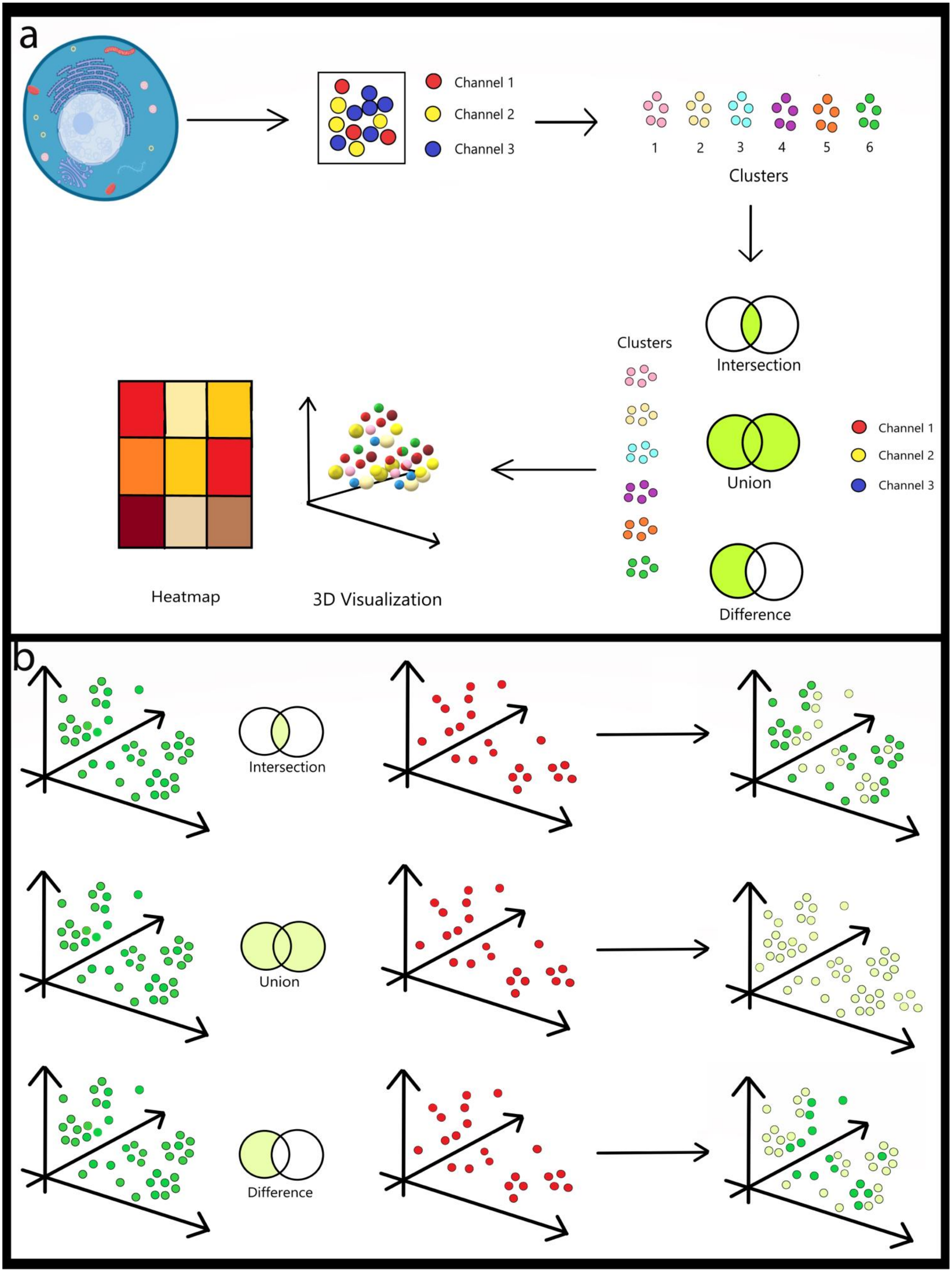
a
Channel 1
Channel 2
Channel 3
1
2
3
4
5
6
Clusters
Clusters
Intersection
Union
Difference
Channel 1
Channel 2
Channel 3
Heatmap
3D Visualization
b
Intersection
Union
Difference

Supplementary Figure 45. Spatially resolved 3D k-means clustering algorithm.

a This flowchart depicts the algorithm used to visualize the 3-dimensional (3D) information acquired from a cell. 3 channels were procured in the form of 3D images, which were in this instance Iodine, Phosphorous, and Bromine. Upon standardizing and normalizing the data in each channel, an unsupervised K-Means clustering algorithm was implemented on the 3 channels which resulted in 6 clusters. Mathematical operations such as intersection, union, and differences were implemented on a variety of combinations between the 6 clusters and 3 channels. This contributed to better 3D visualizations of cell data such as heat maps, density maps, and scatterplots.

b The mathematical operations used in the algorithm for plotting purposes such as intersection, union, and difference are illustrated with visual examples.

(1) The intersection between the green and red clusters results in samples that are common to both clusters. The samples colored light green highlighted in the third figure depicts the points of intersection.

(2) The union between the green and red clusters results in samples that exist in both clusters. The samples colored light green highlighted in the third figure depicts the points of union.

(3) Differences between the green and red clusters result in samples which exist only in the green cluster, but not in the red cluster. The samples colored light green highlighted in the third figure depicts the points of differences.

Credit: Images were created with BioRender.com.

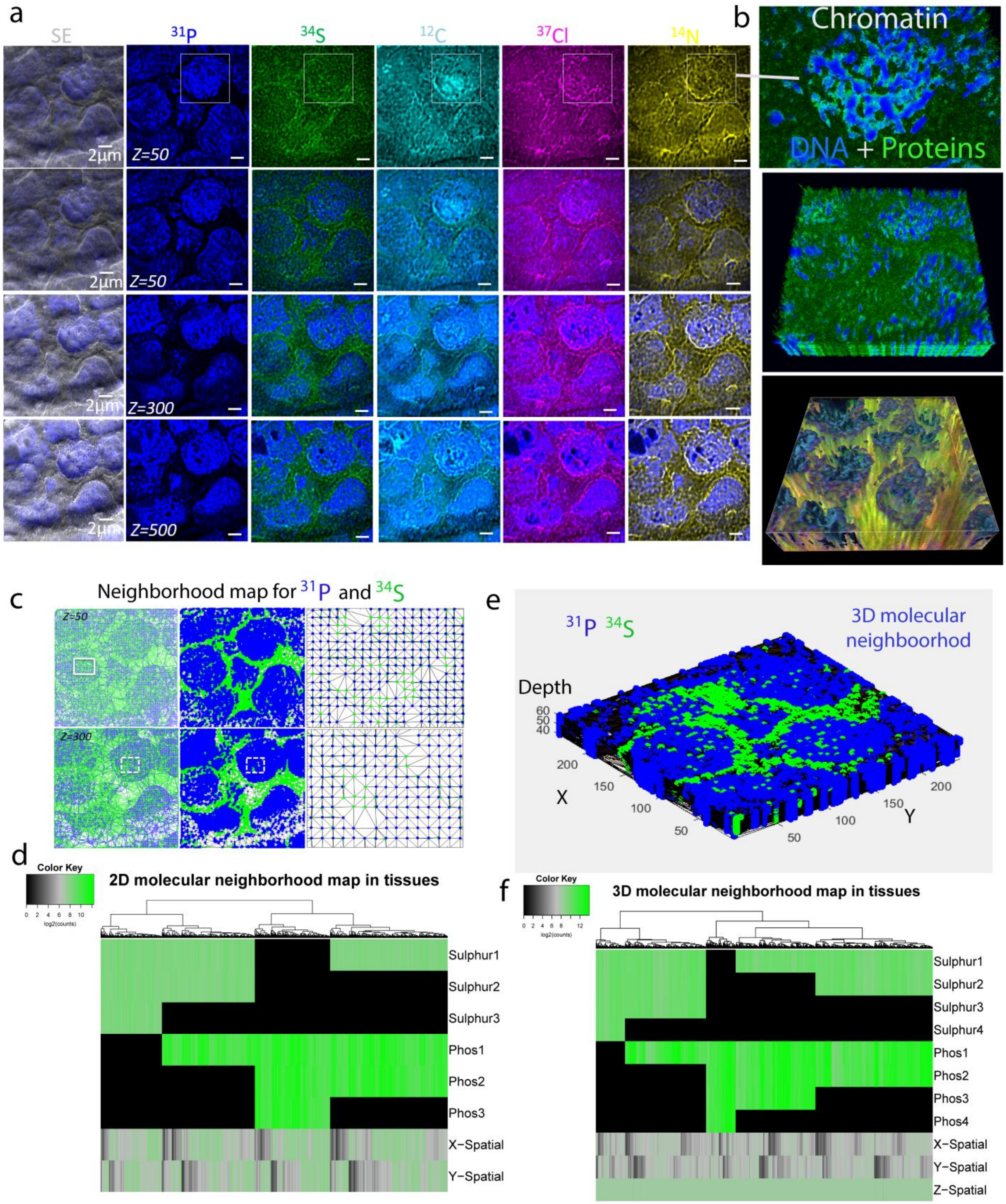
a
SE
^{31}P
^{34}S
^{12}C
^{37}Cl
^{14}N
2µm
Z=50
Z=300
Z=500
b
Chromatin
DNA + Proteins
c
Neighborhood map for ^{31}P and ^{34}S
d
Color Key
log2(counts)
2D molecular neighborhood map in tissues
Sulphur1
Sulphur2
Sulphur3
Phos1
Phos2
Phos3
X−Spatial
Y−Spatial
e
^{31}P ^{34}S
3D molecular neighboorhod
Depth
X
Y
f
3D molecular neighborhood map in tissues
Sulphur4
Phos4
Z−Spatial

Supplementary Figure 46. A molecular neighborhood analysis of chromatin and proteins in FFPE tissues using IBT.

a A label-free ion imaging panel for ^{31}P (primarily chromatin, blue), ^{34}S (proteins, green), ^{12}C (cyan), ^{37}Cl (red), and ^{14}N (yellow) in individual CD3^{+} T cells from a lymph node biopsy core of a T cell lymphoblastic lymphoma patient. Sub-100-nm resolution images were acquired across 500 depth sections. Scale bars, 2 µm. Images in the first column show secondary total ion images as a bright field contrast of the cells. Each row is a 2D representation for 50th, 300^{th,} and 500th slices from ion tomograms.

b IBT renders of single T cells with ^{31}P (blue), ^{34}S (green), and ^{37}Cl (red) co-localization resulting in yellow colors around chromatin islands (indicated by overlapping ^{31}P and ^{34}S, due to the nucleosome).

c Molecular neighborhood map of DNA (^{31}P) and protein (^{34}S) interactions in tissues. Two distinct depth images for 50th and 300th slices were processed by Delaunay triangles that were cornered with blue dots (^{31}P) or green dots (^{34}S).

d Signals of each center-of-mass sub-regions were clustered by heatmap together with spatial positions (X-spatial and Y-spatial) of each triangle. Signals for ^{31}P were highly enriched in the nucleus, and other spatial sub-regions exhibit a combination of ^{31}P and ^{34}S signals. Delaunay triangles were cornered with a combination of phosphate (1-3) sulphur (2-3) signals.

e A 3D neighborhood analysis of twenty sections with Delaunay tetrahedrons that were cornered by four unique green (^{31}P) or magenta (^{34}S) dots.

f Distinct neighborhoods were detected in the heatmap after clustering ^{31}P and ^{34}S with X-Y-Z spatial positions of tetrahedrons. Phosphate (1-4) and Sulphur (1-4) signals corner the tetrahedrons. Chromatin-protein interactions and protein-protein interactions exhibited spatial variations in different clusters.

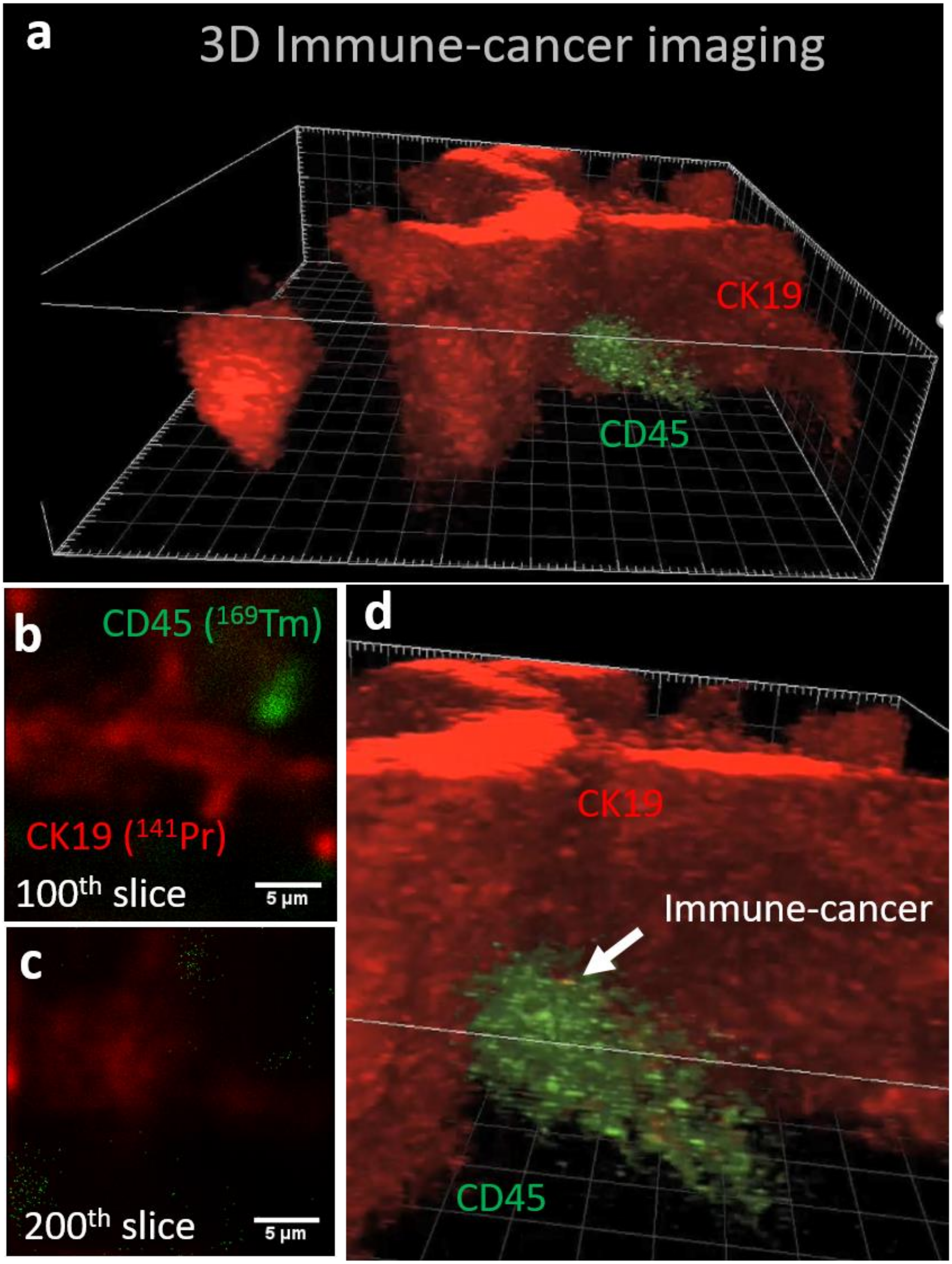
a
3D Immune-cancer imaging
CK19
CD45
b
CD45 (^{169}Tm)
CK19 (^{141}Pr)
100th slice
5 µm
c
200th slice
5 µm
d
CK19
Immune-cancer
CD45

Supplementary Figure 47. Volumetric histology by IBT.

a A sample of cholangiocellular carcinoma from a multi-tumor tissue microarray at 10-µm thickness was mounted on a silicon wafer for ion beam imaging by oxygen ion beam source.

b Immune cell ($CD45^{+}$, label: ^{169}Tm) and cancer cells (CK19, label: ^{141}Pr) were visualized.

c IBT collected at 420 depths allows digital focusing onto different layers (100th and 200th slices shown).

d 3D renders of connected cancer cells (red) and an immune cell (green).

Supplementary Table 1

Sequence
/5Biosg/cgatatcatcatccatggtg
/5Biosg/acataggaatccttctgacc
/5Biosg/cagattttctccatgtcgtc
/5Biosg/atgatctgggtcatcttctc
/5Biosg/cctggatagcaacgtacatg
/5Biosg/catcacgatgccagtggtac
/5Biosg/tcttcatgaggtagtcagtc
/5Biosg/atctcttgctcgaagtccag
/5Biosg/tcattgccaatggtgatgac
/5Biosg/gaaggtagtttcgtggatgc
/5Biosg/tacaggtctttgcggatgtc
/5Biosg/cagtgatctccttctgcatc
/5Biosg/ctcaggaggagcaatgatct
/5Biosg/atctgctggaaggtggacag
/5Biosg/atttgcggtggacgatggag
/5Biosg/agaaagggtgtaacgcaact
/5Biosg/atgccaatctcatcttgttt
/5Biosg/ttcaccgttccagtttttaa
/5Biosg/cctgtaacaacgcatctcat
/5Biosg/gtggacttgggagaggactg
/5Biosg/aaagcaatgctatcacctcc
/5Biosg/actcccagggagaccaaaag
/5Biosg/tcaactggtctcaagtcagt
/5Biosg/cctcatttttaaggtgtgca

Biotinylated DNA sequences for FISH probes that were obtained from IDT company for detecting ActB gene using SIMS and analyzed by IBT.

Supplementary Methods

Tissues

Paraffin blocks of 70 unique different tissues were obtained from the archive of the Institute of Pathology, University of Bern, Switzerland by Christian M. Schürch (C.M.S.), a board-certified pathologist. Cancer and normal tissue regions were annotated on corresponding hematoxylin and eosin-stained sections. A tissue microarray of 70 cores measuring 0.6 mm in diameter was assembled using an automated tissue microarrayer (TMA Grand Master, 3D Histech). Before slicing, silicon substrates were treated with Vectabond (Vector Laboratories) to improve the attachment of tissues on the surface. The tissue microarray block was then sliced onto the silicon substrates (18 mm x 18 mm) at 5-, 10-, and 15-µm thickness. The final silicon samples with tissues were preserved in a vacuum desiccator in the dark. To deparaffinized the tissue, the silicon substrate was manually treated sequentially with xylene (3×), 100% ethanol (2×), 95% ethanol (2×), 80% ethanol, 70% ethanol, and H_2O, each for 30 s. To perform antigen retrieval, the tissue was then placed in a plastic slide holder in target retrieval solution (Agilent, Dako) and loaded into a thermal cycler (PT Module, 97 ^oC for 40 min and cool down to 65 ^oC). After cooling to room temperature, the tissue was rinsed with a wash buffer (PBS 1× IHC with Tween 20 and 0.1% BSA). The sample then was dehydrated and directly imaged by IBT for label-free imaging of subcellular features (**Supplementary Fig. 46**).

Tissue staining

To enable the labeling of mass reporters, after the antigen retrieval step, the tissue on silicon was treated with a blocking buffer (TBS 1× IHC with Tween 20 and 3% donkey serum) for 1 h. After removing the blocking buffer, the antibody mix with CD45 (Label: ^{169}Tm, Cell signaling, and clone: D9M8I) and CK19 (Label: ^{141}Pr, Santa Cruz Biotechnology: sc-6278, and clone: A53-B/A2) was loaded onto the sample and incubated overnight at 4 ^oC. The next day, the sample was rinsed with wash buffer and post-fixed with 2% glutaraldehyde (Electron Microscopy Sciences) for 5 min. After rinsing the sample with low barium PBS, the sample was washed with DI water and dehydrated for ion beam tomographic experiments (**Supplementary Fig. 47)**.
