# Supplementary material for: Nanoscopic subcellular imaging enabled by ion beam tomography
Source: Nat Commun. 2021 Feb 4;12:789. doi: 10.1038/s41467-020-20753-5 (PMC7862654; doi:10.1038/s41467-020-20753-5)
Supplement: Supplementary file 3 — Description of Additional Supplementary Files [file 41467_2020_20753_MOESM3_ESM.pdf]

Description of Additional Supplementary Files

Supplementary Movie 1. 3D IBT reconstruction of a Nalm6 cell treated with Transcription (81Br-rU) and Replication(127I-dU) for 30 min. 3D visuals for the cell that was shown in Figure 2.

Supplementary Movie 2. 3D IBT reconstruction for DNA in a HeLa cell treated with two-colour replication with 127I-dU and 81Br-dU for 24-hours. Only 3D DNA features from 127I-dU were demonstrated. 3D HeLa cell corresponds to - Figure 3.

Supplementary Movie 3. 3D IBT reconstruction for DNA in a HeLa cell treated with two-colour replication with 127I-dU and 81Br-dU for 24-hours. Only 3D DNA features from 81Br-dU were demonstrated. 3D HeLa cell corresponds to Supplementary Figure 30a.

Supplementary Movie 4. 3D IBT reconstruction of a Nalm6 cell treated with 127I-dU and 81Br-dU that was separated by a 30-min chase. The 3D cell patterns correspond to the images that were shown in Figure 4a and b1.

Supplementary Movie 5. 3D IBT visual for replicated DNA patterns that were labelled with 127I-dU and 81Br-dU with a separation of 2-h Chase. 3D Nalm6 cell for the data that was shown in Figure 4b5 and Supplementary Figure 31.

Supplementary Movie 6. 3D IBT representation of a Nalm6 cell that was treated with Transcription (81Br-rU) and Replication(127I-dU) for 2-h. 3D cell data corresponds to Figure 5a2 and Supplementary Figure 38.

Supplementary Movie 7. 3D IBT visual of a Nalm6 cell that was incubated with Transcription (81Br-rU) and Replication(127I-dU together with α-Aminitin transcriptional inhibitor for 30 mins. The cell denotes the images that were shown in Supplementary Figure 39.

Supplementary Movie 8. 3D DNA reconstruction by IBT pipeline for a Nalm6 cell that was treated with two-colour replication with 127I-dU and 81Br-dU for 24-hours. The 3D cell denotes the images that were presented in Supplementary Figure 19.

Supplementary Movie 9. 3D IBT visual for a DNA reconstruction for two-colour replication that was treated with 127I-dU and 81Br-dU that was separated by a 30-min chase. The cell tomograms correspond to Supplementary Figure 32.

Supplementary Movie 10. 3D IBT visual for a DNA reconstruction for two-colour replication that was treated with 127I-dU and 81Br-dU that was separated by a 2-h chase. The 3D cell images correspond to Supplementary Figure 33.

Supplementary Movie 11. 3D IBT reconstruction for a Nalm6 cell that was incubated with Transcription (81Br-rU) and Replication(127I-dU) for 30-minutes. The 3D cell data was obtained from Supplementary Figure 36.

Supplementary Movie 12. 3D mature transcription signals from a HeLa cell using the βAct gene. The single molecule RNA tomograms correspond to the cell that was shown in Supplementary Figure 43.

Supplementary Movie 13. Label free IBT reconstruction of a tissue using 31P (primarily chromatin, blue), 34S (proteins, green), 12C (cyan), 37Cl (red), and 14N (yellow) in individual CD3+ T cells from a lymph node biopsy core of a T cell lymphoblastic lymphoma patient. The 3D tissue images show the cells that was presented in Supplementary Figure 46.

Supplementary Movie 14. IBT reconstruction of a cancer-immune Interface using (CD45+, label: 169Tm) and cancer cells (CK19, label: 141Pr) in a biopsy core from a cholangiocellular carcinoma. The 3D cellular data corresponds to images that were shown in Supplementary Figure 47.

Supplementary Movie 15. Secondary ion beam localization (SILM) working principle is illustrated. A comparison of STORM reconstructed subcellular microtubule patterns and SILM reconstructed DNA images in a HeLa cell was provided. A table was shown for distinct features of STORM and SILM with theoretical localization limits.

Supplementary Software 1. Deconv-IBT analysis package that is used to process the 3D IBT DATA from Raw SIMS Scans. Example data-sets were provided in the form of chromatin and nanotags. The codes were in implemented in MATLAB and Python.

Supplementary Software 2. SILM-IBT analysis package that is used to process the 3D IBT DATA from Raw SIMS Scans. Example data-sets were included as ion image stacks. The codes were processed in Python.

Supplementary Software 3. Deep Learning-IBT analysis package that is used to process the 3D IBT DATA from Raw SIMS Scans in comparison to SILM-IBT reconstructed images. Example data-sets were included as raw and high precision images. The codes were analyzed in Python.

Supplementary Software 4. 3D spatial clustering of IBT data by k-means that is used to process the reconstructed 3D IBT DATA (Deconv-IBT, SILM-IBT, DL-IBT). The code was implemented in Python.

Supplementary Software 5. R quantification of IBT voxel data cubes using heatmap, bar plots with significance, correlation maps, and association circular representations. The extracted excel files from IBT data was analyzed by R-studio interface.

Supplementary Software 6. Resolution analysis of edge scans before and after DECONV-IBT using ERF-function fits in MATLAB.
