# Supplementary material for: Nanoscopic subcellular imaging enabled by ion beam tomography
Source: Nat Commun. 2021 Feb 4;12:789. doi: 10.1038/s41467-020-20753-5 (PMC7862654; doi:10.1038/s41467-020-20753-5)
Supplement: Supplementary file 20 — Reporting Summary [file 41467_2020_20753_MOESM20_ESM.pdf]

nature research

Corresponding author(s): Garry P. Nolan and Ahmet F. Coskun

Last updated by author(s): Nov 21, 2020

Reporting Summary

Nature Research wishes to improve the reproducibility of the work that we publish. This form provides structure for consistency and transparency in reporting. For further information on Nature Research policies, see Authors & Referees and the Editorial Policy Checklist.

Statistics

For all statistical analyses, confirm that the following items are present in the figure legend, table legend, main text, or Methods section.

n/a	Confirmed	
☐	☒	The exact sample size (n) for each experimental group/condition, given as a discrete number and unit of measurement
☐	☒	A statement on whether measurements were taken from distinct samples or whether the same sample was measured repeatedly
☐	☒	The statistical test(s) used AND whether they are one- or two-sided<br>*Only common tests should be described solely by name; describe more complex techniques in the Methods section.*
☐	☒	A description of all covariates tested
☐	☒	A description of any assumptions or corrections, such as tests of normality and adjustment for multiple comparisons
☐	☒	A full description of the statistical parameters including central tendency (e.g. means) or other basic estimates (e.g. regression coefficient) AND variation (e.g. standard deviation) or associated estimates of uncertainty (e.g. confidence intervals)
☐	☒	For null hypothesis testing, the test statistic (e.g. F, t, r) with confidence intervals, effect sizes, degrees of freedom and P value noted<br>*Give P values as exact values whenever suitable.*
☒	☐	For Bayesian analysis, information on the choice of priors and Markov chain Monte Carlo settings
☒	☐	For hierarchical and complex designs, identification of the appropriate level for tests and full reporting of outcomes
☐	☒	Estimates of effect sizes (e.g. Cohen's d, Pearson's r), indicating how they were calculated

Our web collection on statistics for biologists contains articles on many of the points above.

Software and code

Policy information about availability of computer code

Data collection	Acquired original data by NanoSIMS 50L device (Cameca).
Data analysis	Open source codes in Jupyter Notebook(Python 3.7.4 version), MATLAB (2019a), and R (Version 1.1.423, 2009-2018 RStudio, Inc).<br>ImageJ, STORM, and CSB Deep packages were used.<br>The codes used in this IBT pipeline was uploaded to https://github.com/coskunlab/Ion-Beam-Tomography

For manuscripts utilizing custom algorithms or software that are central to the research but not yet described in published literature, software must be made available to editors/reviewers. We strongly encourage code deposition in a community repository (e.g. GitHub). See the Nature Research guidelines for submitting code & software for further information.

Data

Policy information about availability of data

All manuscripts must include a data availability statement. This statement should provide the following information, where applicable:

- Accession codes, unique identifiers, or web links for publicly available datasets
- A list of figures that have associated raw data
- A description of any restrictions on data availability

IBT datasets are available at https://zenodo.org/badge/latestdoi/307904501

October 2018

Field-specific reporting

Please select the one below that is the best fit for your research. If you are not sure, read the appropriate sections before making your selection.

☒ Life sciences ☐ Behavioural & social sciences ☐ Ecological, evolutionary & environmental sciences

For a reference copy of the document with all sections, see nature.com/documents/nr-reporting-summary-flat.pdf

Life sciences study design

All studies must disclose on these points even when the disclosure is negative.

Sample size	Number of cells was more than 20, which was chosen to show repeatability of IBT experiments. Per cell, 3D scans were acquired up to 1,000 cell depth layers and corresponding number of voxels with 256 x 256 x 1,000 data cubes. Similar to the STORM 3D imaging literature, 20 cell with 1,000 z-slices were sufficient to show the statistics of subcellular variations in cancer cells and tissues. These sample sets were adequate to perform control experiments with inhibitors and similarity across datasets.
Data exclusions	No data were excluded.
Replication	At least duplicates of the same condition for cell data were provided. All attempts were successful for replication in dividing/S-phase cells, which was carefully screened during SIMS imaging experiments.
Randomization	Defined spatially organized data based on volumetric regions using replication, transcription, and natural subcellular features. Experimental groups were divided by chase time, pulse time, incubation duration, and the type of the target molecules that were enriched in individual cells. During image acquisition, cells were randomly selected among the dividing/S-phase cells.
Blinding	Direct cellular data with visuals, no blinding was needed.

Reporting for specific materials, systems and methods

We require information from authors about some types of materials, experimental systems and methods used in many studies. Here, indicate whether each material, system or method listed is relevant to your study. If you are not sure if a list item applies to your research, read the appropriate section before selecting a response.

Materials & experimental systems

n/a	Involved in the study	
☐	☒	Antibodies
☐	☒	Eukaryotic cell lines
☒	☐	Palaeontology
☒	☐	Animals and other organisms
☒	☐	Human research participants
☒	☐	Clinical data

Methods

n/a	Involved in the study	
☒	☐	ChIP-seq
☒	☐	Flow cytometry
☒	☐	MRI-based neuroimaging

Antibodies

Antibodies used	(CD45+, label: 169Tm, Cell signaling, and clone: D9M8I) and (CK19, label: 141Pr, Santa Cruz Biotechnology: sc-6278, clone: A53-B/A2) in the Supplementary Figure 47. Streptavidin, Alexa Fluor™ 488 conjugate (with Thermo Fisher Scientific and catalog number: S32354) was used for validation of RNA detection on a confocal microscope.
Validation	Extensively tested in MIBI literature (see Angelo lab, Stanford)<br><br>Specific Validations:<br>CD45 - Keren et al. CELL 2018 (See STAR methods) DOI:https://doi.org/10.1016/j.cell.2018.08.039<br>CK19 - Ohtsuka et al. Sci Rep. 2016 Dec 23;6:39557. DOI: 10.1038/srep39557.<br>Alexa Fluor® 488 streptavidin - Rissman J Clin Invest. 2004;114(1):121-130. https://doi.org/10.1172/JCI20640.

Eukaryotic cell lines

Policy information about cell lines

Cell line source(s)	NALM6 clone G5 (ATCC, CRL-3273) cells , Jurkat clone E6-1 (ATCC® TIB-152™) cells<br>HeLa Cell Line human (Sigma-Aldrich, 93021013, epitheloid cervix carcinoma)

Authentication	None of the cell lines used were authenticated.
Mycoplasma contamination	All cell lines were tested negative for Mycoplasma contamination.
Commonly misidentified lines (See ICLAC register)	No commonly misidentified cell lines were used in this study.
