# Supplementary material for: Nanoscopic subcellular imaging enabled by ion beam tomography
Source: Nat Commun. 2021 Feb 4;12:789. doi: 10.1038/s41467-020-20753-5 (PMC7862654; doi:10.1038/s41467-020-20753-5)
Supplement: Supplementary file 19 — Supplementary Software 1-6 [file 41467_2020_20753_MOESM19_ESM.zip › IBT-SOFTWARE/IBT-software-instructions.pdf]

SOFTWARE PACKAGES IN PYTHON (JUPYTER NOTEBOOK, MATLAB, AND R FILES)

FOR THE ARTICLE, ENTITLED:

Nanoscopic subcellular imaging enabled by Ion Beam Tomography

Ahmet F. Coskun1,2,3,¥,*, Guojun Han1,¥, Shambavi Ganesh3,4, Shih-Yu Chen1, Xavier Rovira Clavé1, Stefan Harmsen2, Sizun Jiang1, Christian M. Schürch1, Yunhao Bai5, Chuck Hitzman6, and Garry P. Nolan1,*
1Baxter Laboratory, Department of Microbiology and Immunology, Stanford University School of Medicine, Stanford, CA
2Department of Radiology, Molecular Imaging Program at Stanford, Stanford University School of Medicine, Stanford, CA
3Wallace H. Coulter Department of Biomedical Engineering, Georgia Institute of Technology and Emory University, Atlanta, GA, USA
4School of Electrical and Computer Engineering, Georgia Institute of Technology, Atlanta, GA, USA
5Department of Chemistry, Stanford University, Stanford, CA
6Department of Materials Science and Engineering, Stanford University, Stanford, CA
¥ Equal contribution
Correspondence should be addressed to
* G.P.N. (gnolan@stanford.edu) and A.F.C. (ahmet.coskun@bme.gatech.edu)

PACKAGE # 1: Deconv-IBT

This package is used to process the 3D IBT DATA from Raw SIMS Scans
Example data-sets: chromatin and nanoTags
MATLAB pipeline: deconvIBT (short slices and 3D batch processing for entire stack)
MATLAB deconvIBTsubfunctions: please add these to directory
Python/Jupyter file for Deconvolution: deconvIBT.ipynb (MATLAB works better, but one can compare)

PACKAGE # 2: SILM-IBT

This package is used to process the 3D IBT DATA from Raw SIMS Scans
Example data-sets: iodrep-3x-all-100 and sequenceiduchromatin
PYTHON/Jupyter pipeline: silm-chromatin-idu (A test series and entire batch processing for 3D IBT stacks)
PYTHON subfolder: storm_analysis make sure to add to your current directory (See here for more comparisons to STORM fluorescence data: https://github.com/ZhuangLab/storm-analysis)

PACKAGE # 3: Deep Learning-IBT

This package is used to process the 3D IBT DATA from Raw SIMS Scans in comparison to SILM-IBT reconstructed images
Example data-sets: IBT-SOFTWARE\DeepLearning-IBT\projection\data
PYTHON/Jupyter pipeline: IBT-SOFTWARE\DeepLearning-IBT\projection and files

deepIBTidu.ipynb (similar ones:deepIBTbrdu.ipynb, deepIBTsi.ipynb, and deepIBTfluo)
PYTHON subfolder: csbdeep and my_model make sure to add to your current directory (See here for more comparisons to Deep learning in fluorescene data: https://csbdeep.bioimagecomputing.com/)

PACKAGE # 4: 3D Spatial clustering of IBT data by k-means

This package is used to process the reconstructed 3D IBT DATA (Deconv-IBT, SILM-IBT, DL-IBT)
Example data-sets: Deconvcell
PYTHON/Jupyter pipeline: MainCodeEditedFiveGTCell11
Example results: Cell11data..., presented in Fig. 6c

PACKAGE # 5: R-STUDIO BASED Quantification of IBT voxel data cubes using heatmap, bar plots with significance, correlation maps, and association circular representations

The extracted excel files from IBT data is analyzed by R interface
BAR PLOTS: IBT-STATS (for replication, transcription, and chromatin overlaps with statistical significance), presented in Fig 4c, 5c and SI Fig. 34a
BAR PLOTS FOR transcription with inhibitors: IBT-STATS with and without normalization, SI Fig. 41 folder
CORRELATION, ASSOCIATION AND HEATMAPS: Circmaps and example plots SI Fig44 a-l
3D LABEL-FREE TISSUE NEIGHBORHOOD ANALYSIS: IBT-STATS-neighborhoods, presented in SI Fig 46

PACKAGE # 6: RESOLUTION ANALYSIS OF EDGE SCANS BEFORE AND AFTER DECONV-IBT USING ERF FITS
RAW DATA, .M Files, And Subfunctions are provided.

06/11/2020 - hope these will be useful and please feel free to ask questions for more details.
