# Supplementary material for: Nanoscopic subcellular imaging enabled by ion beam tomography
Source: Nat Commun. 2021 Feb 4;12:789. doi: 10.1038/s41467-020-20753-5 (PMC7862654; doi:10.1038/s41467-020-20753-5)
Supplement: Supplementary file 19 — Supplementary Software 1-6 [file 41467_2020_20753_MOESM19_ESM.zip › IBT-SOFTWARE/P2 SILM-IBT/storm_analysis/decon_storm/doc/deconSTORM_Conv.html]

Description of deconSTORM\_Conv


Home > deconSTORM\_Conv.m

# deconSTORM\_Conv

## PURPOSE

## SYNOPSIS

**function [sample\_est\_mean, sample\_est\_frames, sample\_est\_hist, saved\_iterations] =deconSTORM\_Conv(mov, APSF, r, niter, iter\_step, alpha, beta, gfactor, fileout, verbose)**

## DESCRIPTION

```
 [sample_est_mean, sample_est_frames, sample_est_hist, saved_iterations] = ...
    deconSTORM_Conv(mov, APSF, r, niter, iter_step, alpha, beta, gfactor, fileout, verbose)

    In 'convolution' mode, APSF is a 2d matrix, the same size as the
    super-resolution image, which will be convolved with the image
    estimate at each iteration.

  mov - fluorescence movie data; 3d array of size pixels x pixels x frames
  APSF - point spread function representation (see above).
  r - background fluorescence intensity
  niter - number of iterations
  iter_step - interval between steps which will be output
  alpha - probability that an active emitter will remain active in the next
           frame
  beta - probability that an inactive emitter will become active in the
           next frame
  gfactor - gain factor
  fileout - name of file in which to store output after every batch of iter_step iterations (optional)
  verbose - 0/1 - whether to print progress reports to the terminal

 Outputs:
  sample_est_mean - Super-resolution estimate of the sample
  sample_est_frames - Super-resolution estimate of each movie frame
  sample_est_hist - history of the estimate during the deconvolution process.
  saved_iterations - vector of the iteration number for each image saved in sample_est_hist
```

## CROSS-REFERENCE INFORMATION

This function calls:


This function is called by:

- demo\_deconSTORM\_microtubules Demo script for deconSTORM, using example fluorescence data from imaging
- demo\_deconSTORM\_simulatedarrow Demo script for deconSTORM, using simulated fluorescence data.

## SUBFUNCTIONS

- function op = cconv2\_PSFk(x,PSFk)

## SOURCE CODE

```
0001 function [sample_est_mean, sample_est_frames, sample_est_hist, saved_iterations] = ...
0002    deconSTORM_Conv(mov, APSF, r, niter, iter_step, alpha, beta, gfactor, fileout, verbose)
0003 %
0004 % [sample_est_mean, sample_est_frames, sample_est_hist, saved_iterations] = ...
0005 %    deconSTORM_Conv(mov, APSF, r, niter, iter_step, alpha, beta, gfactor, fileout, verbose)
0006 %
0007 %    In 'convolution' mode, APSF is a 2d matrix, the same size as the
0008 %    super-resolution image, which will be convolved with the image
0009 %    estimate at each iteration.
0010 %
0011 %  mov - fluorescence movie data; 3d array of size pixels x pixels x frames
0012 %  APSF - point spread function representation (see above).
0013 %  r - background fluorescence intensity
0014 %  niter - number of iterations
0015 %  iter_step - interval between steps which will be output
0016 %  alpha - probability that an active emitter will remain active in the next
0017 %           frame
0018 %  beta - probability that an inactive emitter will become active in the
0019 %           next frame
0020 %  gfactor - gain factor
0021 %  fileout - name of file in which to store output after every batch of iter_step iterations (optional)
0022 %  verbose - 0/1 - whether to print progress reports to the terminal
0023 %
0024 % Outputs:
0025 %  sample_est_mean - Super-resolution estimate of the sample
0026 %  sample_est_frames - Super-resolution estimate of each movie frame
0027 %  sample_est_hist - history of the estimate during the deconvolution process.
0028 %  saved_iterations - vector of the iteration number for each image saved in sample_est_hist
0029 %
0030 
0031 % Copyright, 2012
0032 % Eran Mukamel, Hazen Babcock and Xiaowei Zhuang
0033 % contact: eran@post.harvard.edu
0034 %
0035 
0036 if nargin<10
0037    verbose = 1;
0038 end
0039 if nargin<9
0040    fileout = [];
0041 end
0042 
0043 if verbose
0044    fprintf('********** deconSTORM - Convolution Method ***********\n')
0045 end
0046 
0047 [Lx,Ly,nframes] = size(mov);
0048 
0049 [lx,ly] = size(APSF);
0050 
0051 PSFk = fft2(APSF);
0052 PSFk = repmat(PSFk,[1,1,nframes]);
0053 
0054 dsampx = lx/Lx;
0055 dsampy = ly/Ly;
0056 
0057 % normalization constant
0058 a = sum(APSF(:))/(dsampx*dsampy);
0059 
0060 dsampvecx = [ceil(dsampx/2):dsampx:lx];
0061 dsampvecy = [ceil(dsampy/2):dsampy:ly];
0062 
0063 E = zeros(lx,ly,nframes);
0064 
0065 % define temporal filter
0066 filtord = min(floor(log(.0001)/log(alpha)),100);
0067 expfilt2 = alpha.^abs([-filtord:filtord]);
0068 expfilt2 = expfilt2/sum(expfilt2);
0069 
0070 saved_iterations = unique(find((mod([1:niter],iter_step)==0) | ([1:niter]==niter)));
0071 sample_est_hist = zeros(lx,ly, length(saved_iterations));
0072 sample_est = ones(lx,ly,nframes);
0073 
0074 tic
0075 ll=0;
0076 for jiter = 1:niter
0077    %% deconSTORM iteration
0078    sample_est_down = cconv2_PSFk(sample_est, PSFk);
0079    sample_est_down = sample_est_down(dsampvecx,dsampvecy,:) + r;
0080    EDown = mov ./ sample_est_down;
0081    E(dsampvecx,dsampvecy,:) = EDown;
0082    Econv = cconv2_PSFk(E, PSFk);
0083    
0084    sample_est_curr = padarray(sample_est,[0,0,filtord],0,'pre');
0085    gam = filter(expfilt2, 1, sample_est_curr, [], 3);
0086    gam = gam(:,:,filtord+1:end);
0087    samplecurr = mean(sample_est,3);
0088    samplecurr = repmat(samplecurr,[1,1,nframes]);
0089    gam = min(gam + beta*samplecurr,1);
0090    gam = -log(gam) / gfactor;
0091    
0092    sample_est = sample_est .* Econv ./ (a+gam);
0093    
0094    %% Save intermediate iterations
0095    if (mod(jiter,iter_step)==0) || (jiter==niter)
0096       ll=ll+1;
0097       sample_est_hist(:,:,ll) = squeeze(mean(sample_est,3));
0098       tottime = toc;
0099       if verbose
0100          fprintf('deconSTORM: iteration %d/%d; time=%3.3g ms/iteration/frame; remaining time=%5.5g s.\n',...
0101             jiter,niter, 1000*tottime/jiter/nframes, tottime*(niter-jiter)/jiter)
0102       end
0103       
0104       %% Save output
0105       if ~isempty(fileout)
0106          sample_est_frames = sample_est;
0107          sample_est_mean = mean(sample_est,3);
0108          
0109          save(fileout,'sample_est_mean','sample_est_frames','sample_est_hist','saved_iterations');
0110          if verbose
0111             fprintf('  Saved current output to %s.\n',fileout)
0112          end
0113       end
0114    end
0115 end
0116 
0117 sample_est_hist = reshape(sample_est_hist, lx, ly, ceil(niter/iter_step));
0118 sample_est_frames = reshape(sample_est, lx,ly,nframes);
0119 
0120 % ---------------
0121 function op = cconv2_PSFk(x,PSFk)
0122 %
0123 % Convolution with periodic boundary conditions
0124 %
0125 
0126 if ndims(x)>=3
0127    xk = fft(fft(x,[],1),[],2);
0128    op = ifft(ifft(xk.*PSFk,[],1),[],2);
0129    op = fftshift(fftshift(real(op),1),2);
0130 else
0131    op = fftshift(ifft2(fft2(x).*PSFk,'symmetric'));
0132 end
0133 
0134
```

---

Generated on Sun 25-Mar-2012 15:38:50 by **m2html** © 2003
