# Supplementary material for: Nanoscopic subcellular imaging enabled by ion beam tomography
Source: Nat Commun. 2021 Feb 4;12:789. doi: 10.1038/s41467-020-20753-5 (PMC7862654; doi:10.1038/s41467-020-20753-5)
Supplement: Supplementary file 19 — Supplementary Software 1-6 [file 41467_2020_20753_MOESM19_ESM.zip › IBT-SOFTWARE/P2 SILM-IBT/storm_analysis/decon_storm/doc/deconSTORM_Matrix.html]

Description of deconSTORM\_Matrix


Home > deconSTORM\_Matrix.m

# deconSTORM\_Matrix

## PURPOSE

## SYNOPSIS

**function [sample\_est\_mean, sample\_est\_frames, sample\_est\_hist, saved\_iterations] =deconSTORM\_Matrix(mov, APSF, r, niter, iter\_step, alpha, beta, gfactor, fileout, verbose)**

## DESCRIPTION

```
 [sample_est_mean, sample_est_frames, sample_est_hist, saved_iterations] = ...
    deconSTORM_Matrix(mov, APSF, r, niter, iter_step, alpha, beta, gfactor, fileout, verbose)

 In 'matrix' mode, APSF is a rectangular transfer matrix that
    performs the convolution operation by matrix multiplication.

 Inputs:
  mov - fluorescence movie data; 3d array of size pixels x pixels x frames
  APSF - point spread function representation (see above).
  r - background fluorescence intensity
  niter - number of iterations
  iter_step - interval between steps which will be output
  alpha - probability that an active emitter will remain active in the next
           frame
  beta - probability that an inactive emitter will become active in the
           next frame
  gfactor - gain factor
  fileout - name of file in which to store output after every batch of iter_step iterations (optional)
  verbose - 0/1 - whether to print progress reports to the terminal

 Outputs:
  sample_est_mean - Super-resolution estimate of the sample
  sample_est_frames - Super-resolution estimate of each movie frame
  sample_est_hist - history of the estimate during the deconvolution process.
  saved_iterations - vector of the iteration number for each image saved in sample_est_hist
```

## CROSS-REFERENCE INFORMATION

This function calls:


This function is called by:

- demo\_deconSTORM\_microtubules Demo script for deconSTORM, using example fluorescence data from imaging
- demo\_deconSTORM\_simulatedarrow Demo script for deconSTORM, using simulated fluorescence data.

## SOURCE CODE

```
0001 function [sample_est_mean, sample_est_frames, sample_est_hist, saved_iterations] = ...
0002    deconSTORM_Matrix(mov, APSF, r, niter, iter_step, alpha, beta, gfactor, fileout, verbose)
0003 % [sample_est_mean, sample_est_frames, sample_est_hist, saved_iterations] = ...
0004 %    deconSTORM_Matrix(mov, APSF, r, niter, iter_step, alpha, beta, gfactor, fileout, verbose)
0005 %
0006 % In 'matrix' mode, APSF is a rectangular transfer matrix that
0007 %    performs the convolution operation by matrix multiplication.
0008 %
0009 % Inputs:
0010 %  mov - fluorescence movie data; 3d array of size pixels x pixels x frames
0011 %  APSF - point spread function representation (see above).
0012 %  r - background fluorescence intensity
0013 %  niter - number of iterations
0014 %  iter_step - interval between steps which will be output
0015 %  alpha - probability that an active emitter will remain active in the next
0016 %           frame
0017 %  beta - probability that an inactive emitter will become active in the
0018 %           next frame
0019 %  gfactor - gain factor
0020 %  fileout - name of file in which to store output after every batch of iter_step iterations (optional)
0021 %  verbose - 0/1 - whether to print progress reports to the terminal
0022 %
0023 % Outputs:
0024 %  sample_est_mean - Super-resolution estimate of the sample
0025 %  sample_est_frames - Super-resolution estimate of each movie frame
0026 %  sample_est_hist - history of the estimate during the deconvolution process.
0027 %  saved_iterations - vector of the iteration number for each image saved in sample_est_hist
0028 %
0029 
0030 % Copyright, 2012
0031 % Eran Mukamel, Hazen Babcock and Xiaowei Zhuang
0032 % contact: eran@post.harvard.edu
0033 %
0034 
0035 if nargin<10
0036    verbose = 1;
0037 end
0038 if nargin<9
0039    fileout = [];
0040 end
0041 
0042 if verbose
0043    fprintf('********** deconSTORM - Matrix Method ***********\n')
0044 end
0045 
0046 [Lx,Ly,nframes] = size(mov);
0047 
0048 lx2 = size(APSF,1);
0049 lx = sqrt(lx2); ly = sqrt(lx2);
0050 
0051 % normalization constant for each super-resolution pixel
0052 a = sum(APSF,2);
0053 
0054 % define temporal filter
0055 filtord = min(floor(log(.0001)/log(alpha)),100);
0056 expfilt2 = alpha.^abs([-filtord:filtord]);
0057 expfilt2 = expfilt2/sum(expfilt2);
0058 
0059 saved_iterations = unique(find((mod([1:niter],iter_step)==0) | ([1:niter]==niter)));
0060 
0061 a = a * ones(1,nframes);
0062 mov = reshape(mov, Lx*Ly, []);
0063 
0064 sample_est = ones(size(a));
0065 sample_est_hist = zeros(lx*ly, length(saved_iterations));
0066 
0067 tic
0068 ll=0;
0069 for jiter = 1:niter
0070    %% deconSTORM iteration
0071    
0072    % Estimate of the observed data
0073    sample_est_down = APSF' * sample_est + r;
0074    
0075    EDown = (mov./sample_est_down);
0076    Econv = APSF * EDown;
0077    
0078    sample_est_curr = padarray(sample_est,[0,filtord],0,'pre');
0079    gam = filter(expfilt2, 1, sample_est_curr, [], 2);
0080    gam = gam(:,filtord+1:end);
0081    samplecurr = mean(sample_est,2);
0082    samplecurr = samplecurr*ones(1,nframes);
0083    gam = min(gam + beta*samplecurr,1);
0084    gam = -log(gam) / gfactor;
0085    
0086    sample_est = sample_est .* Econv ./ (a+gam);
0087    
0088    %% Save intermediate iterations
0089    if (mod(jiter,iter_step)==0) || (jiter==niter)
0090       ll=ll+1;
0091       sample_est_hist(:,ll) = squeeze(mean(sample_est,2));
0092       tottime = toc;
0093       if verbose
0094          fprintf('deconSTORM: iteration %d/%d; time=%3.5g ms/iteration/frame; remaining time=%3.3g s.\n',...
0095             jiter,niter, 1000*tottime/jiter/nframes, tottime*(niter-jiter)/jiter)
0096       end
0097       
0098       %% Save output
0099       if ~isempty(fileout)
0100          sample_est_hist = reshape(sample_est_hist, lx, ly, ceil(niter/iter_step));
0101          sample_est_frames = reshape(sample_est, lx,ly,nframes);
0102          sample_est_mean = reshape(mean(sample_est,2),lx,ly);
0103          
0104          save(fileout,'sample_est_mean','sample_est_frames','sample_est_hist','saved_iterations');
0105          if verbose
0106             fprintf('  Saved current output to %s.\n',fileout)
0107          end
0108          
0109          sample_est_hist = reshape(sample_est_hist, lx*ly, ceil(niter/iter_step));
0110       end
0111    end
0112 end
0113 
0114 sample_est_hist = reshape(sample_est_hist, lx, ly, ceil(niter/iter_step));
0115 sample_est_frames = reshape(sample_est, lx,ly,nframes);
```

---

Generated on Sun 25-Mar-2012 15:38:50 by **m2html** © 2003
