# Supplementary material for: Nanoscopic subcellular imaging enabled by ion beam tomography
Source: Nat Commun. 2021 Feb 4;12:789. doi: 10.1038/s41467-020-20753-5 (PMC7862654; doi:10.1038/s41467-020-20753-5)
Supplement: Supplementary file 19 — Supplementary Software 1-6 [file 41467_2020_20753_MOESM19_ESM.zip › IBT-SOFTWARE/P2 SILM-IBT/storm_analysis/decon_storm/doc/deconSTORM_prepareAPSF.html]

Description of deconSTORM\_prepareAPSF


Home > deconSTORM\_prepareAPSF.m

# deconSTORM\_prepareAPSF

## PURPOSE

## SYNOPSIS

**function [APSF] = deconSTORM\_prepareAPSF(sigma,Npixels,npixels)**

## DESCRIPTION

```
 [APSF] = deconSTORM_prepareAPSF(sigma,Npixels,npixels)

 Create a Gaussian point spread function (PSF) convolutional transfer
 function matrix

 Inputs:
 sigma - Standard deviation parameter of the Gaussian PSF shape, in pixels
 Npixels - linear dimension, in pixels, of the image field of view
           (assumed square)
 npixels - Linear dimension (in pixels) of the super-resolution estimate

 Output:
 APSF - transfer function mapping from the high- to the 
 low-resolution images, of size (npixels^2) x (Npixels^2)
```

## CROSS-REFERENCE INFORMATION

This function calls:


This function is called by:

- demo\_deconSTORM\_microtubules Demo script for deconSTORM, using example fluorescence data from imaging
- demo\_deconSTORM\_simulatedarrow Demo script for deconSTORM, using simulated fluorescence data.

## SOURCE CODE

```
0001 function [APSF] = deconSTORM_prepareAPSF(sigma,Npixels,npixels)
0002 %
0003 % [APSF] = deconSTORM_prepareAPSF(sigma,Npixels,npixels)
0004 %
0005 % Create a Gaussian point spread function (PSF) convolutional transfer
0006 % function matrix
0007 %
0008 % Inputs:
0009 % sigma - Standard deviation parameter of the Gaussian PSF shape, in pixels
0010 % Npixels - linear dimension, in pixels, of the image field of view
0011 %           (assumed square)
0012 % npixels - Linear dimension (in pixels) of the super-resolution estimate
0013 %
0014 % Output:
0015 % APSF - transfer function mapping from the high- to the
0016 % low-resolution images, of size (npixels^2) x (Npixels^2)
0017 %
0018 
0019 % Copyright, 2012
0020 % Eran Mukamel, Hazen Babcock and Xiaowei Zhuang
0021 % Contact: eran@post.harvard.edu
0022 %
0023 
0024 dsamp = npixels/Npixels;
0025 
0026 if mod(npixels,Npixels)~=0
0027    error('npixels must be an integer multiple of Npixels.')
0028 end
0029 
0030 xvec = ([1:npixels]-1/2)*Npixels/npixels;
0031 Xvec = ([1:Npixels]-1/2)*Npixels/Npixels;
0032 
0033 [xx,yy] = meshgrid(xvec);
0034 PSF = exp(-((xx-(Npixels+1)/2).^2 + (yy-(Npixels+1)/2).^2)/(2*sigma^2));
0035 
0036 if dsamp>1
0037    dsampvec = [dsamp/2:dsamp:npixels];
0038 elseif dsamp==1
0039    dsampvec = [1:npixels];
0040 end
0041 
0042 norm = sum(sum(PSF(dsampvec,dsampvec)));
0043 
0044 [xx,yy,XX,YY] = ndgrid(xvec,xvec,Xvec,Xvec);
0045 
0046 APSF = exp( -((xx-XX).^2+(yy-YY).^2) / (2*sigma^2) ) / norm;
0047 APSF = reshape(APSF,npixels^2,Npixels^2);
0048
```

---

Generated on Sun 25-Mar-2012 15:38:50 by **m2html** © 2003
