# Supplementary material for: Nanoscopic subcellular imaging enabled by ion beam tomography
Source: Nat Commun. 2021 Feb 4;12:789. doi: 10.1038/s41467-020-20753-5 (PMC7862654; doi:10.1038/s41467-020-20753-5)
Supplement: Supplementary file 19 — Supplementary Software 1-6 [file 41467_2020_20753_MOESM19_ESM.zip › IBT-SOFTWARE/P2 SILM-IBT/storm_analysis/decon_storm/doc/demo_deconSTORM_microtubules.html]

Description of demo\_deconSTORM\_microtubules


Home > demo\_deconSTORM\_microtubules.m

# demo\_deconSTORM\_microtubules

## PURPOSE

**Demo script for deconSTORM, using example fluorescence data from imaging**

## SYNOPSIS

**This is a script file.**

## DESCRIPTION

```
 Demo script for deconSTORM, using example fluorescence data from imaging
 of microtubules.
```

## CROSS-REFERENCE INFORMATION

This function calls:

- deconSTORM\_Conv
- deconSTORM\_Matrix [sample\_est\_mean, sample\_est\_frames, sample\_est\_hist, saved\_iterations] = ...
- deconSTORM\_prepareAPSF
- deconSTORM\_preparePSF

This function is called by:


## SOURCE CODE

```
0001 %
0002 % Demo script for deconSTORM, using example fluorescence data from imaging
0003 % of microtubules.
0004 %
0005 
0006 % Copyright, 2012
0007 % Eran Mukamel, Hazen Babcock and Xiaowei Zhuang
0008 % contact: eran@post.harvard.edu
0009 %
0010 
0011 %% Load sample STORM movie data
0012 
0013 % Fluorescence movie data from imaging of microtubules:
0014 load('deconSTORM_microtubuledata.mat','mov')
0015 
0016 %% Set parameters
0017 
0018 % Npixels is the linear dimension, in pixels, of the image field of view (assumed square)
0019 % nframes is the number of movie frames
0020 [Npixels,~,nframes] = size(mov);
0021 
0022 % Factor by which we will sub-sample each image dimension to create a
0023 % super-resolution sample estimate
0024 dsamp = 8; 
0025 
0026 % Linear dimension (in pixels) of the super-resolution estimate
0027 npixels = Npixels*dsamp;
0028 
0029 % Probability that an active emitter remains active in the next frame
0030 alpha = 1/2.2;
0031 
0032 % Probability that an inactive emitter will become active in the next frame
0033 beta = 6.5e-5;
0034 
0035 % Background fluorescence intensity, in photons per pixel per frame
0036 r = 100;
0037 
0038 % Standard deviation parameter of the Gaussian PSF shape, in pixels
0039 sigma = 1; 
0040 
0041 % Gain parameter for deconSTORM
0042 gfactor = 256;
0043 
0044 %% ---------- METHOD 1: Run deconSTORM using Matrix method
0045 % This method may be computationally faster when there is sufficient memory
0046 % to compute the transfer matrix, APSF. Also, this method does not assume
0047 % periodic boundary conditions.
0048 
0049 % Generate the point spread function transfer matrix, APSF
0050 [APSF] = deconSTORM_prepareAPSF(sigma,Npixels,npixels);
0051 
0052 % Number of iterations of deconSTORM
0053 niter = 1000;
0054 
0055 % Interval between iterations at which to report the output
0056 iter_step = 50;
0057 
0058 % Name of file in which to store results
0059 fileout = 'deconSTORM_simarrow_results.mat';
0060 
0061 verbose = 1; 
0062 
0063 [sample_est_mean, sample_est_frames, sample_est_hist, saved_iterations] = deconSTORM_Matrix(mov, APSF, ...
0064    r, niter, iter_step, alpha, beta, gfactor, fileout, verbose);
0065 
0066 %% ---------- METHOD 1: Run deconSTORM using Convolution method
0067 % This method may be preferrable for large images, for which the transfer
0068 % matrix APSF is too large to store in memory.  This method assumes
0069 % periodic boundary conditions for the image.
0070 
0071 % Generate the point spread function, PSF
0072 [PSF] = deconSTORM_preparePSF(sigma,Npixels,npixels);
0073 
0074 % Number of iterations of deconSTORM
0075 niter = 1000;
0076 
0077 % Interval between iterations at which to report the output
0078 iter_step = 50;
0079 
0080 % Name of file in which to store results
0081 fileout = 'deconSTORM_simarrow_results.mat';
0082 verbose = 1;
0083 
0084 [sample_est_mean, sample_est_frames, sample_est_hist] = deconSTORM_Conv(mov, PSF, ...
0085    r, niter, iter_step, alpha, beta, gfactor, fileout, verbose);
0086
```

---

Generated on Sun 25-Mar-2012 15:38:50 by **m2html** © 2003
