# Supplementary material for: Nanoscopic subcellular imaging enabled by ion beam tomography
Source: Nat Commun. 2021 Feb 4;12:789. doi: 10.1038/s41467-020-20753-5 (PMC7862654; doi:10.1038/s41467-020-20753-5)
Supplement: Supplementary file 19 — Supplementary Software 1-6 [file 41467_2020_20753_MOESM19_ESM.zip › IBT-SOFTWARE/P2 SILM-IBT/storm_analysis/decon_storm/doc/demo_deconSTORM_simulatedarrow.html]

Description of demo\_deconSTORM\_simulatedarrow


Home > demo\_deconSTORM\_simulatedarrow.m

# demo\_deconSTORM\_simulatedarrow

## PURPOSE

**Demo script for deconSTORM, using simulated fluorescence data.**

## SYNOPSIS

**This is a script file.**

## DESCRIPTION

```
 Demo script for deconSTORM, using simulated fluorescence data.
```

## CROSS-REFERENCE INFORMATION

This function calls:

- deconSTORM\_Conv
- deconSTORM\_Matrix [sample\_est\_mean, sample\_est\_frames, sample\_est\_hist, saved\_iterations] = ...
- deconSTORM\_prepareAPSF
- deconSTORM\_preparePSF

This function is called by:


## SOURCE CODE

```
0001 %
0002 % Demo script for deconSTORM, using simulated fluorescence data.
0003 %
0004 
0005 % Copyright, 2012
0006 % Eran Mukamel, Hazen Babcock and Xiaowei Zhuang
0007 % contact: eran@post.harvard.edu
0008 %
0009 
0010 %% Load sample STORM movie data
0011 
0012 % Fluorescence movie data from imaging of microtubules:
0013 load('deconSTORM_simulatedarrowdata.mat','mov')
0014 
0015 %% Set parameters
0016 
0017 % Npixels is the linear dimension, in pixels, of the image field of view (assumed square)
0018 % nframes is the number of movie frames
0019 [Npixels,~,nframes] = size(mov);
0020 
0021 % Factor by which we will sub-sample each image dimension to create a
0022 % super-resolution sample estimate
0023 dsamp = 8; 
0024 
0025 % Linear dimension (in pixels) of the super-resolution estimate
0026 npixels = Npixels*dsamp;
0027 
0028 % Probability that an active emitter remains active in the next frame
0029 alpha = 1/2;
0030 
0031 % Probability that an inactive emitter will become active in the next frame
0032 beta = 1/120;
0033 
0034 % Background fluorescence intensity, in photons per pixel per frame
0035 r =1;
0036 
0037 % Standard deviation parameter of the Gaussian PSF shape, in pixels
0038 sigma = 1; 
0039 
0040 % Gain parameter for deconSTORM
0041 gfactor = 256;
0042 
0043 %% Generate the point spread function (PSF)
0044 
0045 % PSF is a Gaussian point spread function, of size npixels x npixels.
0046 % APSF is a matrix of size (npixels^2) x (Npixels^2). Each column of APSF
0047 % is the expected image (flattened into a column vector) of an emitter at a
0048 % single point in the super-resolution field of view.
0049 
0050 
0051 %% ---------- METHOD 1: Run deconSTORM using Matrix method
0052 % This method may be computationally faster when there is sufficient memory
0053 % to compute the transfer matrix, APSF. Also, this method does not assume
0054 % periodic boundary conditions.
0055 
0056 % Generate the point spread function transfer matrix, APSF
0057 [APSF] = deconSTORM_prepareAPSF(sigma,Npixels,npixels);
0058 
0059 % Number of iterations of deconSTORM
0060 niter = 1000;
0061 
0062 % Interval between iterations at which to report the output
0063 iter_step = 50;
0064 
0065 % Name of file in which to store results
0066 fileout = 'deconSTORM_microtubule_results.mat';
0067 
0068 verbose = 1; 
0069 
0070 [sample_est_mean, sample_est_frames, sample_est_hist, saved_iterations] = deconSTORM_Matrix(mov, APSF, ...
0071    r, niter, iter_step, alpha, beta, gfactor, fileout, verbose);
0072 
0073 %% ---------- METHOD 1: Run deconSTORM using Convolution method
0074 % This method may be preferrable for large images, for which the transfer
0075 % matrix APSF is too large to store in memory.  This method assumes
0076 % periodic boundary conditions for the image.
0077 
0078 % Generate the point spread function, PSF
0079 [PSF] = deconSTORM_preparePSF(sigma,Npixels,npixels);
0080 
0081 % Number of iterations of deconSTORM
0082 niter = 1000;
0083 
0084 % Interval between iterations at which to report the output
0085 iter_step = 50;
0086 
0087 % Name of file in which to store results
0088 fileout = 'deconSTORM_microtubule_results.mat';
0089 verbose = 1;
0090 
0091 [sample_est_mean, sample_est_frames, sample_est_hist] = deconSTORM_Conv(mov, PSF, ...
0092    r, niter, iter_step, alpha, beta, gfactor, fileout, verbose);
0093
```

---

Generated on Sun 25-Mar-2012 15:38:50 by **m2html** © 2003
