# Supplementary material for: Nanoscopic subcellular imaging enabled by ion beam tomography
Source: Nat Commun. 2021 Feb 4;12:789. doi: 10.1038/s41467-020-20753-5 (PMC7862654; doi:10.1038/s41467-020-20753-5)
Supplement: Supplementary file 19 — Supplementary Software 1-6 [file 41467_2020_20753_MOESM19_ESM.zip › IBT-SOFTWARE/P2 SILM-IBT/storm_analysis/decon_storm/doc/index.html]

 Documentation for deconSTORM software 


|  |  |
| --- | --- |
| Master index | Index for . |

# Documentation for deconSTORM software

|  |  |
| --- | --- |
|  | This software is provided as supplementary material for the publication, "Statistical Deconvolution for Super-Resolution Fluorescence Microscopy," by Eran A. Mukamel, Hazen Babcock and Xiaowei Zhuang, *Biophysical Journal* (2012). Please see the paper for a full explanation of the method. |

## Matlab files in this directory:

|  |  |
| --- | --- |
| demo\_deconSTORM\_microtubules | Demo script for deconSTORM, using example fluorescence data from imaging microtubules. |
| demo\_deconSTORM\_simulatedarrow | Demo script for deconSTORM, using simulated fluorescence data. |
| deconSTORM\_Conv | `[sample_est_mean, sample_est_frames, sample_est_hist, saved_iterations] =   deconSTORM_Conv(mov, APSF, r, niter, iter_step, alpha, beta, gfactor, fileout, verbose)` |
| deconSTORM\_Matrix | `[sample_est_mean, sample_est_frames, sample_est_hist, saved_iterations] =   deconSTORM_Matrix(mov, APSF, r, niter, iter_step, alpha, beta, gfactor, fileout, verbose)` |
| deconSTORM\_prepareAPSF | `APSF = deconSTORM_prepareAPSF(sigma,Npixels,npixels)` |
| deconSTORM\_preparePSF | `PSF = deconSTORM_preparePSF(sigma,Npixels,npixels)` |

## Other Matlab-specific files in this directory:

- **deconSTORM\_microtubuledata.mat** - Example microtubule fluorescence data
- **deconSTORM\_simulatedarrowdata.mat** - Example simulated fluorescence data

---

© 2012 Eran Mukamel, Hazen Babcock and Xiaowei Zhuang

contact: eran at post dot harvard dot edu

Generated on Sun 25-Mar-2012 15:38:49 by **m2html** © 2003
