# Supplementary material for: Nanoscopic subcellular imaging enabled by ion beam tomography
Source: Nat Commun. 2021 Feb 4;12:789. doi: 10.1038/s41467-020-20753-5 (PMC7862654; doi:10.1038/s41467-020-20753-5)
Supplement: Supplementary file 19 — Supplementary Software 1-6 [file 41467_2020_20753_MOESM19_ESM.zip › IBT-SOFTWARE/P5 R - Quantification/SI Fig 41/SI Fig 41a AlphaAminTreat-Unnormalized.pdf]

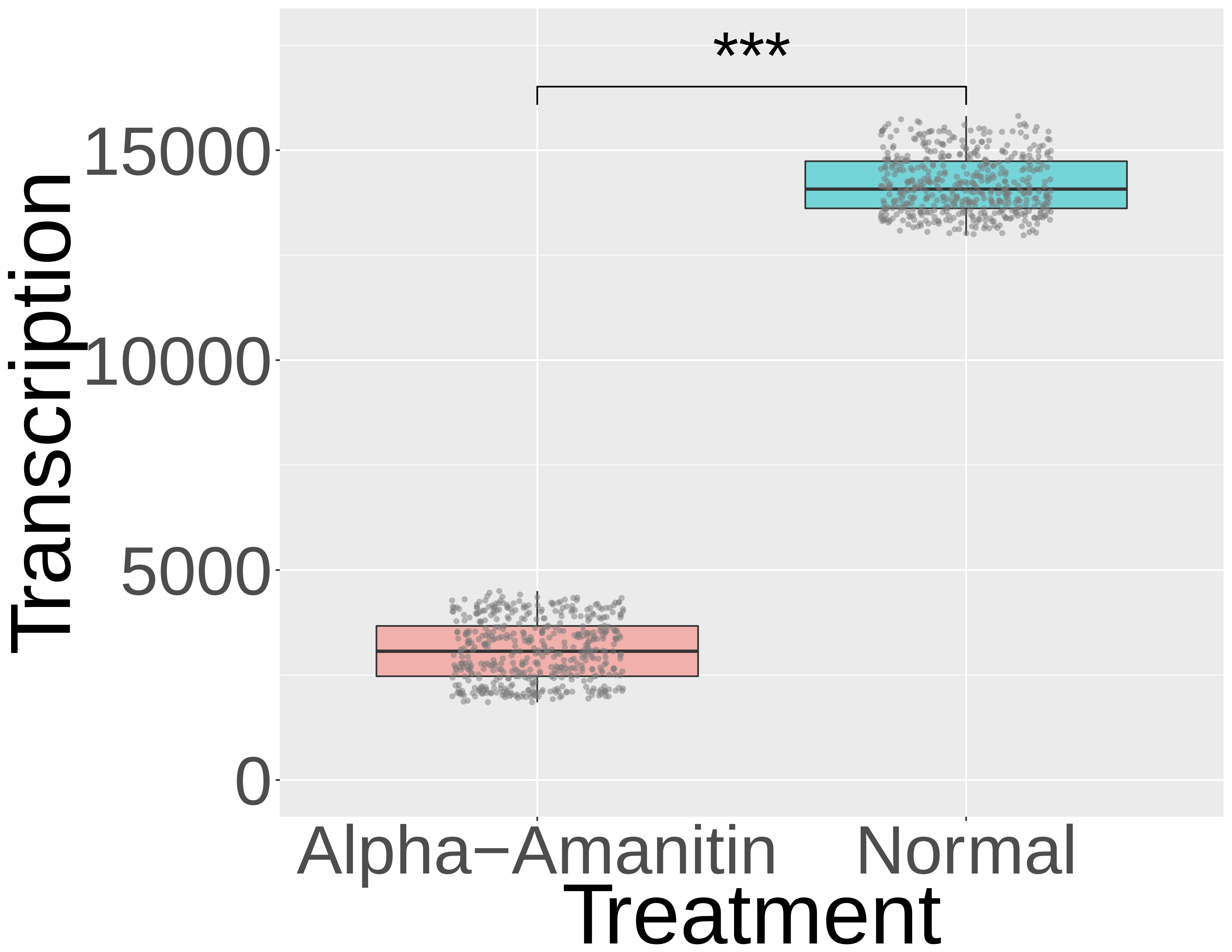
***
15000
10000
5000
0
Transcription
Alpha−Amanitin
Normal
Treatment
