# Supplementary material for: Nanoscopic subcellular imaging enabled by ion beam tomography
Source: Nat Commun. 2021 Feb 4;12:789. doi: 10.1038/s41467-020-20753-5 (PMC7862654; doi:10.1038/s41467-020-20753-5)
Supplement: Supplementary file 19 — Supplementary Software 1-6 [file 41467_2020_20753_MOESM19_ESM.zip › IBT-SOFTWARE/P5 R - Quantification/SI Fig 44/SI Fig 44h.pdf]

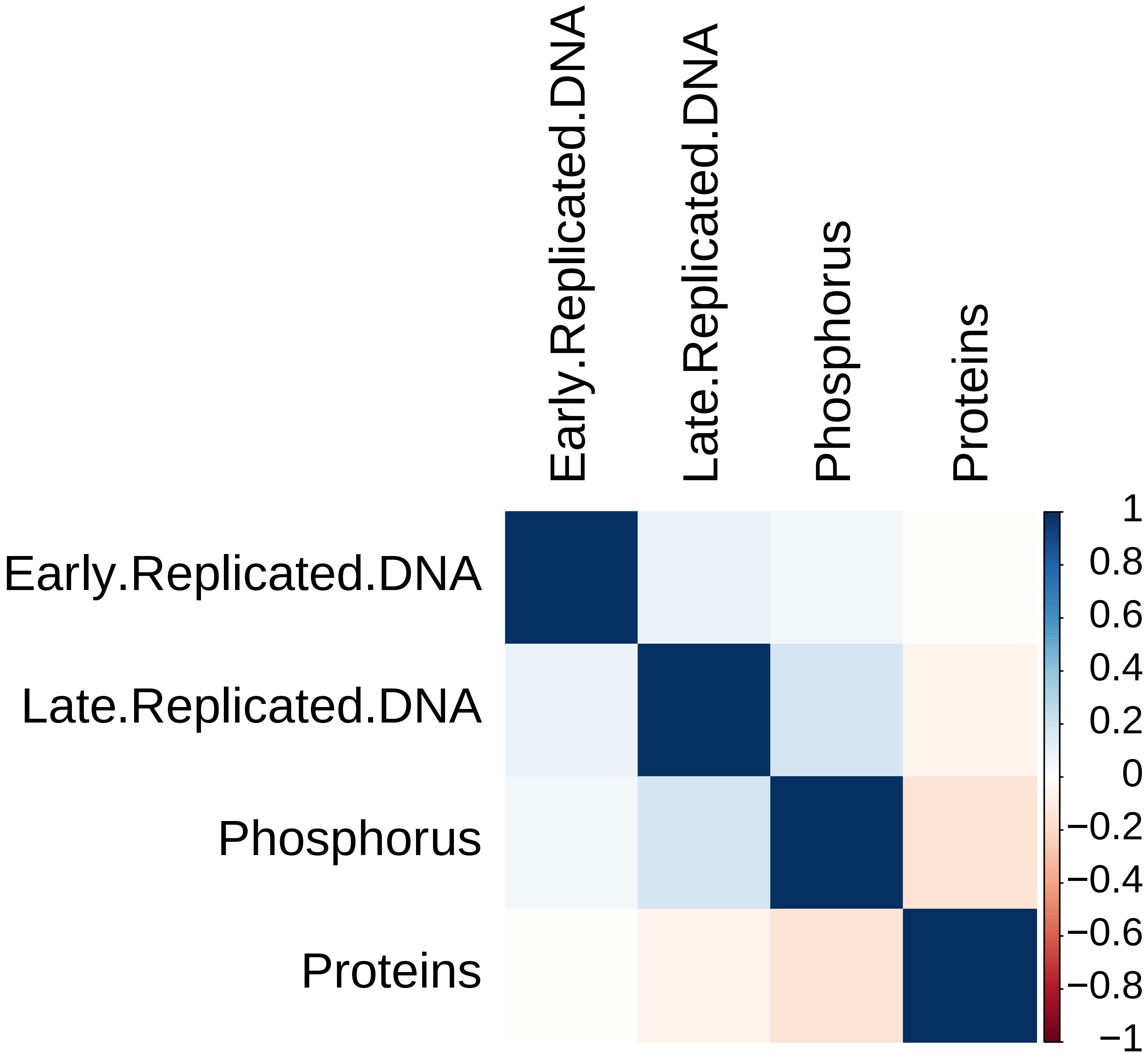

Early.Replicated.DNA
Late.Replicated.DNA
Phosphorus
Proteins
Early.Replicated.DNA
Late.Replicated.DNA
Phosphorus
Proteins
1
0.8
0.6
0.4
0.2
0
−0.2
−0.4
−0.6
−0.8
−1
